# Biochemical characterization of a cyanobactin arginine-*N*-prenylase from the autumnalamide biosynthetic pathway†

**DOI:** 10.1039/d2cc01799g

**Published:** 2022-09-28

**Authors:** Claudia Clemente, Nicholas Johnson, Xiaodan Ouyang, Rafael V. Popin, Sergio Dall'Angelo, Matti Wahlsten, Jouni Jokela, Alessandro Colombano, Brunello Nardone, David P. Fewer, Wael E. Houssen

**Affiliations:** Institute of Medical Sciences, University of Aberdeen Ashgrove Road West Foresterhill Aberdeen AB25 2ZD UK w.houssen@abdn.ac.uk; Department of Microbiology, University of Helsinki, P.O.Box 56, Viikki Biocenter Viikinkaari 9 00014 Finland david.fewer@helsinki.fi; CEM Microwave Ltd, Buckingham Industrial Park Buckingham MK18 1WA UK; Department of Chemistry, University of Aberdeen, Meston Walk Aberdeen AB24 3UE UK

## Abstract

Cyanobactins are linear and cyclic post-translationally modified peptides. Here we show that the prenyl-d-Arg-containing autumnalamide A is a member of the cyanobactin family. Biochemical assays demonstrate that the AutF prenyltransferase targets the guanidinium moiety in arginine and homoarginine and is a useful tool for biotechnological applications.

## Abstract 2

Biochemical characterization of the prenyltransferase (AutF) from the autumnalamide pathway shows it targets the nitrogen of the guanidinium moiety in arginine and homoarginine.

## Graphical abstract

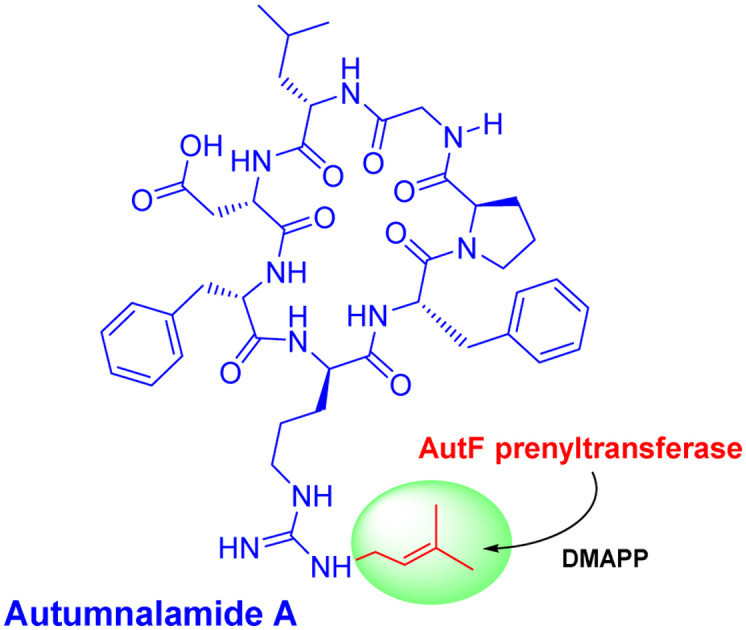

Cyclic peptides can modulate therapeutic targets that involve extended binding surfaces such as protein–protein interactions that are challenging for small molecule drugs.^1–3^ Cyclic peptides often show increased target binding affinity and improved chemical stability against proteolytic enzymes and thereby display a longer biological half-life than their linear counterparts.^4^ However, cyclic peptides are under-exploited due to their poor cellular permeability and negligible oral bioavailability.^1^ Increasing lipophilicity of peptide-based drugs *via* lipidation or prenylation can enhance their cellular permeability.^5^ Prenyltransferases catalyse the regioselective and chemoselective intramolecular transfer of prenyl groups from an isoprene donor to an electron rich position on an aromatic ring or heteroatom in an acceptor molecule.^6^ They are commonly reported from the biosynthetic pathways of cyanobactins which are linear and cyclic peptides produced through the post-translational modification of precursor peptides.^7^ Cyanobactin post-translational modifications include N-to-C macrocyclization,^8^ heterocyclization to generate thiazolines and oxazolines;^9^ oxidation of heterocycles to thiazoles and oxazoles;^10^ and prenylation.^11–19^ Cyanobactin prenyltransferases belong to the ABBA superfamily of prenyltransferases^20^ and catalyse the *O*-prenylation of Tyr, Thr and Ser in the forward or reverse orientation,^12,18^ the forward prenylation of Trp indole on C3 and N1,^13,14,19^ and the prenylation at the N- or C-terminus of linear peptides.^21,22^ Cyanobactins containing reverse *O*-prenylated Tyr undergo a Claisen rearrangement to yield forward *C*-prenylated Tyr.^18^

The AgcF prenyltransferase from the argicyclamide biosynthetic pathway was recently reported to catalyse the mono- and bis-*N*-prenylation of the guanidine moiety of l-Arg.^17^ The argicyclamide A scaffold consists otherwise exclusively of l-amino acids.^17^ AgcF was found to have high substrate selectivity while being capable of using dimethylallyl diphosphate (DMAPP) or geranyl diphosphate (GPP) as a prenyl or geranyl donor.^17^

Autumnalamide A (Fig. 1) is an *N*^ω^-d-arginine mono-prenylated cyclic peptide that was isolated from the organic extract of the cyanobacterium *Phormidium autumnale* CCAP1446/10^23^ and proved to prevent IL2 production from T cells through the modulation of store operated calcium channels.^24^ The autumnalamide A chemical structure was determined using NMR and HRMS while the absolute configuration was determined using chiral GC MS analysis and ^13^C NMR.^23^ This analysis has revealed that autumnalamide A contains d-Pro and prenylated d-Arg residues.

**Fig. 1:**
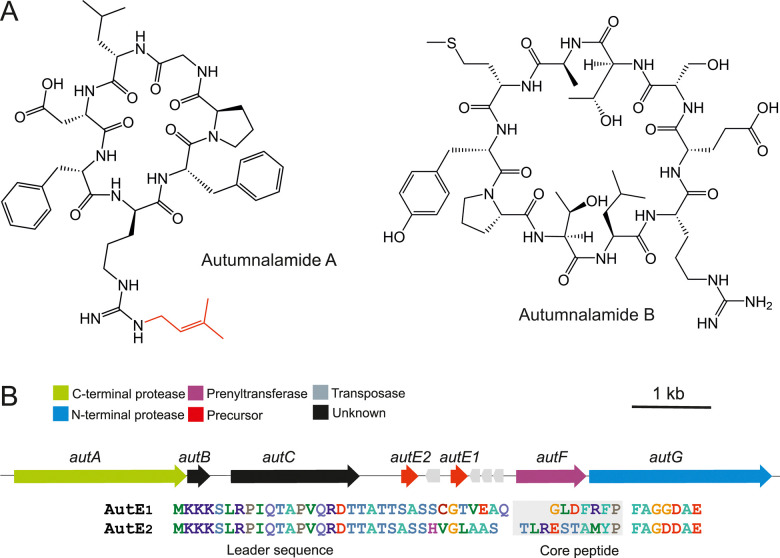
(A) Chemical structures of autumnalamide A and B produced by the cyanobacterium *Phormidium autumnale* CCAP1446/10. (B) The autumnalamide biosynthetic pathway encodes AutE1 and AutE2 precursor peptides (core sequences are highlighted in gray) that are modified to autumnalamide A and B respectively.

Here, we identify the autumnalamide (*aut*) biosynthetic pathway and characterize the substrate specificity of the AutF prenyltransferase.

We extracted high-molecular-weight genomic DNA from *Phormidium autumnale* CCAP1446/10 and obtained a 6.74 Mb draft genome sequence. We predicted a 9.6 kb cyanobactin biosynthetic gene cluster through tBLASTn searches using biosynthetic enzymes from the anacyclamide biosynthetic pathway^14^ as query sequences against a standalone BLAST database. This putative autumnalamide (*aut*) biosynthetic gene cluster encoded seven cyanobactin biosynthetic proteins organized in a single operon (Fig. 1). Surprisingly, the *aut* biosynthetic gene cluster encodes two precursor proteins, AutE1 and AutE2, and a cyanobactin prenyltransferase, AutF (Fig. 1 and Table S2, ESI†). The core sequence of AutE1 matched the sequence of the previously reported autumnalamide A exactly^23^ (Fig. 1). LC-MS analysis of the cyanobacterial extract also showed that autumnalamide A is the most abundant cyanobactin produced while trace amount of non-prenylated autumnalamide A can be detected (Fig. S2 and Table S1, ESI†).

We used LC-MS analysis to identify the predicted product of the AutE2 precursor peptide. The major product detected was a new non-prenylated cyclic peptide that we named autumnalamide B (Fig. 1 and Fig. S2, Table S1, ESI†). Trace amount of the mono-prenylated autumnalamide B was detected (Table S1, ESI†).

The stereochemistry of autumnalamide B was determined through comparison of retention time over multiple runs in HRLC-MS analysis and MS fragmentation pattern with four synthetic stereochemical variants containing all possible combination of l- or d-Arg and l- or d-Pro 1–4 (Table 1) and with Marfey analysis. This analysis showed that autumnalamide B exclusively consists of l-amino acids (Fig. S3–S5, ESI†).

**Table 1:** Summary of the *in vitro* prenylation assays catalysed by AutF

Sample	Sequence^a^	Prenylation/(% yield)^b^	Fig.	Sample	Sequence^a^	Prenylation/(% yield)^b^	Fig.
1	Cyclo[-TLRESTAMYP]	<1.5	S9	13	H-FDLGPFR-NH_2_	<1.5	S21
2	Cyclo[-TLrESTAMYp]	<1.5	S10	14	H-FDLGPFr-NH_2_	<1.5	S22
3	Cyclo[-TLRESTAMYp]	<1.5	S11	15	H-FREDLGPAYD-NH_2_	<1.5	S23
4	Cyclo[-TLrESTAMYP]	<1.5	S12	16	H-FRADLGPAYD-NH_2_	<1.5	S24
5	H-TLRESTAMYPFQA-NH_2_	<1.5	S13	17	*N*-α-Boc-l-arginine	<1.5	S25
6	Cyclo[-LGPFrFD]	N	S14	18	*N*-α-Fmoc-l-arginine	<1.5	S26
7	Cyclo[-LGPFRFD]	4	S15	19	Z-l-arginine-OH·HCl	<1.5	S27
8	H-FrFDLGpAYD-NH_2_	<1.5	S16	20	Z-d-arginine-OH·HCl	<1.5	S28
9	H-FrFDLGPAYD-NH_2_	<1.5	S17	21	Fmoc-l-homoarginine-OH	30	S29
10	H-FRFDLGPAYD-NH_2_	30	S18	22	H-FXFDLGPAYD-NH_2_ where X is l-homoarginine	<1.5	S30
11	H-RFDLGPF-NH_2_	N	S19	23	H-QYLDEKLPNG-NH_2_	N	S31
12	H-rFDLGPF-NH_2_	N	S20	24	H-VVKGALKSLV-NH_2_	N	S32

a The core sequences of autumnalamide A and B are “GLDFRFP” and “TLRESTAMYP” respectively.

b Percentage yield was calculated by dividing the chromatographic peak area of the charged species by the sum of peak areas of prenylated product and/or the remaining substrate in the reaction mixture.

The *autF* gene was then cloned in pEHISTEV-SUMO vector (Gift from Dr Haunting Liu) in frame with an N-terminal Tobacco etch virus (TEV) protease-cleavable His_6_SUMO tag, expressed in *E. coli* BL21(DE3) and purified (Fig. S8, ESI†). We used a series of 24 linear and macrocyclic peptides and protected amino acids to test the substrate specificity of AutF (Table 1). Some linear peptides were synthesised with a C-terminus three amino acid recognition signals for the macrocyclases PatG_mac_^8*a*^ (Ala-Tyr-Asp) and PCY1^25^ (Phe-Gln-Ala) in order to prepare cyclic peptide substrates.

Substrates 1–4 are four variants of autumnalamide B containing “l- or d-Arg” and “l or d-Pro”. AutF catalysed the mono-prenylation of these substrates with poor yield (<1.5%) and this correlates well with the fact that the majority of the naturally detected autumnalamide B is non-prenylated (Fig. S9–S12, ESI† and Table 1). AutF also prenylates the linear peptide substrate 5 containing the autumnalamide B sequence with a C-terminus PCY1 macrocyclase recognition signal but with overall poor yield <1.5% (Fig. S13, ESI† and Table 1).

AutF could not process substrate 6 which is variant of the autumnalamide A core with the only difference of containing l-Pro instead of d-Pro (Fig. S14, ESI† and Table 1). The AutF enzyme was capable of catalysing, albeit with low yield, the mono-prenylation of cyclic peptide 7 which is another autumnalamide A variant containing l-Arg and l-Pro instead of d-Arg and d-Pro respectively (Fig. S15, ESI† and Table 1). However, the yield of the prenylated product of AutF reaction with substrate 7 was 4% which does not correlate with the fact that autumnalamide A constitute the major autumnalamide in the cyanobacterial extract (Table S1, ESI†). Therefore, we tested the ability of AutF to process linear variants of autumnalamide A.

AutF prenylates linear peptide 8 containing the autumnalamide A core sequence with a C-terminus PatG_mac_ recognition signal but with again overall poor yield <1.5% (Fig. S16, ESI† and Table 1). Similarly, the yield was very low in case of substrate 9 which is the same sequence as substrate 8 but containing l-Pro instead of d-Pro (Fig. S17, ESI† and Table 1). However, the yield of the prenylation product increased 29-fold when using substrate 10 containing the autumnalamide A sequence with both l-Arg and l-Pro instead of d-Arg and d-Pro and a C-terminus PatG_mac_ signal (Fig. S18, ESI† and Table 1).

Based on these results, we predict that the prenylation step in autumnalamide A biosynthesis precedes the epimerization step. We could not identify an epimerase-encoding gene in the autumnalamide biosynthetic cluster or more distantly within the draft genome. This is reminiscent of patellamides and kawaguchipeptins in which the genetic origin of epimerization has not yet been discovered and it was proposed to be chemically spontaneous.^26,27^

Substrates 11–14 are permutations of the core peptide sequence of autumnalamide A in AutE1 (Fig. 1) but with d- or l-Arg at either the N-terminus or the C-terminus. AutF was not able to process substrates 11 and 12 containing l- and d-Arg respectively at the N-terminus (Fig. S19 and S20, ESI† and Table 1) while it was able to process, with very low yield, substrates 13 and 14 which contain l- and d-Arg at the C-terminus respectively (Fig. S21 and S22, ESI† and Table 1). This suggests that AutF is sensitive to the immediate environment surrounding the Arg residue in linear peptides with high preference for the Arg to be sandwiched between two residues. The same requirement was previously reported for the C-3 tryptophan prenyltransferase KgpF from the kawaguchipeptin biosynthetic pathway.^16^

The fact that the autumnalamide B core sequence was not prenylated suggested that the enzyme is sensitive to the presence of the acidic “Glu” that precedes the Arg in autumnalamide B. To test this hypothesis, we synthesized substrate 15, which is a homolog of substrate 10 but with the replacement of one of the Phe residues surrounding the Arg with Glu. AutF mono-prenylates this substrate but with very low (<1.5%) yield (Fig. S23, ESI† and Table 1).

In order to check if the enzyme would accept small amino acid instead of one of the Phe residues in substrate 10, we synthesized and tested substrate 16 and MS analysis of the reaction revealed that the enzyme mono-prenylates this substrate again with low (<1.5%) yield (Fig. S24, ESI† and Table 1). These results suggest that AutF processes peptides in which Arg is sandwiched between bulky hydrophobic residues.

AutF also showed very weak ability (<1.5% yield) to mono-prenylate *N*-α-Boc-l-Arg (17), *N*-α-Fmoc-l-Arg (18), Z-l-Arg-OH (19) and Z-d-Arg-OH (20) (Fig. S25–S28, ESI† and Table 1). However, although AutF could not process the homoarginine-containing peptide substrate 22 (Fig. S30, ESI†), it can process Fmoc-l-homoarginine-OH (21) with 30% yield (Fig. S29, ESI†). This makes AutF a useful tool to produce prenylated Fmoc-l-homoarginine that could be used in solid phase peptide synthesis of homoarginine-containing peptides.

In addition, we tested the ability of AutF to prenylate the basic amino acid, lysine in the peptide substrates 23 and 24. MS analysis of the biochemical reactions shows that AutF could not process these substrates (Fig. S31 and S32, ESI† and Table 1) and thus we can conclude that the enzyme is specifically targeting the guanidine moiety in Arg and homoarginine.

AutF steady state kinetic parameters were determined using substrates 10 (*K*_cat_/*K*_m_ = 7.18 S^−1^ M^−1^) and 21 (*K*_cat_/*K*_m_ = 10.21 S^−1^ M^−1^) due to their relatively higher conversion yield (Fig. S33, ESI†). The optimal reaction conditions for AutF were determined by calculating the turnover number (*K*_cat_) with substrate 21 under different reaction conditions (Fig. S34, ESI†). The results indicate that the higher turnover is achieved at pH 7 in the presence of 500 mM NaCl and Mg^2+^ as a metal ion. These conditions were used to scale up the enzymatic reaction with Fmoc-homoarginine (21) to obtain sufficient amount of prenylated product for structural characterization by NMR. The ^1^H–^1^H COSY spectrum shows a correlation between 16-NH and 17-H which ascertains that the prenylation occurs on the N^ω^ of the homoarginine guanidinium moiety (Fig. S35a–o and Table S5, ESI†).

Interestingly, AutF did not accept GPP as an isoprene donor because we did not detect any geranylated product from its GPP-containing reaction with substrate 10. On the other hand, AgcF, from argicyclamide pathway, was reported to accept both DMAPP and GPP and was capable of catalysing the mono-geranylation of argicyclamide C.^17^ AutF shares 62% sequence identity with AgcF (Fig. S36, ESI†). To gain insight into the reason of AutF inability to use GPP as a donor, we generated computational models for both AgcF and AutF using the crystal structure of PagF (PDB: 5TU6) with which they share 42% and 44% sequence similarity respectively (Fig. S37, ESI†). The residues forming the active site entrance in both AgcF (Cys218, Cys266, Leu288, Gly66, Gly132) and AutF (Cys219, Cys267, Leu288, Gly67, Ala133) are conserved with the only difference is the replacement of Gly132 in AgcF with the slightly larger Ala133 in AutF (Fig. S37, ESI†). Schmidt and Nair groups recently demonstrated that a single amino acid change (F222G or F222A) in the isoprene-binding pocket of the *O*-Tyr prenyl transferase, PagF, completely switched its donor specificity from DMAPP to GPP.^28^ We used site directed mutagenesis to generate AutF A133G variant. This protein was expressed, purified and tested for its ability to process substrate 10 with both DMAPP and GPP. Results indicate that this variant can only use DMAPP as cofactor donor. Structural studies are then required to fully understand the substrate/cofactor specificity of both AutF and AgcF. This is because the binding of the substrate may induce conformation changes in the active site that create more space to accommodate longer cofactor. This has been noticed in the bacterial indole prenyltransferases; TleC from *Streptomyces blastmyceticus* and MpnD from *Marinactinospora thermotolerans* which are known to catalyze the prenylation of (−)-indolactam V at the C-7 position of the indole ring with GPP or DMAPP, to produce lyngbyatoxin or pendolmycin, respectively. Structural data revealed that TleC-specific Trp97 rotates by about 70° when the substrates are bound to the active site. This rotation generates a bigger space to accommodate the long side chain of the C10 GPP. This space is lacking in the C5 prenyltransferase MpnD as the rotation of the corresponding residue Tyr80 is not allowed due to the steric hinderance between Tyr80 and Met159.^29^

In this study, we identified the autumnalamide biosynthetic cluster and identified a new product. In addition, we biochemically characterized the prenyl transferase AutF, which targets the guanidine moiety in arginine and homoarginine. Interestingly, the enzyme catalyses the linear l-Arg containing variant of autumnalamide A with much higher yield than the cyclic variants containing l- or d-Arg. We predict that prenylation precedes the epimerization. However, this requires additional studies to be confirmed.

W. E. H. and D. P. F. designed research; C. C., N. J., X. O., R. V. P., S. D., M. W., J. J., A. C., B. N., D. P. F. and W. E. H. performed experiments and analysed data; W. E. H., D. P. F., J. J. and M. W. wrote the manuscript with contribution from all the authors.

This project was supported by a fellowship grant from the EPSRC (No. EP/S027246/1, W.E.H.), a training grant from the BBSRC (BB/V509206/1, W. E. H. and S. D.), the Novo Nordisk Foundation (18OC0034838, D. P. F.) and the NordForsk NCoE program “NordAqua” (Project Number 82845, D. P. F.). C. C. is funded by a PhD studentship from University of Aberdeen. N. J. is funded by the IBioIC CTP PhD programme. R. V. P. was funded by the Doctoral Programme in Microbiology and Biotechnology of the University of Helsinki. X. O. was funded by the China Scholarship Council (Grant 201906150148). We are grateful to Professor James Naismith (University of Oxford) for sharing the construct used to express the macrocyclase PCY1. We thank Dr Huanting Liu (University of St Andrews) for sharing the pEHISTEV-SUMO vector.

## Conflicts of interest

There are no conflicts of interest to declare.

## Supplementary Material

CC-058-D2CC01799G-s001
