# Supplementary material for: Biochemical characterization of a cyanobactin arginine-N-prenylase from the autumnalamide biosynthetic pathway
Source: Chem Commun (Camb). 2022 Sep 28;58(86):12054–7. doi: 10.1039/d2cc01799g (PMC9609003; doi:10.1039/d2cc01799g)
Supplement: CC-058-D2CC01799G-s001 [file CC-058-D2CC01799G-s001.pdf]

Electronic Supplementary Material (ESI) for ChemComm.
This journal is © The Royal Society of Chemistry 2022

Supporting Information

Biochemical Characterization of a Cyanobactin Arginine-*N*-prenylase from the Autumnalamide Biosynthetic Pathway

Claudia Clemente, Nicholas Johnson, Xiaodan Ouyang, Rafael V Popin, Sergio Dall'Angelo, Matti Wahlsten, Jouni Jokela, Alessandro Colombano, Brunello Nardone, David P Fewer & Wael E Houssen**

Supporting Materials and Methods

Cultivation of the cyanobacterial CCAP1446/10 strain, DNA extraction and genome sequencing and assembly

Phormidium autumnale CCAP1446/10 was purchased from the Culture Collection of Algae and Protozoa and purified to remove bacterial and fungal contaminants. The strain was grown in photon irradiance of 5 µmol m^{-2} s^{-1} in Z8 medium for 28 d at 25°C and the resulting biofilm was harvested using a glass rod. Genomic DNA of the strain was extracted from cells that were lysed with a heat-shock treatment consisting of repeated (x15) liquid nitrogen immersion and thawing at 55 °C water bath. DNA extraction was then carried out using a commercial DNA extraction kit (E.Z.N.A.® SP Plant DNA Mini Kit Protocol - Fresh/Frozen Samples, Omega Bio-Tek). DNA-yield and quality were verified by NanoDrop (NanoDrop 1000 Spectrophotometer, Thermo Fisher Scientific). The DNA size and quality were further assessed by gel electrophoresis at room temperature (100 V, 400 mA, 30 min, 0.9% agarose) in 0.5 × TAE-buffer (20 mM Tris, 10 mM acetic acid, 0.5 mM EDTA, pH 8.3).

Libraries were prepared with Nextera DNA flex library prep kit (recently renamed to Illumina DNA Prep) and Illumina MiSeq sequencing was carried out using the MiSeq Reagent Kit v3 (600 cycle). Obtained sequences were trimmed to remove adapters using Trimmomatic v0.39 [S1] and the assemblies were prepared from the trimmed fastaq files using SPAdes v3.12.0 with the –careful option.[S2] The resulting assembly was then further processed for taxonomic classification using Kraken v2 [S3] and contaminating scaffolds were removed with ZEUSS v1.0.2.[S4] Lastly, scaffolding and gap closing were done using Platanus 1.2.4 [S5] to yield a 6.74 Mb assembly with 171 scaffolds.

16S ribosomal RNA phylogenetic analysis

A phylogenetic tree based on the 16S ribosomal RNA gene was generated using 75 previously published cyanobacterial sequences selected from representative cyanobacterial orders to illustrate the phylogenetic position of *Phormidium autumnale* CCAP1446/1 (Figure S1). The 76 sequences were aligned with MUSCLE alignment in MEGA11 using default parameters.[S6] A default BIC calculation with the program jModelTest v2.1.2 indicated that the evolutionary model HKY+I+G best fitted the data set.[S7] This model was used for the construction of a Bayesian inference using the program MrBayes v3.2.7a with 5,000,000 generations and default number of runs and chains.[S8] The posterior probabilities were calculated by the Markov chain Monte Carlo method implemented in the program. The visualization of the tree and collapsing of the clades was made in FigTree v1.4.4 (http://tree.bio.ed.ac.uk/software/figtree/) and figure optimization was performed with Inkscape 1.1 (https://inkscape.org).

Detection of autumnalamide

Cells were collected from the 40 mL culture of *Phormidium autumnale* CCAP1446/10 by centrifugation at 7,000*g* for 7 min (Eppendorf Centrifuge 5804 R). The collected cells were freeze dried (Christ® Beta 2 – 8 LSCplus, LyoCub e 4 – 8) yielding 10 – 55 mg. Freeze dried

cells were extracted in 1 mL 100 % methanol and homogenized using 200 µL of cell disruption media (0.5 mm glass beads, Scientific industries, Inc.) by mechanical crushing with a Fastprep cell disruptor (Bio 101, Thermo Electron Corporation, Qbiogene, Inc.) at a speed of 6.5 ms^{-1} for 45 s. Suspensions were centrifuged at 20,000 *g* for 5 min, filtered through 0.2 µm filter (13 mm syringe filter, PTFE, VWR international) and analyzed by mass spectrometry (MS).

Samples were analyzed with LC-MS (Waters Acquity I-class UPLC and QTOF, SYNAPTG2 -Si, Waters) (Figure S2, Table S1). The mobile phase consisted of solutions A (0.1 % solution of formic acid in water) and B (0.1 % solution of formic acid in acetonitrile/2-propanol (1/1)). Two methods were used. In the first method, the column was Kinetex® C8 (LC Column (50 x 2.1 mm, 1.7 µm 100 Å, Phenomenex) and gradient at the beginning of analysis consisted of 5 % liquid B which was increased linearly to 100% in 5 min, liquid B was kept at 100 % until 7 min, and from 7.1 to 10 min the proportion of liquid B was 5%. In the second method, the column was the same, but the length was 100 mm. Gradient at the beginning of analysis consisted of 5 % liquid B which was increased linearly to 100% in 10 min, liquid B was kept at 100 % until 14 min, and from 14.1 to 20 min the proportion of liquid B was 5 %. The injection volume varied from 0.1 to 1 µL.

QTOF was calibrated using sodium formate and Ultramark® 1621. Leucine Enkephalin was used at 10 s intervals as a lock mass reference compound. Data were accumulated in positive electrospray ionization Resolution Mode at scan range of *m/z* 50-2000. Additional MS parameters were as follows: Polarity, ES+; capillary, 3.0 kV; source temperature, 120 °C; sampling cone, 40.0; source offset, 80.0; source gas flow, 0.0 mL /min; desolvation temperature, 600 °C; cone gas flow, 50.0 L/h. Identification of autumnalamide B was based on the theoretical molecular weights calculated from the predicted core peptide from candidate strains using ChemBioDraw.

Stereochemistry of autumnalamide B was determined by comparing its retention time with those of the different synthetic stereochemical variants (**1**-**4**) (Figure S3). Selected ion recording (SIR) chromatograms were carried out by injecting each of the synthetic peptide stereochemical variants **1**-**4** six times and autumnalamide B from *Phormidium autumnale* CCAP1446/10 extract three times into Waters Acquity Premier UPLC system equipped with PDA UV detector and QDa mass detector and recorded the retention time in each run. In addition, we compared the MS fragmentation pattern of autumnalamide B in the extract with those of the synthetic stereochemical variants **1**-**4** (Figure S4). Marfey analysis of purified autumnalamide B was also carried out (Figure S5).

Prediction and annotation of autumnalamide biosynthetic gene cluster

The 9.6-kb autumnalamide (*aut*) biosynthetic gene cluster was predicted through tBLASTn [S9] searches using AcyA, AcyG and AcyF protein sequences from the anacyclamide biosynthetic gene cluster [S10] as query sequences against a standalone BLAST database of the *Phormidium autumnale* CCAP1446/10 draft genome (Table S2). The genes encoded in the *aut* biosynthetic gene cluster were predicted using GLIMMER as implemented in Artemis.[S11] Start sites were predicted and proteins annotated manually using a combination of searches against the Conserved Domain Database and protein classification resources at NCBI and InterProScan searches and BLASTp[S9] searches against the non-redundant database at NCBI (Table S2). The annotated sequence of the *aut* biosynthetic gene cluster from *Phormidium autumnale* CCAP1446/10 was deposited in GenBank under accession number JAIGNI000000000.

The AutF prenyltransferase was aligned with 36 other cyanobactin prenyltransferases using MUSCLE (https://www.ebi.ac.uk/Tools/msa/muscle/). 127 positions were retained and used for the construction of a phylogenetic tree using PHYML (Figure S6). A phylogenetic tree was constructed using the WAG amino acid substitution model, four substitution rate categories, an estimated proportion of invariable sites of 0.013, and an estimated γ-distribution shape parameter of 2.415. The stability of the in-group relations was assessed with 1000 bootstrap

replicates. The resulting phylogenetic tree was rooted using midpoint rooting using RETREE and visualized using TREEVIEW.

The AutF prenyltransferase was to query the nonredundant database at NCBI. This analysis identified three complete cyanobactin biosynthetic pathways that encoded close homologs of the AutF prenyltransferase from the anacyclamide and piricyclamide biosynthetic pathway (Figure S7A). Inspection of these cyanobactin biosynthetic gene clusters identified 2-4 precursor proteins that encode either Lys or Arg in the core region (Figure S7B).

Recombinant tagged AutF protein sequence (MWt: 46.97 kDa):

MGSSHHHHHHGSDSEVNQEAKPEVKPEVKPETHINLKVSDGSSEIFFKIKKTTPLRRLMEAF
AKRQGKEMDSLRFLYDGIRIQADQTPEDLDMEDNDIIEAHREQIGGDIPTTENLYFQGTTLLE
NSKKLYYIGIHKRVFEVENLYPLDVFENFVEQIEKTSENCILESSCKIEQDKLYPARFDIGFKL
QNLEQLNAVYNFFQKVEARADVRVNHSLIQQFFGEDFDFSKMTGFMVGVDVRRELAESKL
KLALTIKDYPEKLKVAIALNGGLDETIQALMVSNSLHLGFDLSLNGGSNIELYLYIKKQEFQQI
HIQQRLAKVLSTQALQPLPACSRICVGLSKANAEKVVYYYLENFNDFLNYFAANDTARRVHA
YYREQPVKEMCVALQESELLAGTIQKMNLYYLI

Sequence in red is the TEV cleavage signal

N-terminal His$_6$ SUMO tag sequence is in blue.

AutF Expression of in *E. coli* and purification:

Full-length *autF* gene was amplified by PCR using Phusion^{Tm} High-Fedility DNA polymerase and the *g*DNA of cyanobacterial strain CCAP1446/10 as template. The gene was purified from agarose gel using QIAquick Gel Extraction Kit and cloned in pEHISTEV-SUMO plasmid (Gift from Dr Hunating Liu, University of St Andrews) in frame with an *N*-terminal Tobacco etch virus (TEV) protease-cleavable His$_6$SUMO tag using In-Fusion® cloning kit (Takara Bio) after linearisation of the vector by PCR (Figure S8A). Primers used in PCR reactions are listed in Table S3. The protein was expressed in *Escherichia coli* BL21 (DE3) cells. A seed culture was grown overnight on Luria-Bertani broth (LB) medium containing 50 µg/mL kanamycin and incubated at 37°C with shaking at 200 rpm and an aliquot (10 mL) was used to inoculate each litre of LB medium. Cultures were grown at 37°C with shaking at 200 rpm until OD_{600} is 0.4-0.5. Cultures were then cooled down to room temperature and IPTG was added to a final concentration of 0.5 mM. Induced cultures were further incubated at 30°C for 6 hours.

Cells were harvested by centrifugation at 4,000*g*, 4°C for 15 min and resuspended in lysis buffer (200 mM NaCl, 20 mM Tris (pH 8.0), 20 mM imidazole and 3 mM *β*-mercaptoethanol (BME) with the addition of complete EDTA-free protease inhibitor tablets (Thermo Scientific) and 0.4 mg g^{-1} DNase wet cells (Sigma). They were then lysed using STANSTED SPCH-EP-10 pressure cell homogeniser (Homogenising Systems Ltd, UK) and the lysate was cleared by centrifugation (40,000*g*, 4°C, 45 min) followed by filtration through a 0.45 µm membrane filter and loaded onto an Ni-Sepharose 6 FF column (Cytiva, Sweden) equilibrated in lysis buffer. The column was washed with 20 volumes of washing buffer containing 200 mM NaCl, 20 mM Tris (pH 8.0), 20 mM imidazole and 3 mM BME and AutF was eluted with 250 mM imidazole in the same buffer. The elution peak was loaded onto a HiPrep^{TM} 26/10 Desalting column (Cytiva, Sweden) equilibrated in 200 mM NaCl, 10 mM HEPES pH 8.0, 1 mM Tris(2-carboxyethyl) phosphine hydrochloride (TCEP) and 10% glycerol. The protein was concentrated to 150 µM using Vivaspin concentrators, 10 kDa molecular weight cut-off. Integrity and identity were confirmed by SDS-PAGE (Figure S8B) and MS. The purified protein was stored as flash-frozen aliquots at -80°C until used.

Site directed mutagenesis was carried out using In-Fusion® cloning kit (Takara Bio) following the manufacturer protocol and after PCR amplification using Phusion^{Tm} High-Fedility DNA polymerase and the primers listed in Table S3. The mutant protein was expressed and purified as above.

Solid phase peptide synthesis

The majority of the Fmoc amino acid derivatives, including Fmoc-D-Arg(Pbf)-OH, and the preloaded Fmoc-Asp(NovaSyn TGA)-OAll resin were purchased from Novabiochem, Merck Biosciences, UK. Fmoc-Leu-OH, Fmoc-Har(PbF)-OH, Fmoc-Gln(Trt)-OH, *N,N'*-diisopropylcarbodiimide (DIC), tetrakis(triphenylphosphine)palladium ($Pd(PPh_3)_4$), 1-[Bis(dimethylamino)methylene]-1H-1,2,3-triazolo[4,5-b]pyridinium 3-oxide hexafluorophosphate (HATU), trifluoroacetic acid (TFA), 7-Azabenzotriazol-1-yloxy)tripyrrolidino-phosphonium hexafluorophosphate (PyAOP) and triisopropylsilane (TIS) were acquired from Fluorochem, UK. Fmoc-L-Arg(Pbf)-OH, Fmoc-Tyr(tBu)-OH, Fmoc-Met-OH, ethyl cyano(hydroxyimino)acetate (Oxyma), Cl-TCP(Cl) ProTide resin and the Fmoc-Rink Amide ProTide (LL) resin was obtained from the CEM corporation, USA. *N*,*N*-dimethylformamide (DMF), dichloromethane (DCM), methanol (MeOH) and diethyl ether were purchased from VWR, Avantor, USA. The phenylsilane and the *N*,*N*-Diisopropylethylamine (DIPEA) were obtained from Alfa Aesar, UK. Piperidine was purchased from Merck Life Sciences, UK. Sodium diethyldithiocarbamate trihydrate, 2,2′-(ethylenedioxy)diethanethiol (DODT), potassium iodide (KI), pyridine and the Kaiser test kit were acquired from Sigma Aldrich.

Linear peptides and cyclic precursor peptides were prepared using the standard Fmoc-based solid-phase peptide synthesis (SPPS) strategy on a Liberty Blue™ Automated Microwave Peptide Synthesizer (CEM Corporation, USA) at a 0.1 mmol scale. A Fmoc-Rink Amide ProTide (LL) resin (0.19 mmol/g) was used for the synthesis of all these peptides. Initial deprotection of the Fmoc protecting group from the resin and all subsequent deprotections were performed with programed cycles using a solution of 20% piperidine in DMF. Coupling cycles in the synthesizer were performed, after deprotection, with solutions of 0.2 M Fmoc-amino acids, 1 M DIC and 1 M Oxyma in DMF. Most Fmoc amino acids underwent one coupling cycle during attachment. Fmoc-arginine residues underwent two coupling cycles before deprotection of the Fmoc. For all linear and cyclic precursor peptides, the *N*-terminal Fmoc group was cleaved at the end of the synthesis.

Cyclo[-LGPFRFD] (**7**) and *cyclo*[-LGPFrFD] (**6**) peptides were synthesized on a Fmoc-Asp(NovaSyn TGA)-OAll resin (0.17 mmol/g) under the same conditions as the other linear peptides with the exception of no final deprotection step being performed on the *N*-terminal leucine. The resin was then transferred to a fritted SPE column (Hicrom, Avantor UK) and treated with $Pd(PPh_3)_4$ (0.1 equiv.) and phenylsilane (20 equiv.) in DCM. The mixture was allowed to react for 40 min at RT on an orbital shaker (Heidolph, 1350 rpm) to remove the allyl ester protecting group from the *C*-terminal carboxylic acid.[S12] The resin was then washed with DMF, 5% sodium diethyldithiocarbamate trihydrate solution in DMF, 1% DIPEA in DMF and DMF again to remove any Pd remaining from the deprotection cocktail.[S12] Fmoc deprotection was performed manually on the resin using 20% piperidine in DMF for 30 min on a shaker (1350 rpm) at RT.[S12, S13] A Kaiser test was performed to confirm full deprotection. Cyclisation was performed manually on-resin by treatment with HATU (2 equiv.) and DIPEA (2 equiv.) in DMF for 19 hrs. on a shaker (1350 rpm) at RT.[S13] After cyclisation, a Kaiser test was performed to confirm cyclisation.

All peptides not containing methionine were deprotected and cleaved from the resins by treatment with a cleavage cocktail of TFA/TIS/H_2O (95:2.5:2.5) for 3 hrs. at RT on an orbital shaker (Heidolph, 1350 rpm). The cleavage mixture was concentrated by a stream of air. Peptides containing a methionine residue were treated with a cocktail solution of TFA/TIS/DODT/H_2O (92.5:2.5:2.5:2.5) and then concentrated under N_2 gas. The peptides were precipitated using cold diethyl ether, placed into a -20 freezer overnight, washed with ether (3x) and dried under vacuum to give a crude solid. The crude peptides were purified using reversed phase HPLC on an Agilent Technologies 1260 Infinity using a C18 column

(ACE 5 C18-HL, 5 μm, 10x250 mm, 100 Å) through an acetonitrile (+0.1% TFA)/Water (+0.1% TFA) gradient (see Table S4 for details). The collected fractions were subsequently lyophilized on a LaboGene CoolSafe Freeze dryer to give the pure solid. Identity and purity were confirmed by HPLC-MS analysis.

Cyclo[-TLrESTAMYp] (**2**) and cyclo[-TLRESTAMYp] (**3**) were synthesized on a Cl-TCP(Cl) ProTide resin (0.5 mmol/g) under the same conditions as conventional synthesis with the exception of a special coupling performed on the C-terminal alanine which was coupled to the resin using a double coupling cycle and utilizing a base of 1.0 M DIPEA and 0.125 M KI solution in DMF. 0.1 eq DIPEA was also added to the Oxyma activator solution. After synthesis the resin was transferred to a fritted SPE column and washed 6x with DCM (Placed on shaker for third and last wash) the resin was then treated with a cleavage solution of 1% TFA in DCM 5x for 2 min each on the shaker at RT. After each treatment, the solution was filtered into a solution of 10% pyridine in MeOH. All filtrates were combined and concentrated down under vacuum. Ice cold H_2O was added to the solution causing the protected peptide to precipitate out of solution. This solution was subsequently lyophilized to give a white powder. The identity was confirmed by LCMS. The protected peptides were cyclized in solution at a concentration of 2 mM with 3 eq of PyAOP and 5 eq of DIPEA at RT for two days using HPLC to monitor reaction completion. Once completed the reaction solution was concentrated down under vacuum and lyophilized.

The protection groups on the peptides were cleaved off using a cocktail solution of TFA/TIS/DODT/H_2O (92.5:2.5:2.5:2.5) for 3 hrs. at RT on an orbital shaker. The mixture was concentrated under a stream of N_2 and precipitated out using cold diethyl ether. The solid was washed, dried and purified under the same conditions as the linear peptides.

Enzymatic reactions

Macrocyclization reactions using the plant macrocyclase PCY1 [S14] were prepared for 250 µM peptide substrate and 1 µM purified enzyme in buffer containing 100 mM NaCl, 20 mM Tris HCl pH 8.5, 3 mM TCEP and 5% DMSO. Reaction mixtures were incubated at 30°C for 24 hrs. Macrocyclization reactions using the cyanobactin macrocyclase $PatG_{mac}$ [S15] contain 100 µM peptide, 5% DMSO, 500 mM NaCl, 20 µM $PatG_{mac}$, and 20 mM bicine pH 8.0. Those reactions were incubated at 37°C with shaking at 200 rpm for 4 days and monitored by MS.

Macrocyclization reaction mixtures were then subjected to solid phase extraction using Strata C18-E (55 µm, 70 Å; 2 g/12 mL, Giga tubes; Phenomenex) and the organic components were eluted with 100% methanol and concentrated under vacuum using rotary evaporator.

The eluate was then purified on the semipreparative HPLC C18 column (ACE 5 C18-HL) and the identity of the purified cyclic peptide products were confirmed by LCMS.

Prenylation reactions with AutF contain 100 µM peptide substrate, 20 µM purified enzyme, 12 mM $MgCl_2$, 1mM dimethylallyl pyrophosphate (DMAPP), 1% DMSO in buffer containing 150 mM NaCl, 10 mM HEPES pH 7.5 and 3 mM TCEP. Samples were analysed by LC-MS as described above (Figures S9-S32). Control samples were prepared by incubation of the peptide substrates in the aforementioned buffer. No prenylated peptides were detected in control samples, suggesting there was no spontaneous prenylation occurring independent of the enzyme.

Kinetics

The AutF steady-state kinetics parameters were assessed using 2 mM DMAPP and variable concentrations of the two substrates Fmoc-L-Homoarginine-OH (0.01 - 0.5 mM) and H-FRFDLGPAYD-NH_2 (0.05 – 1 mM) on the basis of product yield under different conditions as monitored by LCMS. The assays were conducted in duplicate and all rates were confirmed to be linear. The kinetics curves were fit to Michaelis-Menten kinetics (Figure S33) and were generated using prism 5.04 (GraphPad software, Inc. La Jolla, CA 92037 USA).

Amino acid analysis of autumnalamide B

Phormidium autumnale CCAP 1446/10 freeze-dried cells (0.7g) were extracted 2 times with 75 mL of methanol assisted by Silentcrusher homogenizer (20 kiloRPS for 1 min). After centrifugation at 7000 rcf for 7 min), supernatants were pooled to a rotavapor flask together with 10 mL C18 silica (Sepra™ C18-E (50 µm, 65 Å, Phenomenex) and the methanol was evaporated in a rotavapor. A Silica cartridge was primed with dichloromethane and ethyl acetate and the C18 silica was placed on top of a silica cartridge (Chromabond® SiOH (45 mL/ 5000 mg)). Cartridge was eluted with ethyl acetate until pigments were removed, then the compound of interest was eluted with methanol and methanol was evaporated away. Residue was dissolved in 10% methanol and final purification was done with Acquity Premier UPLC (PDA and QDA detectors, fraction manager, Waters). Fractions from multiple 10 µL injections to XSelect® HSS T3 column (4.6 x 150mm, 5 µm) eluted at 1 mL min^{-1} with a gradient starting with 95/5 0.1% HCOOH/(acetonitrile/isopropanol 50/50 + 0.1% HCOOH) changing linearly to 40/60 in 5 min, then to 0/100 in 0.1 min being there 1.9 min and then back to 95/5 in 0.1 mins, were pooled. Solvent was evaporated and solid autumnalamide B was used for chiral amino acid analysis by Marfey derivatization with 1-fluoro-2,4-dinitrophenyl-5-L-alanine.[S17] During the purification methionine was oxidised.

Marfey derivatives of the autumnalamide B acid hydrolysate and amino acid standards (from Sigma, methionine sulfone was from Fluka, 1 µL injections) were analyzed with UPLC UV/MS using Kinetex® C8 column (100 x 2.1 mm, 1.7 µm, 100Å) eluted 0.3 ml min-1 at 40 °C with 0.1%HCOOH (solvent A) and acetonitrile + 0.1% HCOOH (solvent B). Gradient started from 95/5 (A/B) going to 40/60 in ten mins, then to 0/100 in 0.1 mins and kept there 3.9 mins, then back to 95/5 in 0.1 mins with total run time of 20 min. Compounds were detected with mass detector using full scan negative ionization.

As arginine Marfey derivatives did not resolve with reversed phase C8 column, a HILIC method was used. One µL samples were injected to Acquity UPLC® BEH Amide column (2.1 x 100mm, 1.7 µm) eluted 0.3 mL.min^{-1} at 40 °C with 0.2% ammonium formate (solvent A) and acetonitrile (solvent B). Gradient started from 90/10 (A/B) going to 40/60 in nine mins, kept there 1 min, then back to 90/10 in 0.1 mins with total run time of 16 min. Results are shown in Figure S5.

References

[S1] A. M. Bolger, M. Lohse and B. Usadel, *Bioinformatics,* 2014, **30**, 2114–2120.

[S2] A. Bankevich, S. Nurk, D. Antipov, A. A. Gurevich, M. Dvorkin, et al., *J. Comput. Biol.,* 2012, **19**, 455-477.

[S3] D. E. Wood and S. L. Salzberg, *Genome Biol.,* 2014, **15**, R46.

[S4] D. O. Alvarenga, M. F. Fiore and A. M. Varani, *Front. Microbiol.,* 2017, **8**, article 809.

[S5] R. Kajitani, K. Toshimoto, H. Noguchi, A. Toyoda, Y. Ogura, M. Okuno, M. Yabana, M. Harada, E. Nagayasu, H. Maruyama, Y. Kohara, A. Fujiyama, T. Hayashi and T. Itoh, *Genome Res.* 2014, **24**, 1384–1395.

[S6] K. Tamura, G. Stecher and S. Kumar, *Mol. Biol. Evol.,* 2021, **38**, 3022–3027.

[S7] D. Darriba, G. L. Taboada, R. Doallo and D. Posada, *Nat. Methods,* 2012, **9**, 772–772.

[S8] F. Ronquist, M. Teslenko, P. van der Mark, D. L. Ayres, A. Darling, S. Höhna, B. Larget, L. Liu, M. A. Suchard and J. P. Huelsenbeck, *Syst. Biol.,* 2012, **61**, 539–542.

[S9] C. Camacho, G. Coulouris, V. Avagyan, N. Ma, J. Papadopoulos, K. Bealer and T. L. Madden, *BMC Bioinformatics,* 2009, **10**, 421.

[S10] L. Dalponte, A. Parajuli, E. Younger, A. Mattila, J. Jokela, M. Wahlsten, N. Leikoski, K. Sivonen, S. A. Jarmusch, W. E. Houssen and D. P. Fewer, *Biochemistry,* 2018, **57**, 6860−6867.

[S11] K. Rutherford, J. Parkhill, J. Crook, T. Horsnell, P. Rice, M. A. Rajandream and B. Barrell, *Bioinforma. Oxf. Engl.* 2000, **16**, 944–945.

[S12] R. A. Davis, K. Lau, S. H. Hausner and J. L. Sutcliffe, *Org. Biomol. Chem.* 2016, **14**, 8659-8663.

[S13] C. F. McCusker, P. J. Kocienski, F. T. Boyle and A. G. Schätzlein, *Bioorg. Med. Chem. Lett.* 2002, **12**, 547–549.

[S14] H. Ludewig, C. M. Czekster, E. Oueis, E. S. Munday, M. Arshad, S. A. Synowsky, A. F. Bent and J. H. Naismith, *ACS Chem Biol.* 2018, **13**, 801-811.

[S15] J. Koehnke, A. Bent, W. E. Houssen, D. Zollman, F. Morawitz, S. Shirran, J. Vendome, A. F. Nneoyiegbe, L. Trembleau, C. H. Botting, M. C. M. Smith, M. Jaspars and J. H. Naismith, *Nat. Struct. Mol. Biol.* 2012, **19**, 767 – 772.

[S16] A. Waterhouse, M. Bertoni, S. Bienert, G. Studer, G. Tauriello, et al., *Nucleic Acids Res.,* 2018, **46**, W296 – W303.

[S17] L. M. P. Heinilä, J. Jokela, M. N. Ahmed, M. Walsten, K. Saurav, P. Hrouzek, P. Permi, H. Koistinen, D. P. Fewer, K. Sivonen, *Org. Biomol. Chem.*, 2022, **20**, 2681–2692.

Supporting Tables

Table S1. Detection of autumnalamide A and B and other variants by LC-HRMS showing exact masses for both single and double protonated ions.

**Autumnalmide variant sequence**	**Molecular formula M**	**t_R (min)**	**$[M+nH]^{n+}$**	**Theoretical (*m/z*)**	**Observed (*m/z*)**	**Error (ppm)**
**Autumnalmide B:** *Cyclo*[-TLRESTAMYP]	$C_{50}H_{79}N_{13}O_{16}S$	3.50	1	1150.55612	1150.5566	0.4
			2	575.78170	575.7811	-1.0
Cyclo[-TLR(prenyl)ESTAMYP]	$C_{55}H_{87}N_{13}O_{16}S$	4.78	1	1218.61872	1218.6197	0.8
			2	609.81300	609.8113	-2.8
Cyclo[-GLDFrFp]	$C_{41}H_{56}N_{10}O_9$	4.96	1	833.43045	833.4299	-0.7
			2	417.21886	417.2177	-2.8
**Autumnalamide A:** *Cyclo*[-GLDFr(prenyl)Fp]	$C_{46}H_{64}N_{10}O_9$	6.44	1	901.49305	901.4929	-0.2
			2	451.25016	451.2493	-1.9

Table S2. Annotation of the autumnalamide (*aut*) biosynthetic gene cluster from *Phormidium autumnale* CCAP1446/1

aut **biosynthetic gene cluster**				**BLASTp results**			
**Protein**	**Length (aa)**	**Predicted function**	**Locus_tag**	**Description**	**Identity (%)**	**Organism**	**Accession**
AutA	674	*N*-terminal protease	K4039_073 30	PatA/PatG family cyanobactin maturation protease	79.53	*Oscillatoriales* cyanobacterium	TAG93315
AutB	75	Unknown	K4039_073 25	cyanobactin biosynthesis system PatB/AcyB/McaB family protein	92.96	*Oscillatoriales* cyanobacterium	TAG93316
AutC	503	Unknown	K4039_073 20	cyanobactin biosynthesis PatC/TenC/TruC family protein	88.47	*Tychonema* sp. LEGE 06208	WP_19406 6075
AutE2	49	Precursor peptide	K4039_073 10	Anacyclamide/piricycla mide family prenylated cyclic peptide	53.06	*Planktothrix rubescens* NIVA-CYA 18	CAC53451 60
AutE1	47	Precursor peptide	K4039_073 05	Precursor peptide	50.00	*Planktothrix agardhii* NIES-596	AED99433
AutF	289	Prenyltransfera se	K4039_073 00	LynF/TruF/PatF family peptide *O*-prenyltransferase	75.09	*Dolichospermum circinale* AWQC310F	WP_03536 7793
AutG	718	*C*-terminal protease	K4039_072 95	Peptidase S8	67.79	Cyanobacteria bacterium UBA11372	HAX79128

Table S3. Primers used for cloning and site directed mutagenesis (SDM)

**Experiment**	**Name**	**Sequence**
Cloning of autF in pEHISTEV-SUMO		
Linearisation of pEHISTEV-SUMO vector	Forward 5pTevSUMO<br>Reverse 3pTevSUMO	5'- ACTCGAGCACCACCA -3'<br>5'- GCCCTGAAAATACAG -3'
Amplification of *autF* gene	Forward 5autF_SUMO<br>Reverse 3autF_SUMO	5'- GTATTTTCAGGGCACAACCTTGTTAG -3'<br>5'- TGGTGCTCGAGTTTAAATCAGGTAGTAC -3'
SDM to prepare AutFA133G varianat		
	Forward 5Gly<br>Reverse 3Gly	5'- GTTAAAGCTGGGCCTAACG -3'<br>5'- CTTTAATCGTTAGGCCCAGC -3'

Table S4: Acetonitrile (AcN) (+0.1% TFA) and H_2O (+0.1% TFA) gradient purification methods for each synthesised peptide

Peptide	HPLC Acetonitrile/H_2O Purification Method	Retention time (min)
Cyclo[-TLRESTAMYP] **(1)**	15% - 40% ACN(+0.1%TFA) in H_2O (+0.1% TFA) in 15 min 4.7 mL/min	10.8
Cyclo[-TLrESTAMYp] **(2)**	15% - 30% ACN(+0.1%TFA) in H2O (+0.1% TFA) in 20 min	15.9
Cyclo[-TLRESTAMYp] **(3)**	15% - 50% ACN(+0.1%TFA) in H2O (+0.1% TFA) in 15 min	9.0
Cyclo[-TLrESTAMYP] **(4)**	15% - 40% ACN(+0.1%TFA) in H_2O (+0.1% TFA) in 15 min 4.7 mL/min	10.5
Cyclo[-LGPFrFD] **(6)**	25% - 50% ACN(+0.1%TFA) in H_2O (+0.1% TFA) in 15 min 4.7 mL/min	11.9
Cyclo[-LGPFRFD] **(7)**	25% - 50% ACN(+0.1%TFA) in H_2O (+0.1% TFA) in 20 min 4.7 mL/min	11.5
H-FrFDLGpAYD-NH_2 **(8)**	20% - 40% ACN(+0.1%TFA) in H_2O (+0.1% TFA) in 15 min 4.7 mL/min	8.5
H-FrFDLGPAYD-NH_2 **(9)**	15% - 40% ACN(+0.1%TFA) in H_2O (+0.1% TFA) in 15 min 4.7 mL/min	11.0
H-FRFDLGPAYD-NH_2 **(10)**	25% - 40% ACN(+0.1%TFA) in H_2O (+0.1% TFA) in 15 min 4.7 mL/min	7.1
H-RFDLGPF-NH_2 **(11)**	20% - 50% ACN(+0.1%TFA) in H_2O (+0.1% TFA) in 15 min 4.7 mL/min	10.1
H-rFDLGPF-NH_2 **(12)**	20% - 50% ACN(+0.1%TFA) in H_2O (+0.1% TFA) in 15 min 4.7 mL/min	9.4
H-FDLGPFR-NH_2 **(13)**	20% - 40% ACN(+0.1%TFA) in H_2O (+0.1% TFA) in 15 min 4.7 mL/min	10.0
H-FDLGPFr-NH_2 **(14)**	20% - 40% ACN(+0.1%TFA) in H_2O (+0.1% TFA) in 15 min 4.7 mL/min	10.2
H-FREDLGPAYD-NH_2 **(15)**	10% - 35% ACN(+0.1%TFA) in H2O (+0.1% TFA) in 15 min 4.7 mL/min	11.3
H-FRADLGPAYD-NH_2 **(16)**	15% - 40% ACN(+0.1%TFA) in H2O (+0.1% TFA) in 15 min 4.7 mL/min	9.5
H-FRXDLGPAYD-NH_2 **(22)** X= L-homoarginine	15% - 50% ACN(+0.1%TFA) in H_2O (+0.1% TFA) in 15 min 4.7 mL/min	10.4
H-QYLDEKLPNG-NH_2 **(23)**	15% - 40% ACN(+0.1%TFA) in H2O (+0.1% TFA) in 15 min 4.7 mL/min	7.5
H-VVKGALKSLV-NH_2 **(24)**	15% - 40% ACN(+0.1%TFA) in H2O (+0.1% TFA) in 15 min 4.7 mL/min	9.7

Table S5: ^{13}C NMR (100 MHz) and ^{1}H (400 MHz) NMR data for *N*$^{\omega}$-prenyl homoarginine in DMSO-*d*$_6$. ^{13}C values from carbon and HSQC spectra and ^{1}H values from proton and HSQC spectra.

No	^{13}C, mult.	^{1}H	mult., *J* (Hz)	HMBC
1, 1'	140.7, C	-		
2, 2'	143.8, C	-		
3	46.7, CH	4.22		1, 1', 2, 2', 4, 4', 5, 5', 8
4, 4 '	125.3, CH	7.72		1, 1', 3, 6, 6', 7, 7'
5, 5'	127.1, CH	7.33		2, 2', 3, 4, 4', 7, 7'
6, 6'	127.6, CH	7.42		1, 1', 3, 4, 4', 7, 7'
7, 7'	120.1, CH	7.89	d, 7.5	1, 1', 2, 2', 4, 4', 5, 5'
8	65.6, CH_2	4.28		2, 2', 3, 9
9	156.2, C	-		
10	173.9, C	-		
11	53.7, CH	3.93		9, 10, 12, 13
11-NH	-	7.63	d, 8.3	9, 10, 11, 12
12	30.4, CH_2	1.62 1.71		10, 11, 13
13	22.8, CH_2	1.35		11, 12, 14, 15
14	28.0, CH_2	1.47		12, 13, 15
15	40.7, CH_2	3.11		13, 14, 16
15-NH	-	7.49		14, 15, 16
16	155.5, C	-		
16-C=NH	-			
16-NH	-	7.50		16, 17, 18
17	39.0, CH_2	3.72		16, 18, 19, 20, 21
18	119.2, CH	5.17		17, 20, 21
19	136.0, C	-		
20	17.8, CH_3	1.63	s	18, 19, 21
21	25.3, CH_3	1.69	s	18, 19, 20

Supporting Figures

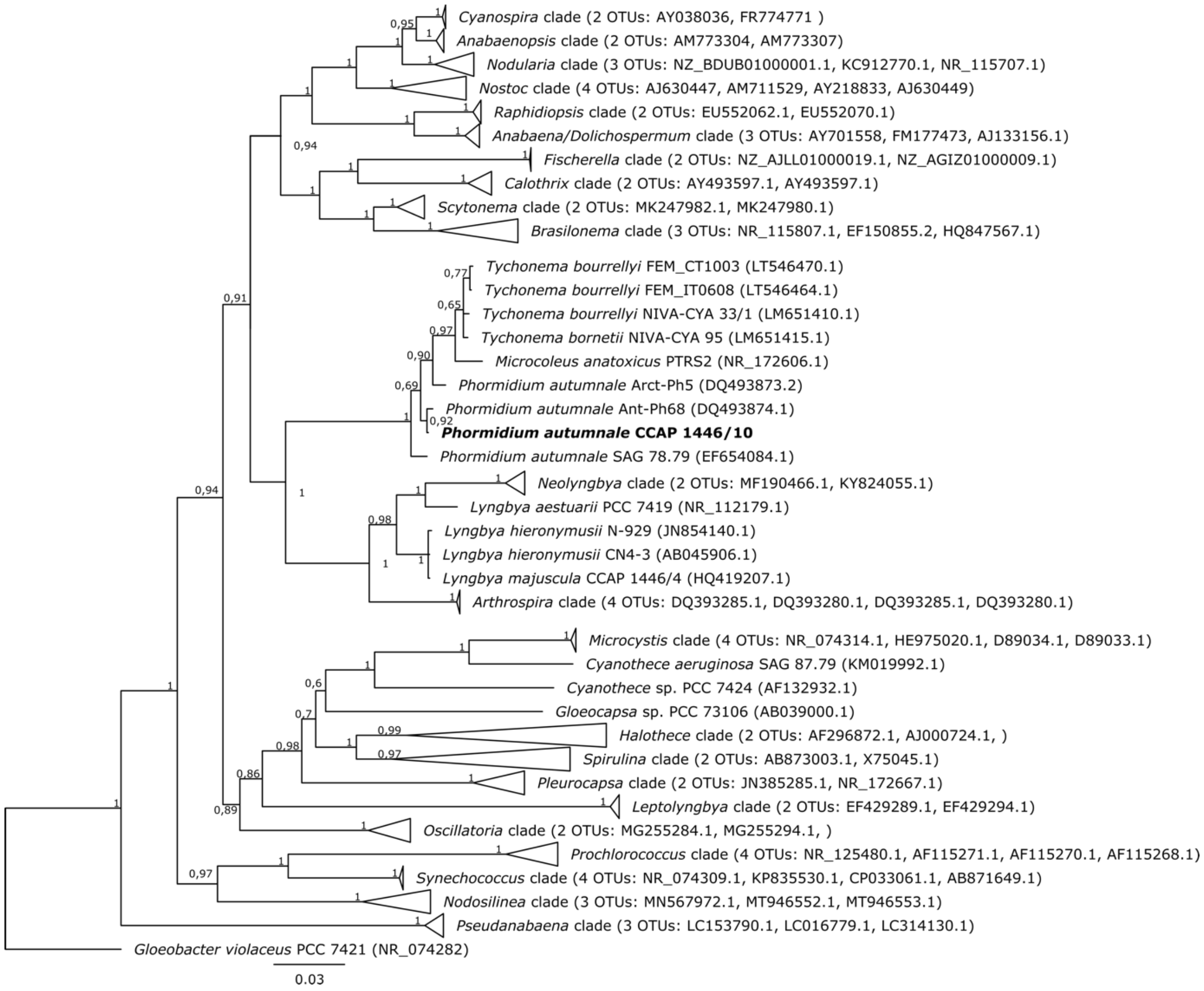

Figure S1: Bayesian inference tree based on the 16S rRNA genes from 76 cyanobacterial strains constructed with 5,000,000 generations. Accession number of the sequences is shown in parentheses and node labels represent the posterior probability values. The strain *Phormidium autumnale* CCAP1446/10 is shown in bold.

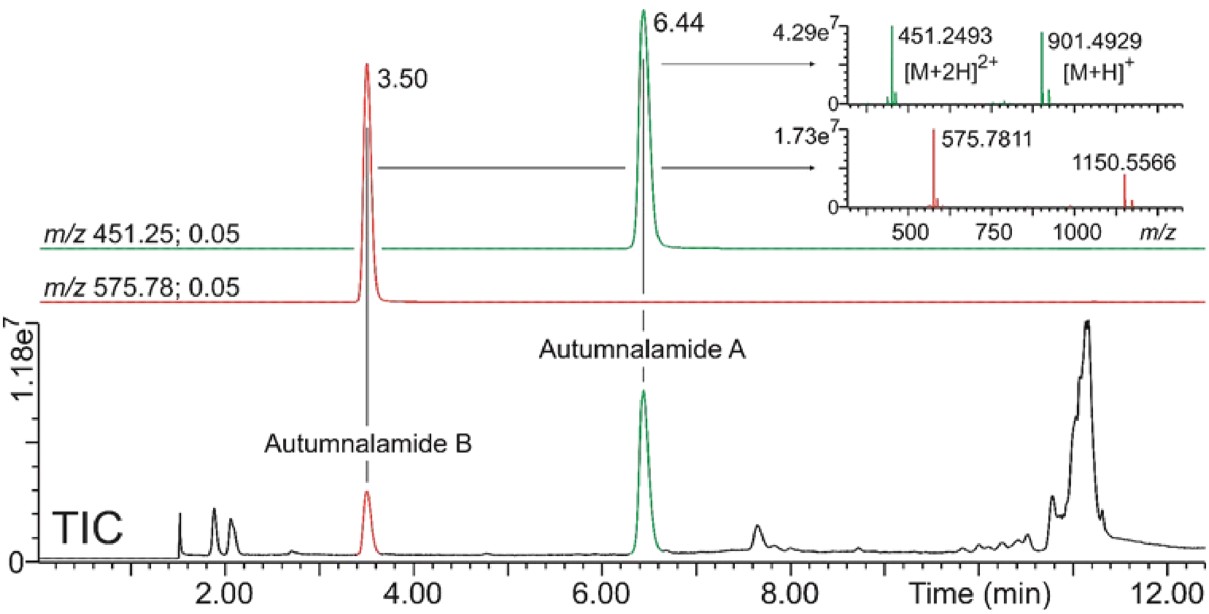

Figure S2: Total ion chromatogram (TIC) from *Phormidium autumnale* CCAP1446/10 extract, autumnalamide A and B specific (*m/z* 451.25 and *m/z* 575.78 $[M+2H]^{2+}$) extracted ion chromatograms showing the retention times of autumnalamide A and B. Single and double protonated molecule ions of autumnalamide A and B shown in right top corner.

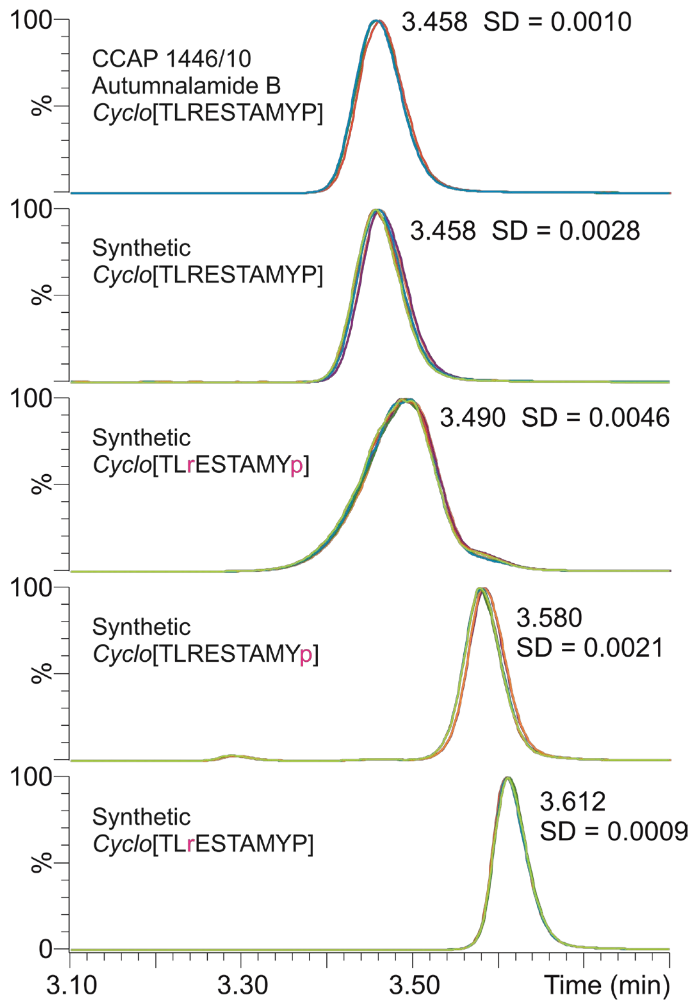

Figure S3: Selected Ion (*m/z* 575.78; $[M+2H]^{2+}$) Recording (SIR) chromatograms of autumnalamide B from *Phormidium autumnale* CCAP1446/10 extract and synthetic autumnalamide B stereochemical variants **1-4**. Each synthetic variant was injected six times and CCAP1446/10 extract three times.

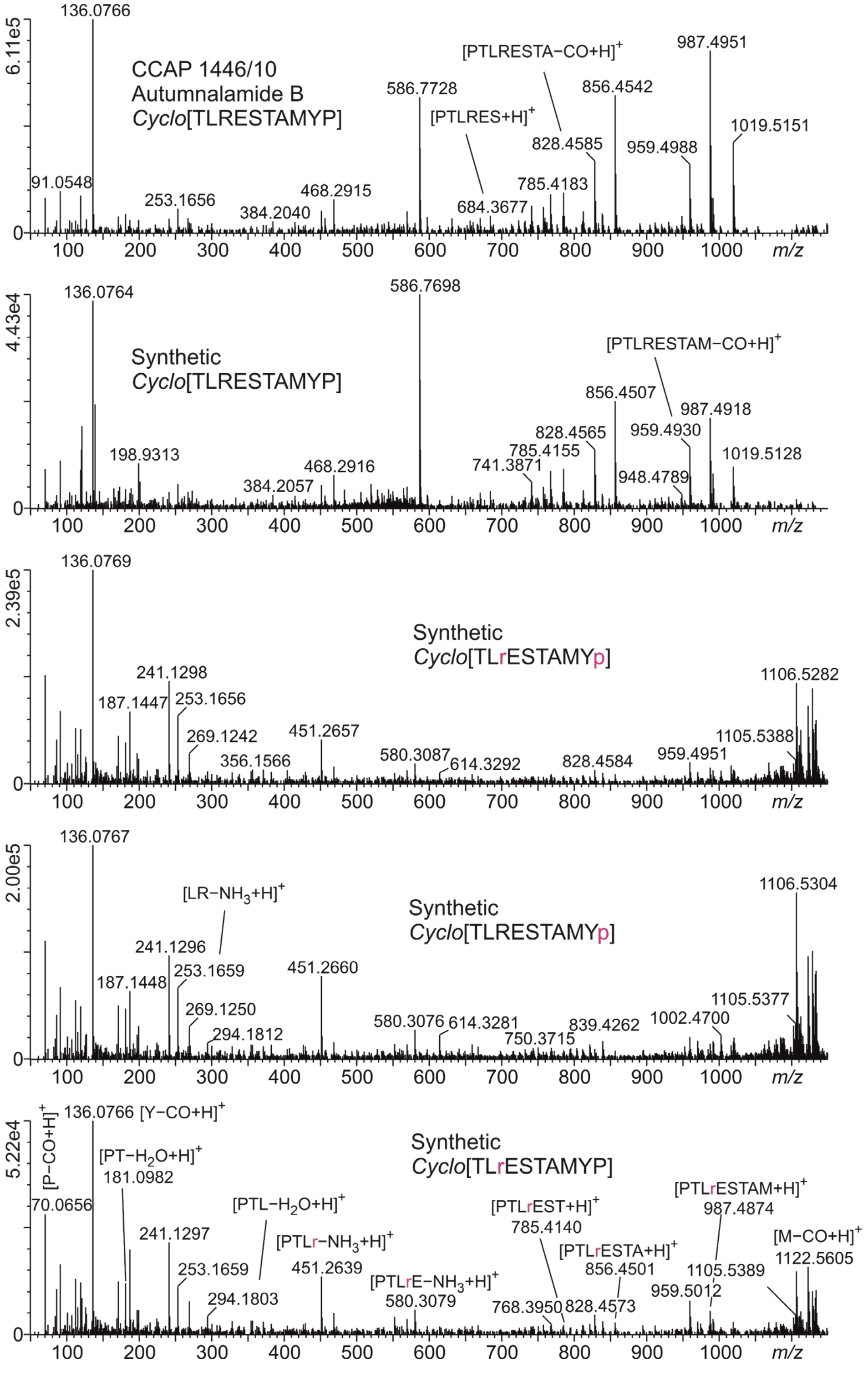

Figure S4: MS^E (E: elevated collision energy) spectra of the autumnalamide B (*m/z* 575.78; $[M+2H]^{2+}$) chromatogram peaks of the *Phormidium autumnale* CCAP1446/10 extract and four synthetic autumnalamide B stereochemical variants (1-4).

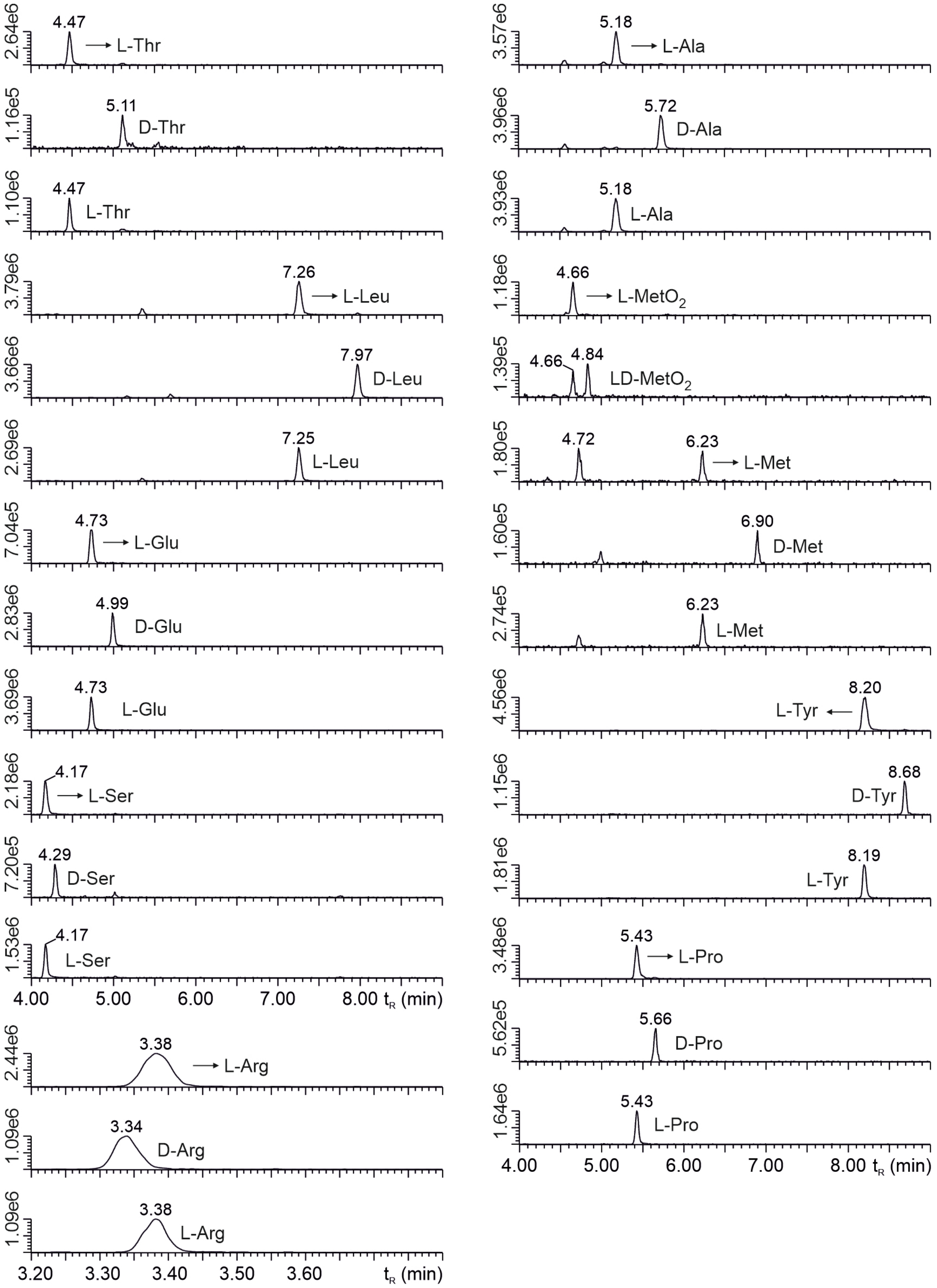

Figure S5: Extracted ion chromatograms (EICs) of Marfey derivative (1-fluoro-2,4-dinitrophenyl-5-L-alanine) L- and D-amino acids. Amino acid pointed with arrow originate from the autumnalamide B hydrolysate. EICs negative ion *m/z* values: Ala 340, Ser 356, Pro 366, Thr 370, Leu 382, Glu 398, Met 400, MetO2 432, Arg 425, Tyr 684 (double derivative).

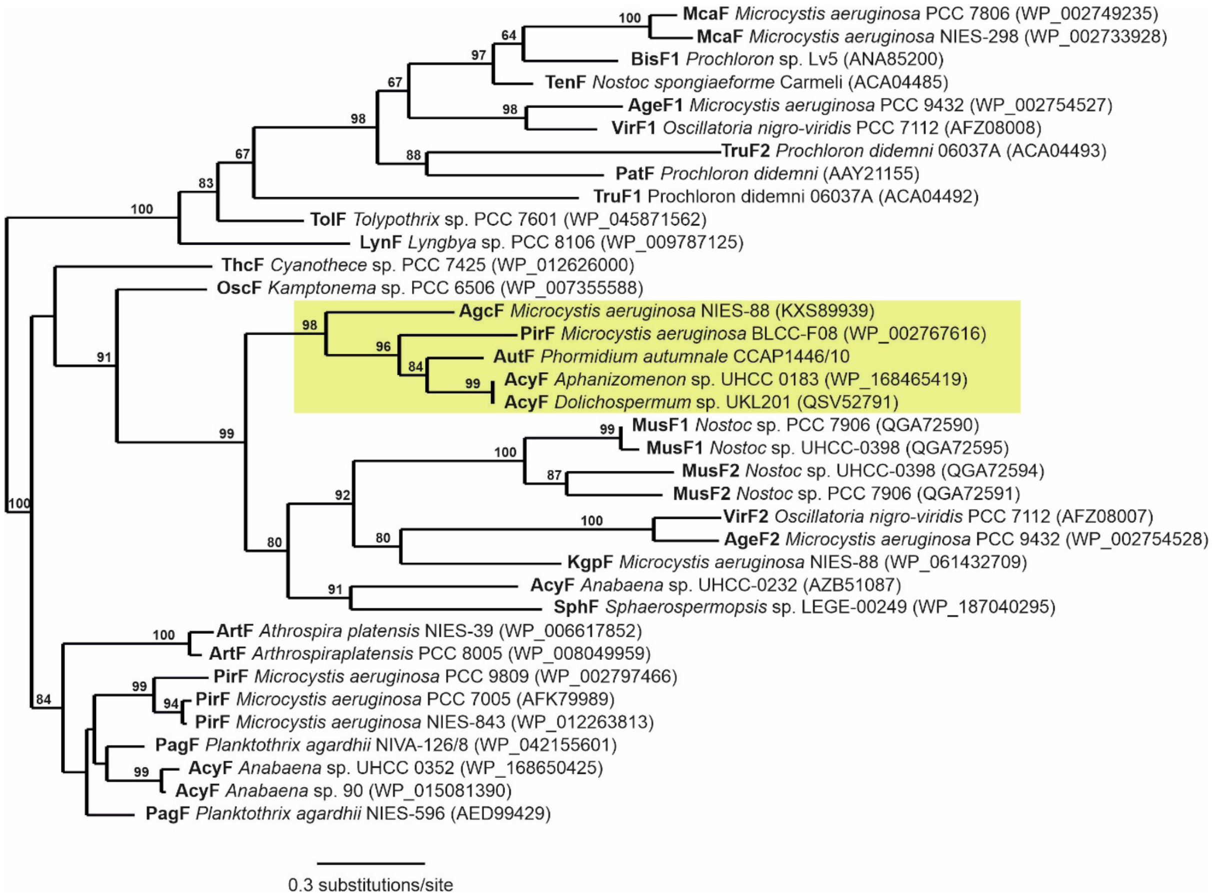

Figure S6: A PhyML phylogenetic tree showing the relationship between AutF and AgcF prenyltransferases. Phylogeny of 36 prenyltransferases from a variety of cyanobactin biosynthetic pathways showing the position of the AutF and AgcF enzymes. Bootstrap values are indicated at the node.

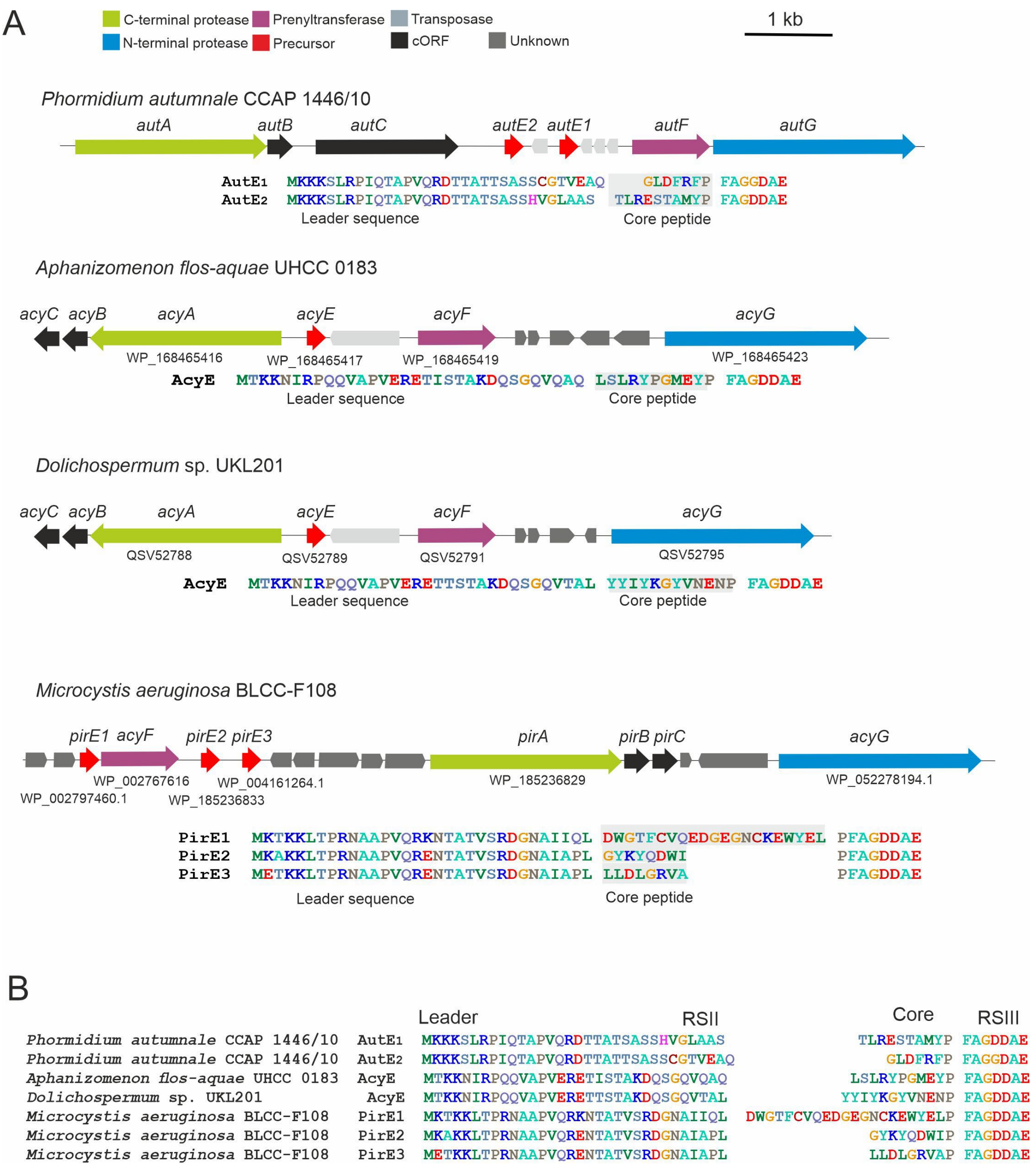

Figure S7: Bioinformatic analysis of the autumnalamide biosynthetic pathway. (A) Cyanobactin biosynthetic gene clusters from the autumnalamide (*aut*), anacyclamide (*acy*) and piricyclamide (*pir*) biosynthetic gene clusters that encode AutF and AgcF prenyltransferases homologs. (B) Precursor proteins encoded in these four biosynthetic pathways showing the leader and core sequences as well as the presence of recognition sequences (RS), RSII and RSIII.

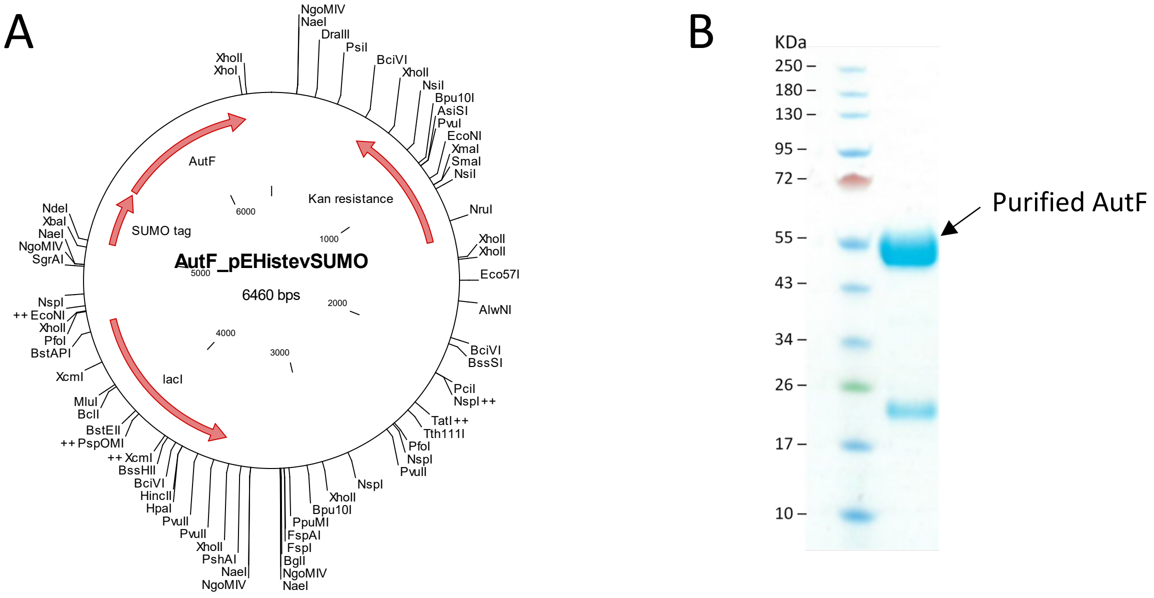

Figure S8: (A) Map of the construct containing *autF* gene in pEHistevSUMO vector. (B) SDS-PAGE gel shows the purified AutF protein.

A

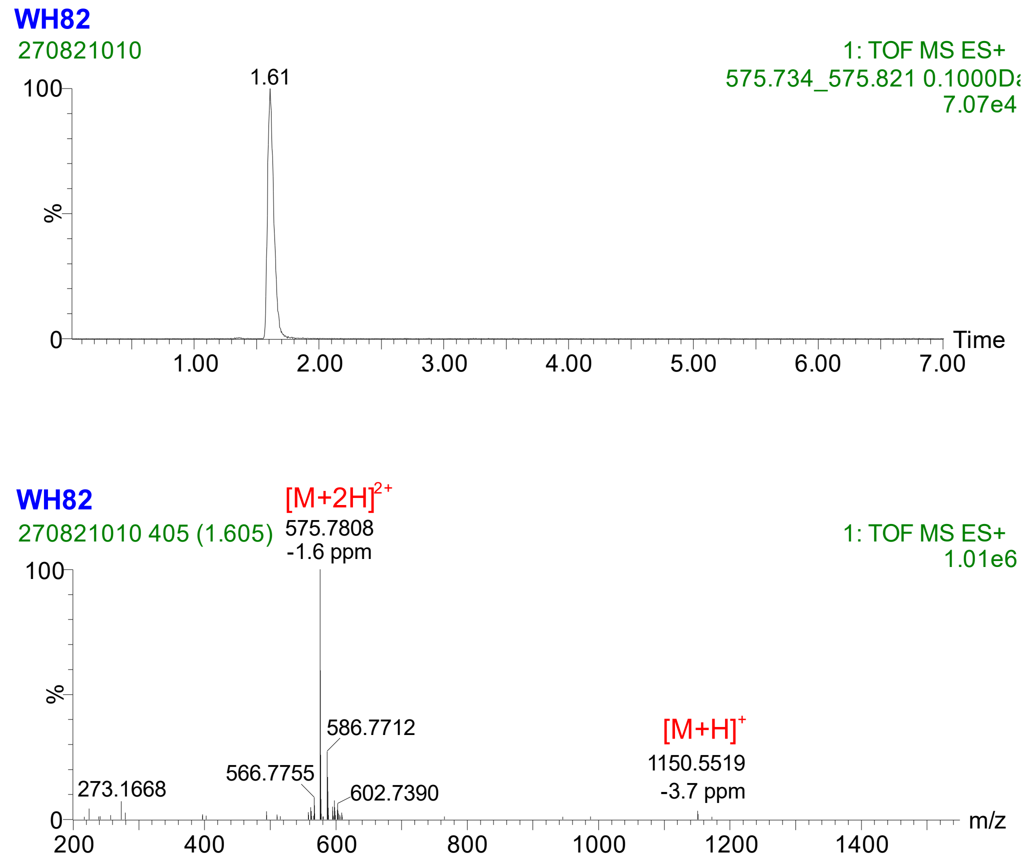

WH82
270821010
1: TOF MS ES+
575.734_575.821 0.1000Da
7.07e4
1.61
100
%
0
Time
1.00
2.00
3.00
4.00
5.00
6.00
7.00
WH82
270821010 405 (1.605)
[M+2H]2+
575.7808
-1.6 ppm
1: TOF MS ES+
1.01e6
586.7712
566.7755
273.1668
602.7390
[M+H]+
1150.5519
-3.7 ppm
m/z
200
400
600
800
1000
1200
1400

B

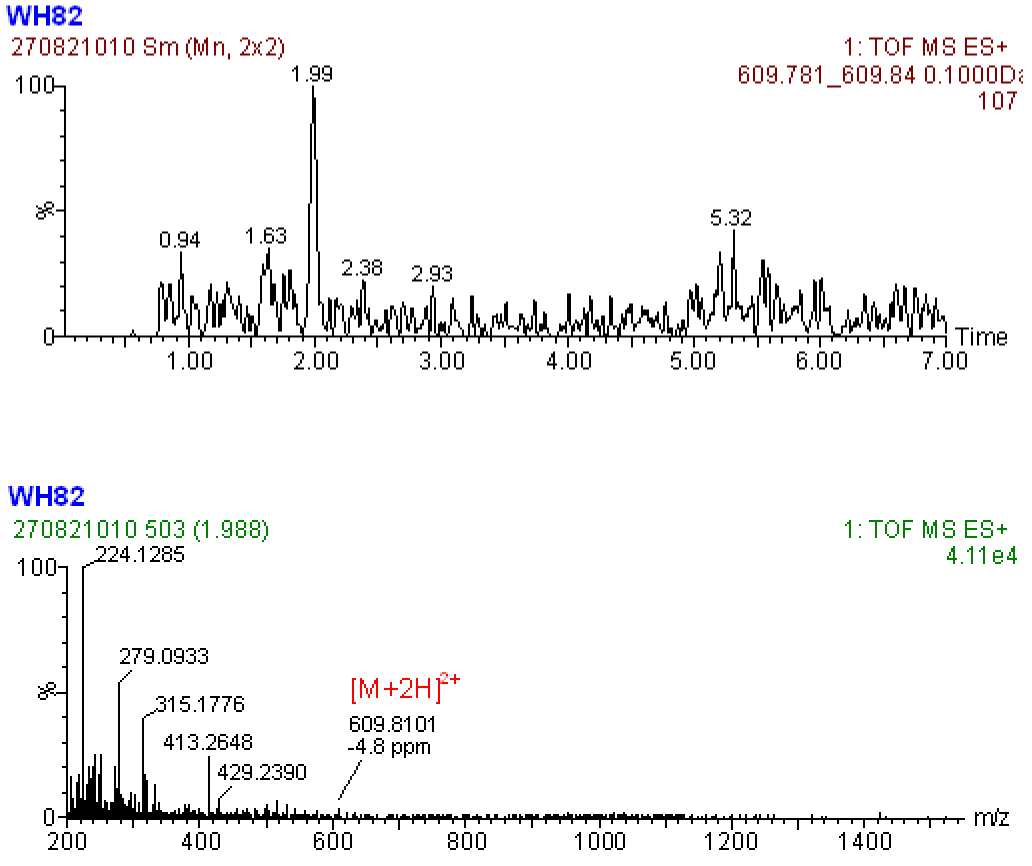

WH82
270821010 Sm (Mn, 2x2)
1: TOF MS ES+
609.781_609.84 0.1000Da
107
1.99
0.94
1.63
2.38
2.93
5.32
100
%
0
Time
1.00
2.00
3.00
4.00
5.00
6.00
7.00
WH82
270821010 503 (1.988)
1: TOF MS ES+
4.11e4
224.1285
279.0933
315.1776
413.2648
429.2390
[M+2H]2+
609.8101
-4.8 ppm
m/z
200
400
600
800
1000
1200
1400

Figure S9: LC HRMS of the biochemical reaction of peptide *Cyclo* [-TLRESTAMYP] (**1**) and AutF in the presence of DMAPP. A) LCMS of the unprocessed substrate, B) LCMS of the prenylated product.

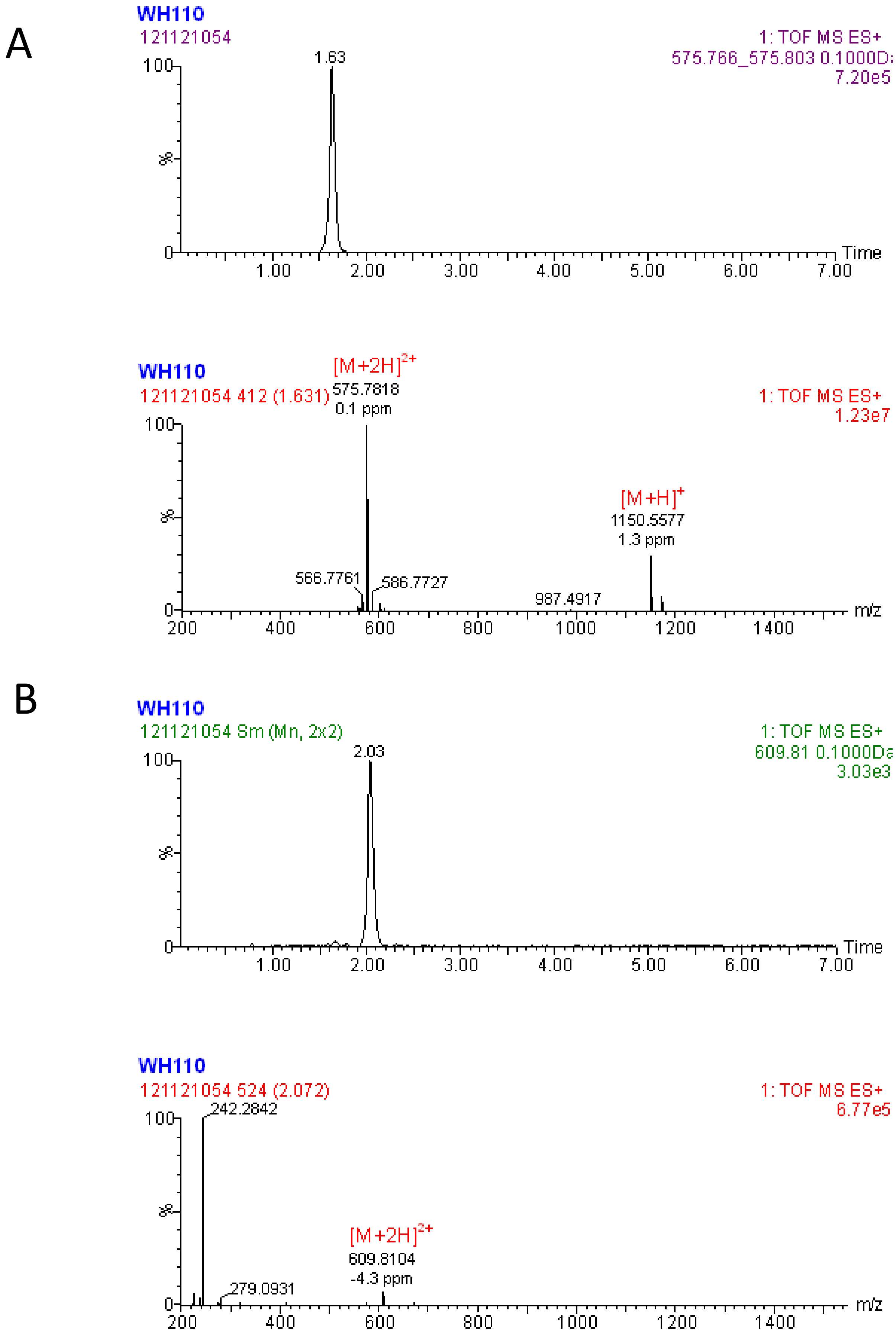
A
WH110
121121054
1: TOF MS ES+
575.766_575.803 0.1000Da
7.20e5
1.63
100
%
0
Time
1.00
2.00
3.00
4.00
5.00
6.00
7.00
WH110
121121054 412 (1.631)
[M+2H]2+
575.7818
0.1 ppm
1: TOF MS ES+
1.23e7
[M+H]+
1150.5577
1.3 ppm
566.7761
586.7727
987.4917
m/z
200
400
600
800
1000
1200
1400
B
WH110
121121054 Sm (Mn, 2x2)
1: TOF MS ES+
609.81 0.1000Da
3.03e3
2.03
Time
WH110
121121054 524 (2.072)
1: TOF MS ES+
6.77e5
242.2842
279.0931
[M+2H]2+
609.8104
-4.3 ppm
m/z

Figure S10: LC HRMS of the biochemical reaction of peptide *Cyclo* [-TLrESTAMYp] (**2**) and AutF in the presence of DMAPP. A) LCMS of the unprocessed substrate, B) LCMS of the prenylated product.

A

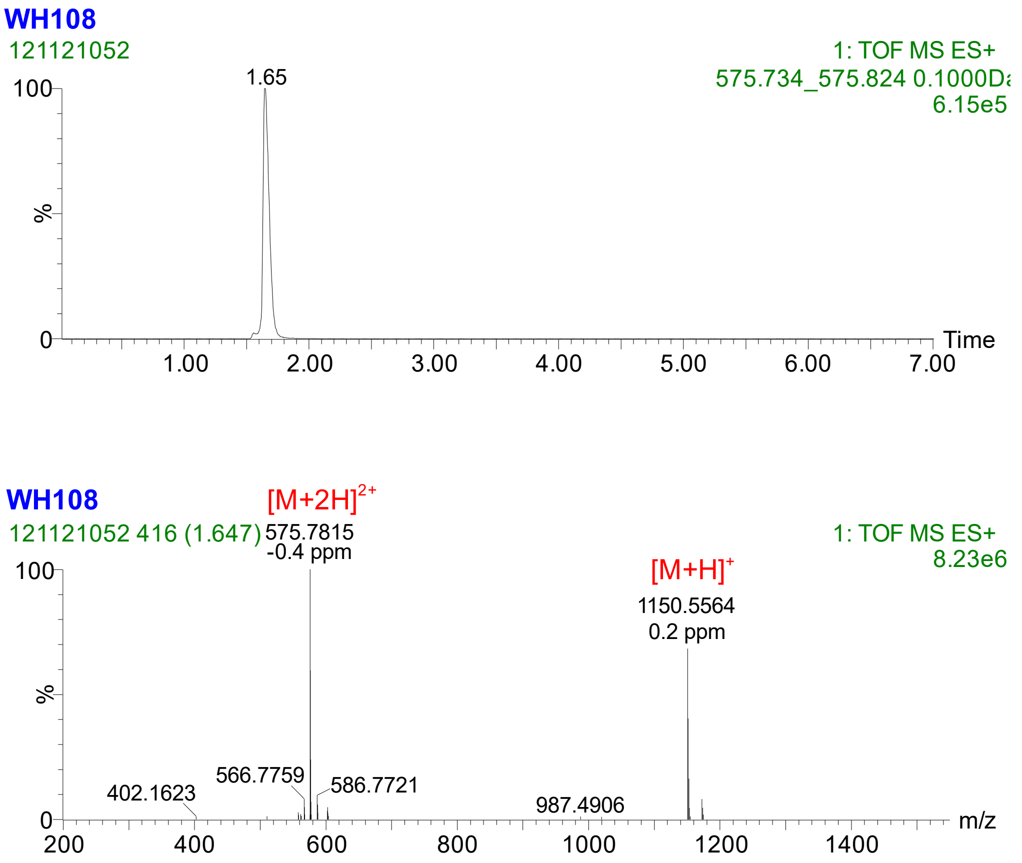
WH108
121121052
1: TOF MS ES+
575.734_575.824 0.1000Da
6.15e5
1.65
100
%
0
1.00
2.00
3.00
4.00
5.00
6.00
7.00
Time
WH108
121121052 416 (1.647)
[M+2H]$^{2+}$
575.7815
-0.4 ppm
1: TOF MS ES+
8.23e6
[M+H]$^{+}$
1150.5564
0.2 ppm
402.1623
566.7759
586.7721
987.4906
200
400
600
800
1000
1200
1400
m/z

B

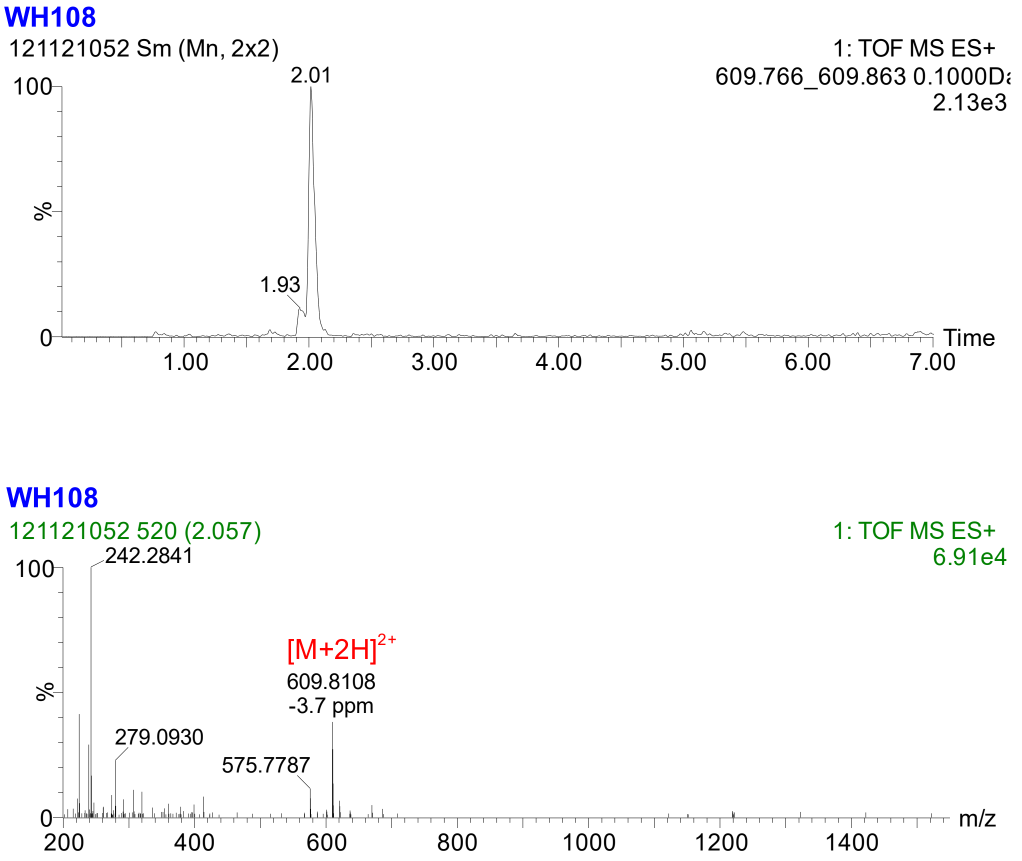
WH108
121121052 Sm (Mn, 2x2)
1: TOF MS ES+
609.766_609.863 0.1000Da
2.13e3
2.01
1.93
100
%
0
1.00
2.00
3.00
4.00
5.00
6.00
7.00
Time
WH108
121121052 520 (2.057)
1: TOF MS ES+
6.91e4
242.2841
[M+2H]$^{2+}$
609.8108
-3.7 ppm
279.0930
575.7787
200
400
600
800
1000
1200
1400
m/z

Figure S11: LC HRMS of the biochemical reaction of peptide *Cyclo* [-TLRESTAMYp] (**3**) and AutF in the presence of DMAPP. A) LCMS of the unprocessed substrate, B) LCMS of the prenylated product.

A

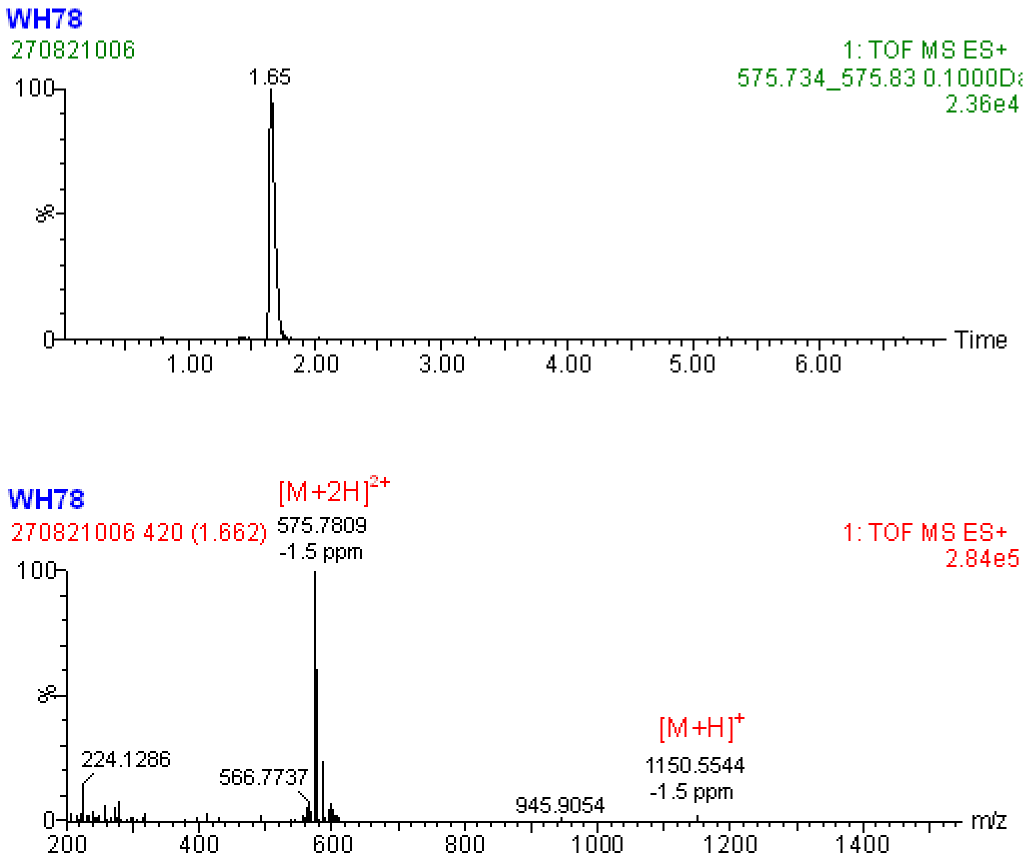
WH78
270821006
1: TOF MS ES+
575.734_575.83 0.1000Da
2.36e4
1.65
100
%
0
1.00
2.00
3.00
4.00
5.00
6.00
Time
WH78
270821006 420 (1.662)
[M+2H]$^{2+}$
575.7809
-1.5 ppm
1: TOF MS ES+
2.84e5
224.1286
566.7737
945.9054
[M+H]$^{+}$
1150.5544
-1.5 ppm
m/z
200
400
600
800
1000
1200
1400

B

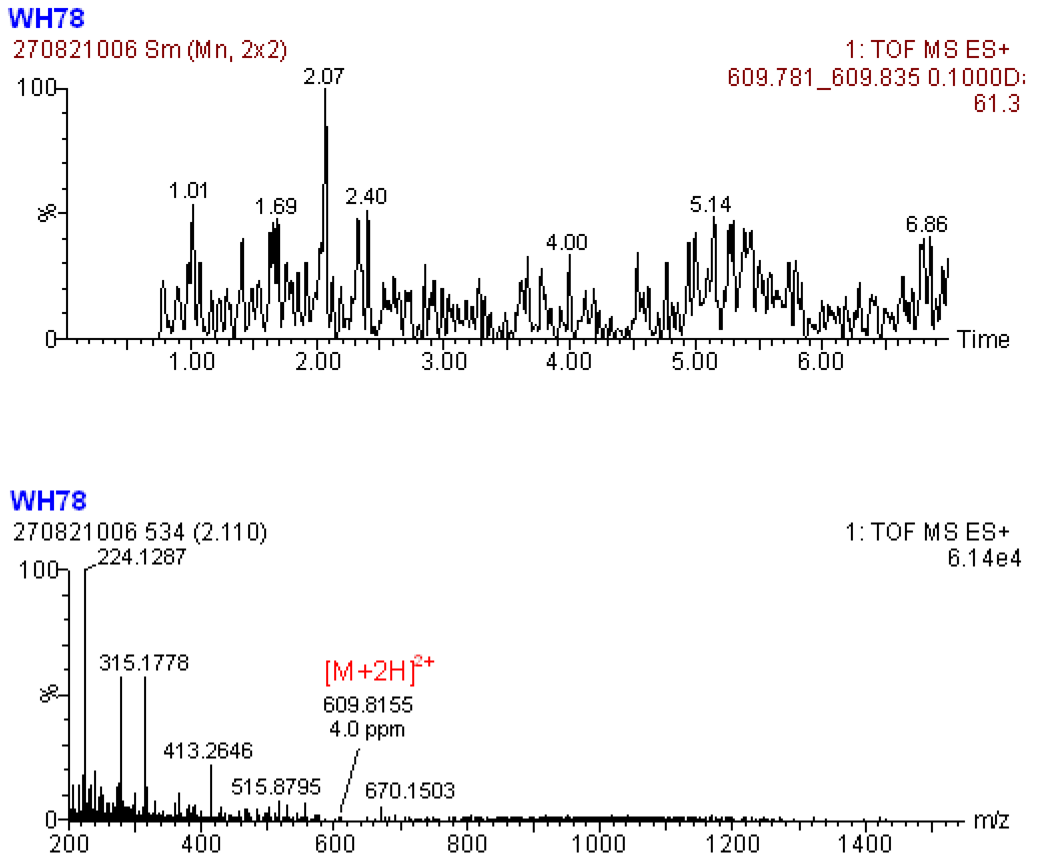
WH78
270821006 Sm (Mn, 2x2)
1: TOF MS ES+
609.781_609.835 0.1000Da
61.3
2.07
1.01
1.69
2.40
4.00
5.14
6.86
Time
WH78
270821006 534 (2.110)
1: TOF MS ES+
6.14e4
224.1287
315.1778
[M+2H]$^{2+}$
609.8155
4.0 ppm
413.2646
515.8795
670.1503
m/z
200
400
600
800
1000
1200
1400

Figure S12: LC HRMS of the biochemical reaction of peptide *Cyclo* [-TLrESTAMYP] (**4**) and AutF in the presence of DMAPP. A) LCMS of the unprocessed substrate, B) LCMS of the prenylated product.

A

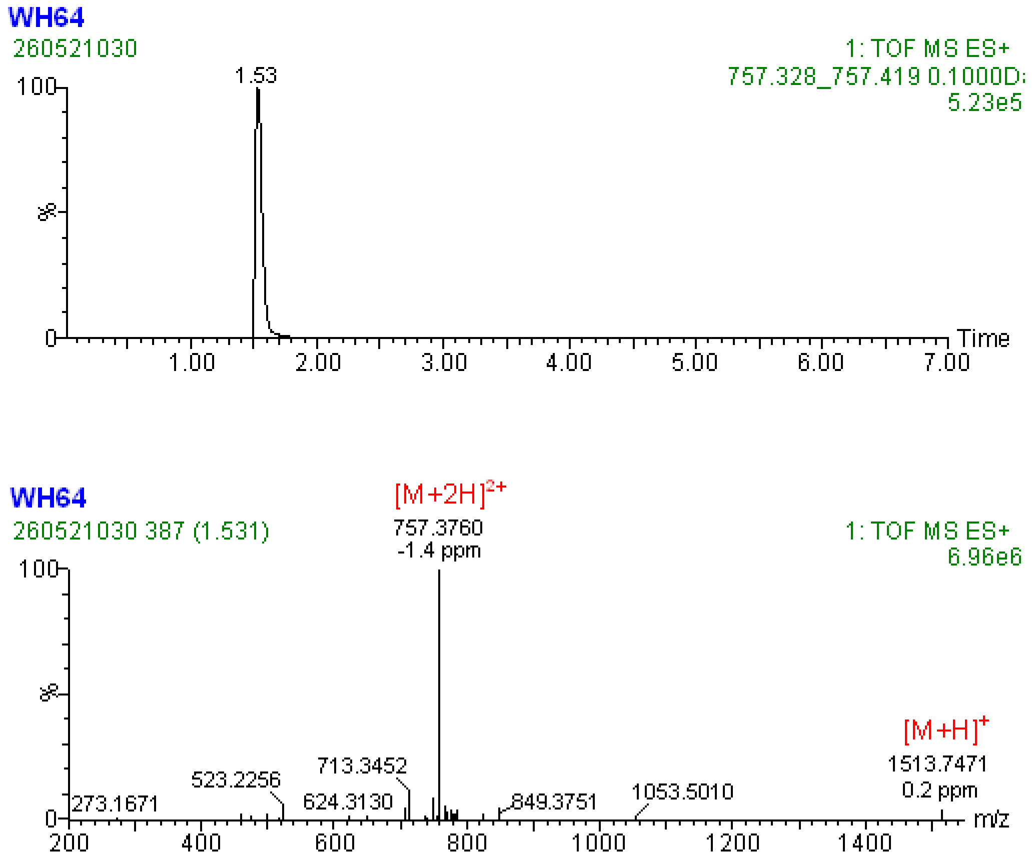

WH64
260521030
1: TOF MS ES+
757.328_757.419 0.1000Da
5.23e5
1.53
100
%
0
Time
1.00
2.00
3.00
4.00
5.00
6.00
7.00
WH64
260521030 387 (1.531)
[M+2H]2+
757.3760
-1.4 ppm
1: TOF MS ES+
6.96e6
[M+H]+
1513.7471
0.2 ppm
273.1671
523.2256
624.3130
713.3452
849.3751
1053.5010
m/z
200
400
600
800
1000
1200
1400

B

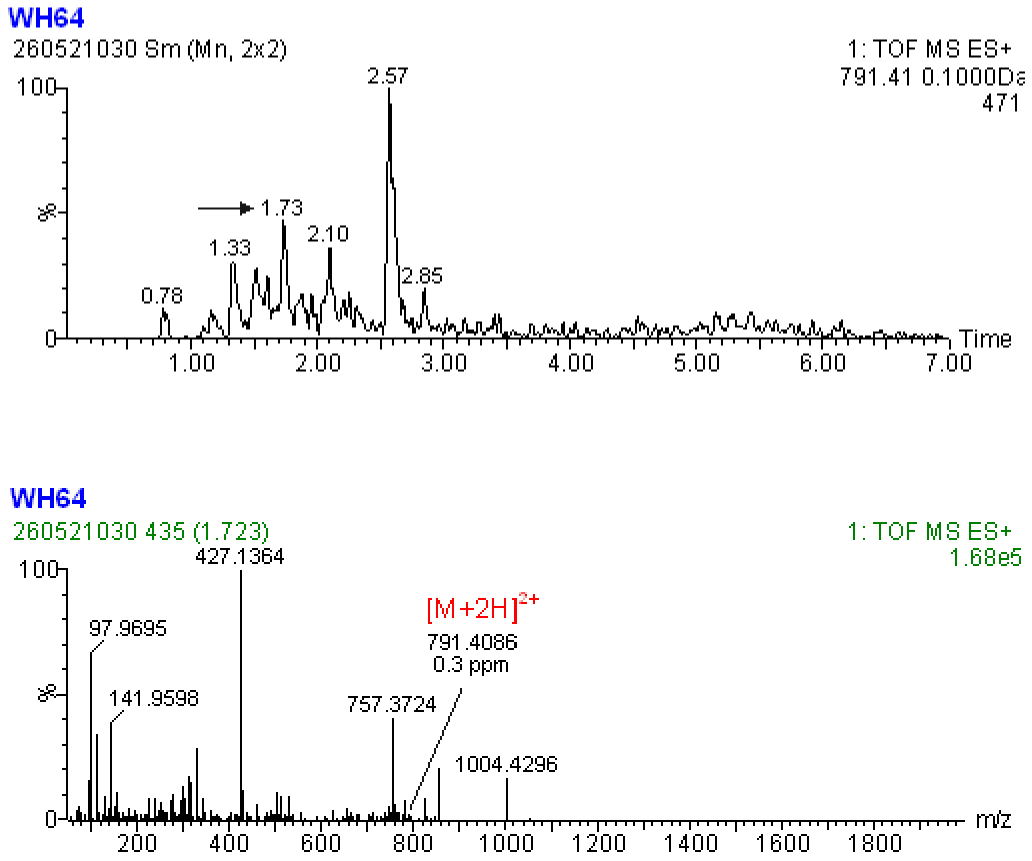

WH64
260521030 Sm (Mn, 2x2)
1: TOF MS ES+
791.41 0.1000Da
471
2.57
1.73
1.33
2.10
0.78
2.85
Time
1.00
2.00
3.00
4.00
5.00
6.00
7.00
WH64
260521030 435 (1.723)
1: TOF MS ES+
1.68e5
427.1364
97.9695
141.9598
[M+2H]2+
791.4086
0.3 ppm
757.3724
1004.4296
m/z
200
400
600
800
1000
1200
1400
1600
1800

Figure S13: LC HRMS of the biochemical reaction of peptide H-TLRESTAMYPFQA-NH_2 (**5**) and AutF in the presence of DMAPP. A) LCMS of the unprocessed substrate, B) LCMS of the prenylated product.

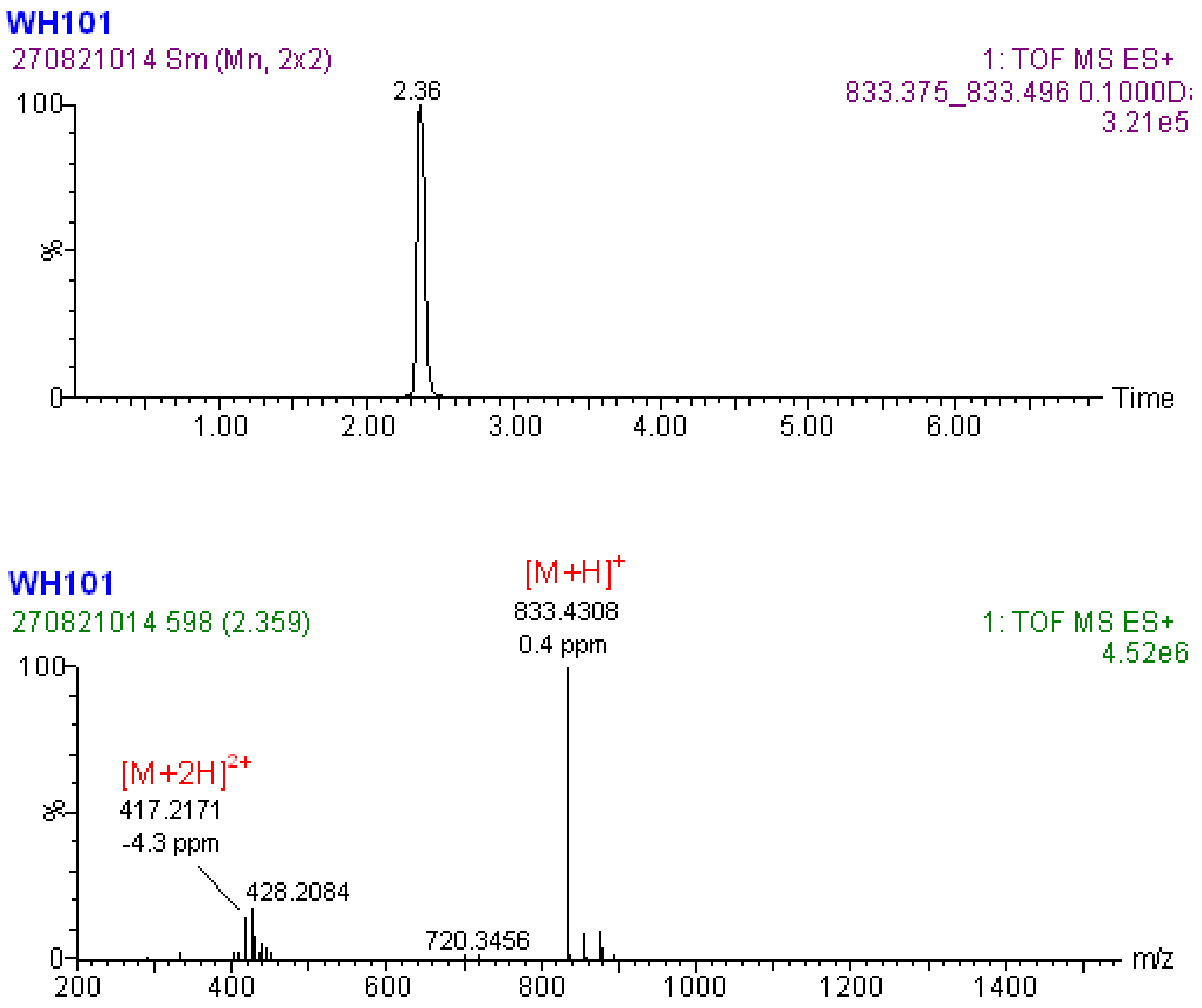

Figure S14: LC HRMS of the unprocessed substrate in the reaction mixture of *Cyclo*[-LGPFrFD] (**6**) and AutF in the presence of DMAPP.

A

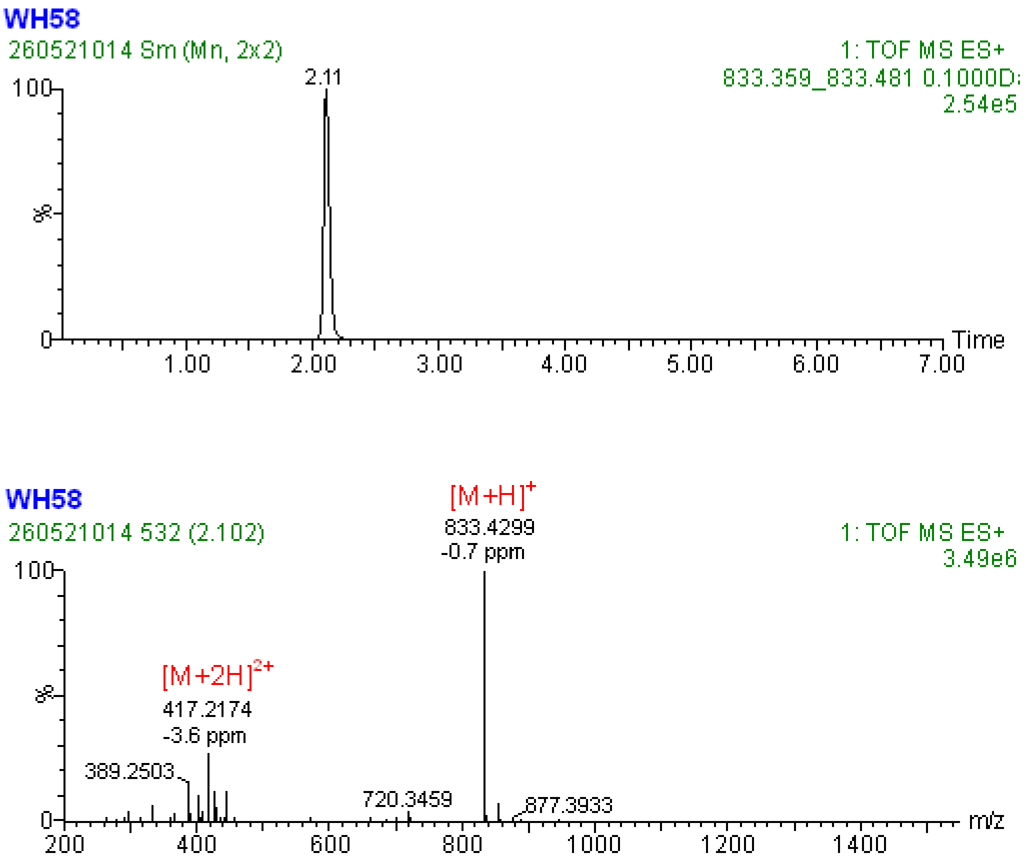
WH58
260521014 Sm (Mn, 2x2)
1: TOF MS ES+
833.359_833.481 0.1000Da
2.54e5
2.11
100
%
0
1.00
2.00
3.00
4.00
5.00
6.00
7.00
Time
WH58
260521014 532 (2.102)
1: TOF MS ES+
3.49e6
[M+H]+
833.4299
-0.7 ppm
[M+2H]2+
417.2174
-3.6 ppm
389.2503
720.3459
877.3933
100
%
0
200
400
600
800
1000
1200
1400
m/z

B

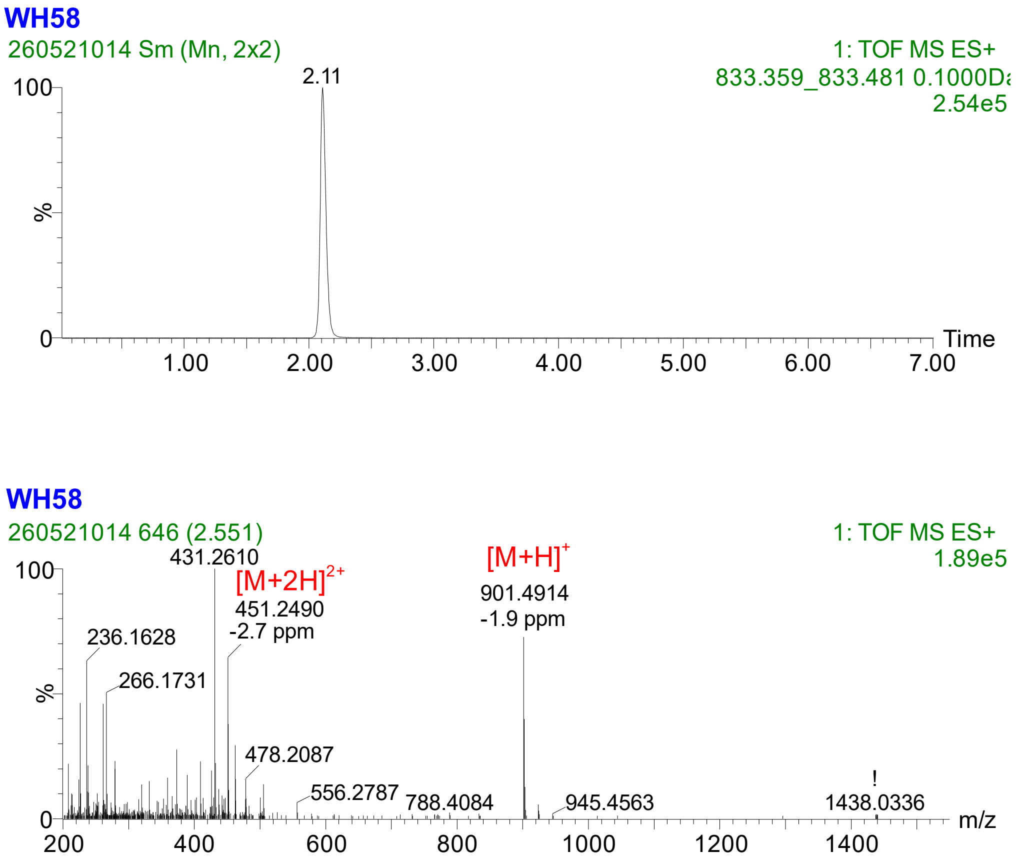
WH58
260521014 Sm (Mn, 2x2)
1: TOF MS ES+
833.359_833.481 0.1000Da
2.54e5
2.11
100
%
0
1.00
2.00
3.00
4.00
5.00
6.00
7.00
Time
WH58
260521014 646 (2.551)
1: TOF MS ES+
1.89e5
431.2610
[M+2H]2+
451.2490
-2.7 ppm
[M+H]+
901.4914
-1.9 ppm
236.1628
266.1731
478.2087
556.2787
788.4084
945.4563
1438.0336
100
%
0
200
400
600
800
1000
1200
1400
m/z

Figure S15: LC HRMS of the biochemical reaction of peptide *cyclo*[-LGPFRFD] (**7**) and AutF in the presence of DMAPP. A) LCMS of the unprocessed substrate, B) LCMS of the prenylated product.

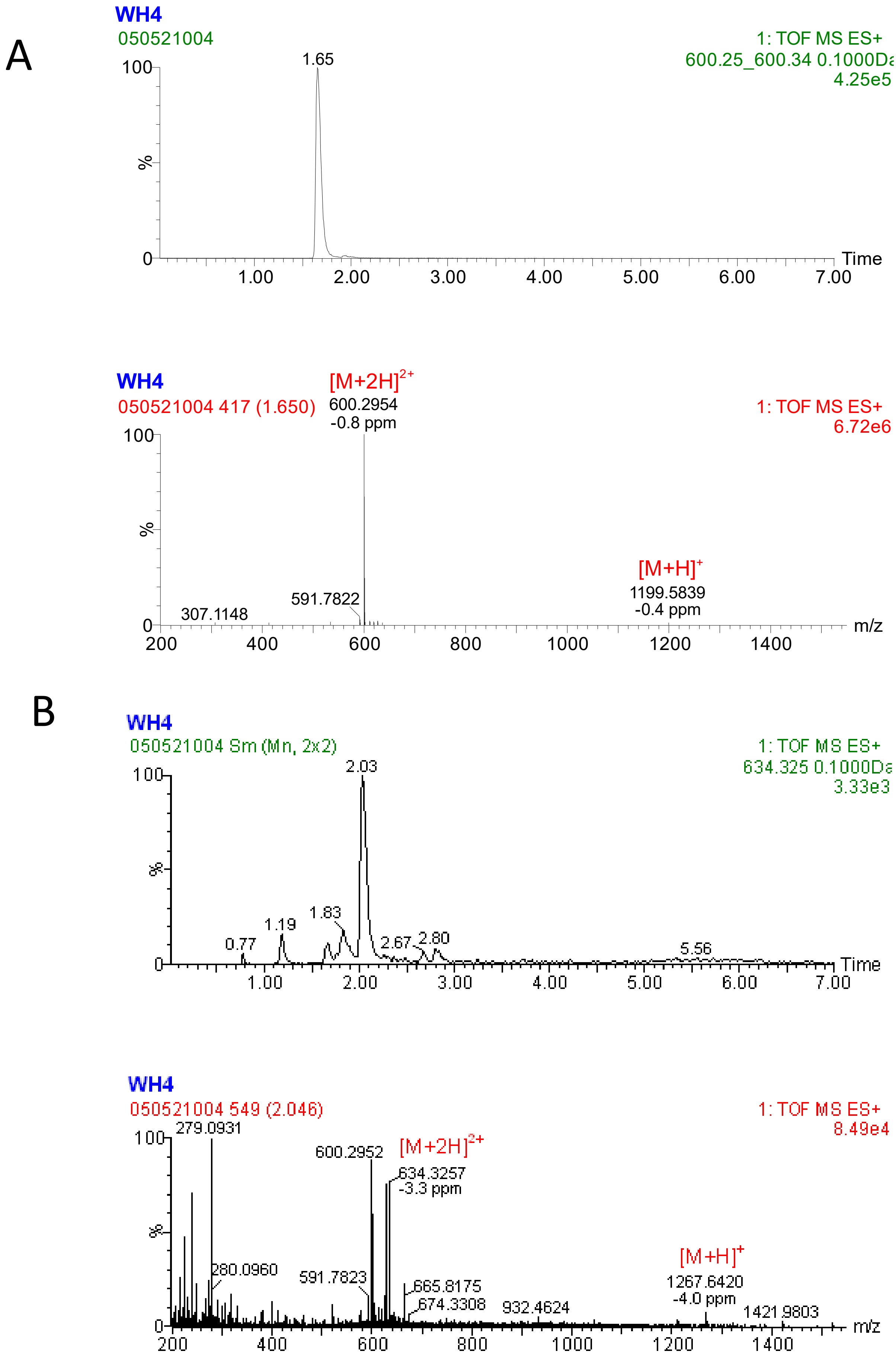
A
WH4
050521004
1: TOF MS ES+
600.25_600.34 0.1000Da
4.25e5
1.65
100
%
0
Time
1.00
2.00
3.00
4.00
5.00
6.00
7.00
WH4
050521004 417 (1.650)
[M+2H]2+
600.2954
-0.8 ppm
1: TOF MS ES+
6.72e6
[M+H]+
1199.5839
-0.4 ppm
591.7822
307.1148
m/z
200
400
600
800
1000
1200
1400
B
WH4
050521004 Sm (Mn, 2x2)
1: TOF MS ES+
634.325 0.1000Da
3.33e3
2.03
1.83
1.19
0.77
2.67
2.80
5.56
WH4
050521004 549 (2.046)
1: TOF MS ES+
8.49e4
279.0931
600.2952
[M+2H]2+
634.3257
-3.3 ppm
280.0960
591.7823
665.8175
674.3308
932.4624
[M+H]+
1267.6420
-4.0 ppm
1421.9803

Figure S16: LC HRMS of the biochemical reaction of peptide H-FrFDLGpAYD-NH_2 (**8**) and AutF in the presence of DMAPP. A) LCMS of the unprocessed substrate, B) LCMS of the prenylated product.

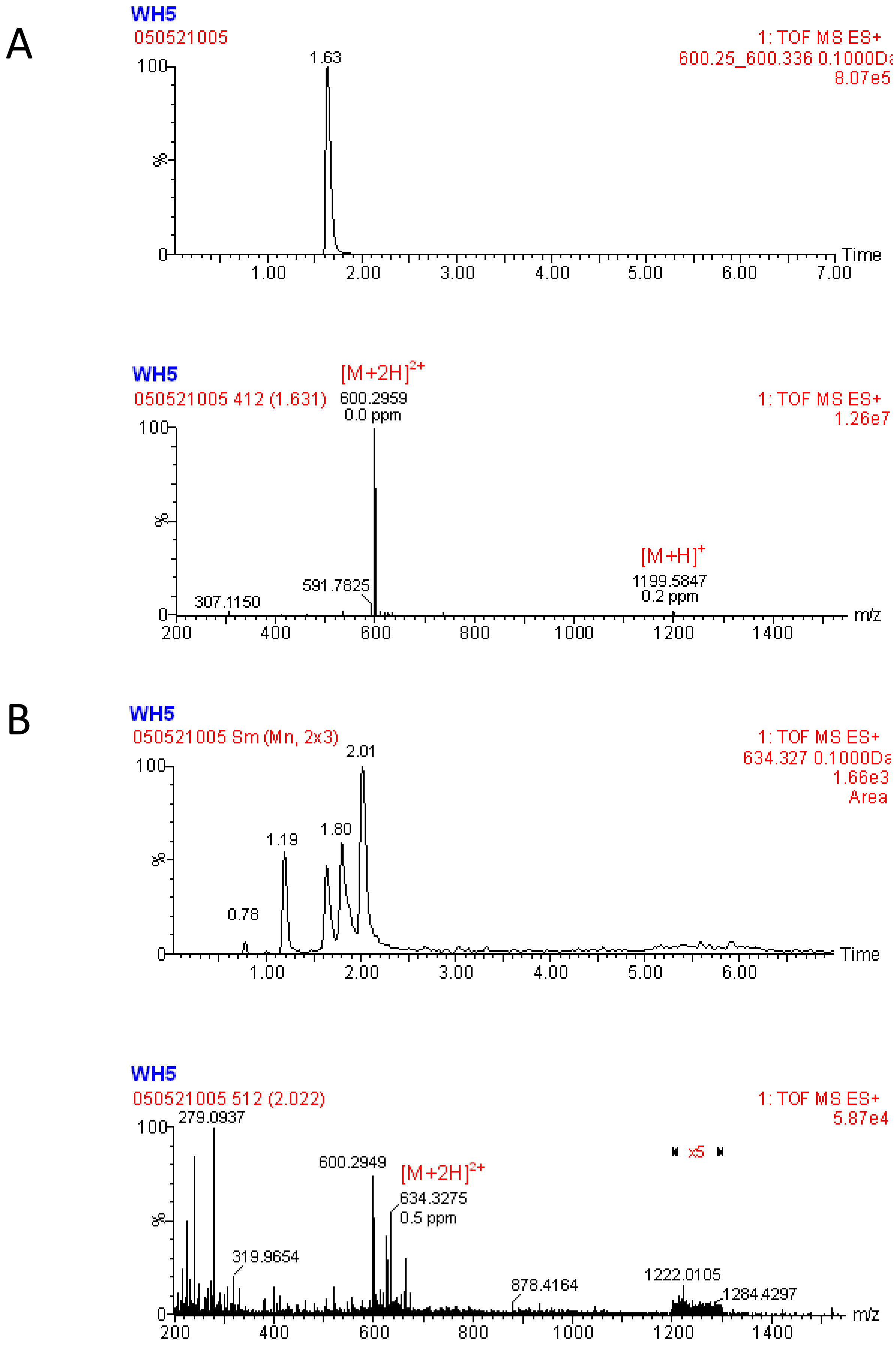
A
WH5
050521005
1: TOF MS ES+
600.25_600.336 0.1000Da
8.07e5
1.63
100
%
0
Time
1.00 2.00 3.00 4.00 5.00 6.00 7.00
WH5
050521005 412 (1.631)
[M+2H]2+
600.2959
0.0 ppm
1: TOF MS ES+
1.26e7
[M+H]+
1199.5847
0.2 ppm
591.7825
307.1150
m/z
200 400 600 800 1000 1200 1400
B
WH5
050521005 Sm (Mn, 2x3)
1: TOF MS ES+
634.327 0.1000Da
1.66e3
Area
2.01
1.80
1.19
0.78
Time
1.00 2.00 3.00 4.00 5.00 6.00
WH5
050521005 512 (2.022)
1: TOF MS ES+
5.87e4
279.0937
x5
600.2949
[M+2H]2+
634.3275
0.5 ppm
319.9654
878.4164
1222.0105
1284.4297
m/z
200 400 600 800 1000 1200 1400

Figure S17: LC HRMS of the biochemical reaction of peptide H-FrFDLGPAYD-NH_2 (**9**) and AutF in the presence of DMAPP. A) LCMS of the unprocessed substrate, B) LCMS of the prenylated product.

A

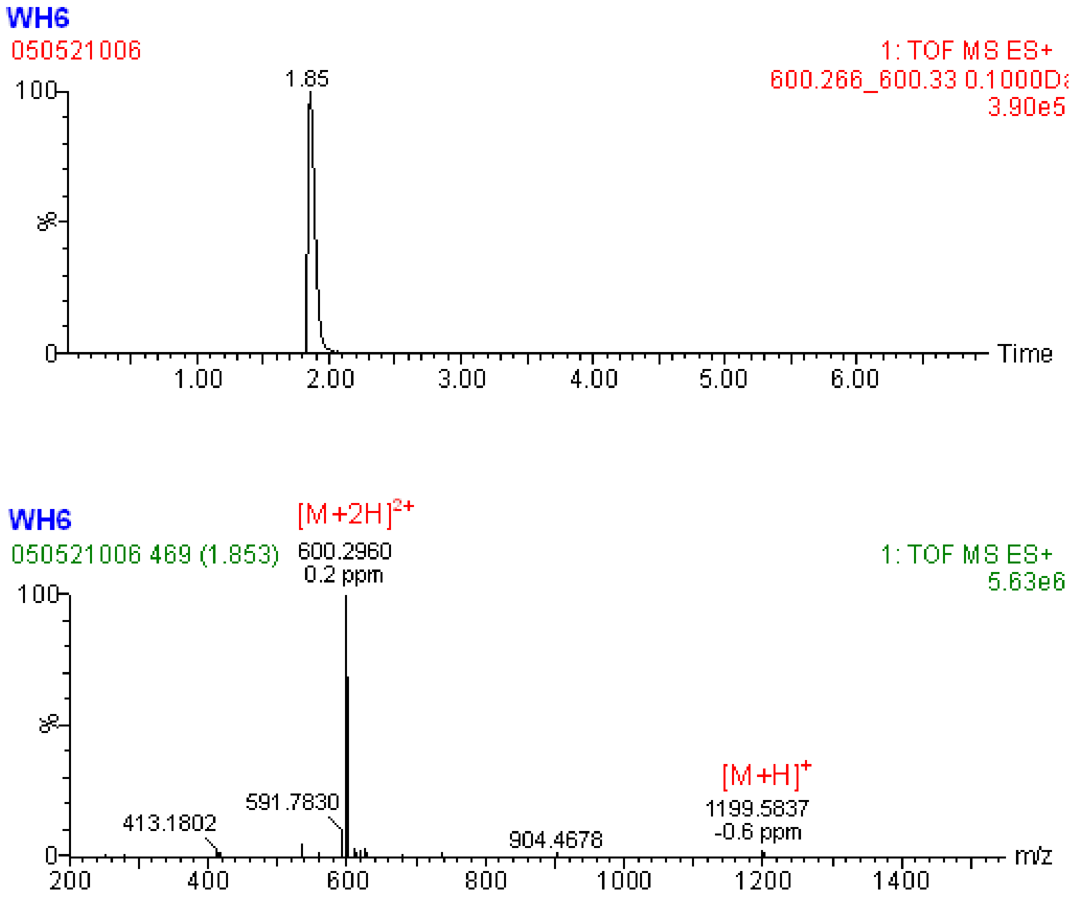

WH6
050521006
1: TOF MS ES+
600.266_600.33 0.1000Da
3.90e5
1.85
100
%
0
Time
1.00
2.00
3.00
4.00
5.00
6.00
WH6
050521006 469 (1.853)
[M+2H]$^{2+}$
600.2960
0.2 ppm
1: TOF MS ES+
5.63e6
[M+H]$^{+}$
1199.5837
-0.6 ppm
591.7830
413.1802
904.4678
m/z
200
400
600
800
1000
1200
1400

B

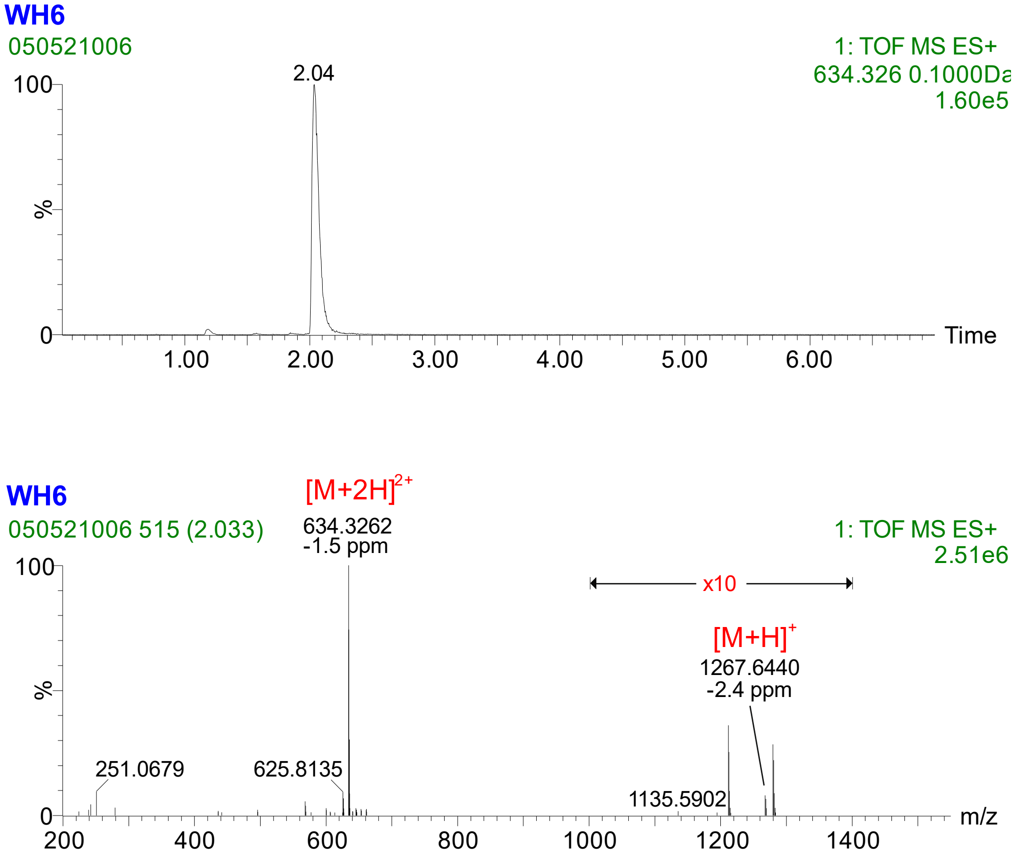

WH6
050521006
1: TOF MS ES+
634.326 0.1000Da
1.60e5
2.04
100
%
0
Time
1.00
2.00
3.00
4.00
5.00
6.00
WH6
050521006 515 (2.033)
[M+2H]$^{2+}$
634.3262
-1.5 ppm
1: TOF MS ES+
2.51e6
x10
[M+H]$^{+}$
1267.6440
-2.4 ppm
251.0679
625.8135
1135.5902
m/z
200
400
600
800
1000
1200
1400

Figure S18: LC HRMS of the biochemical reaction of peptide H-FRFDLGPAYD-NH_2 (**10**) and AutF in the presence of DMAPP. A) LCMS of the unprocessed substrate, B) LCMS of the prenylated product.

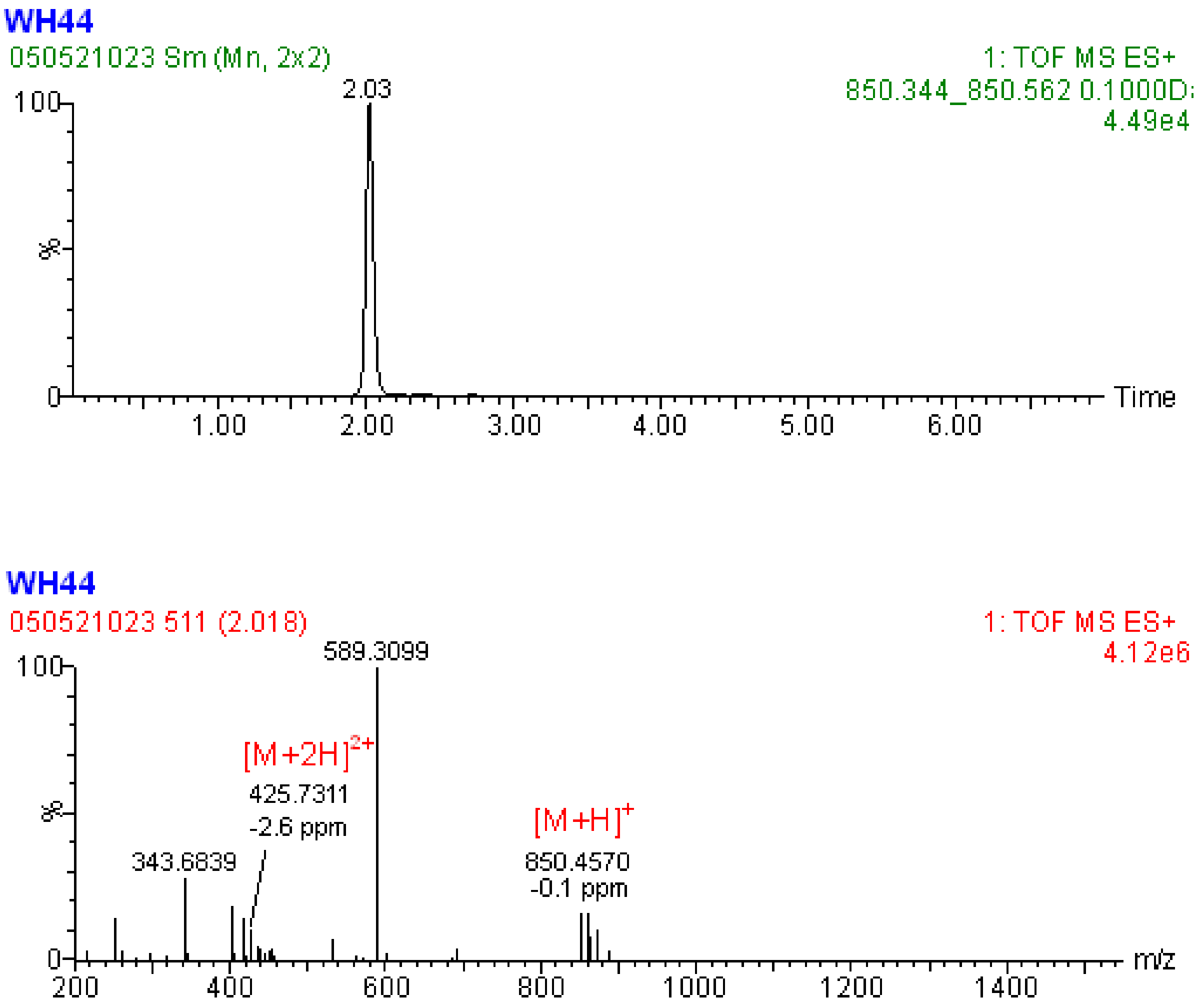

Figure S19: LC HRMS of the unprocessed substrate in the reaction mixture of H-RFDLGPF-NH_2 (**11**) and AutF in the presence of DMAPP.

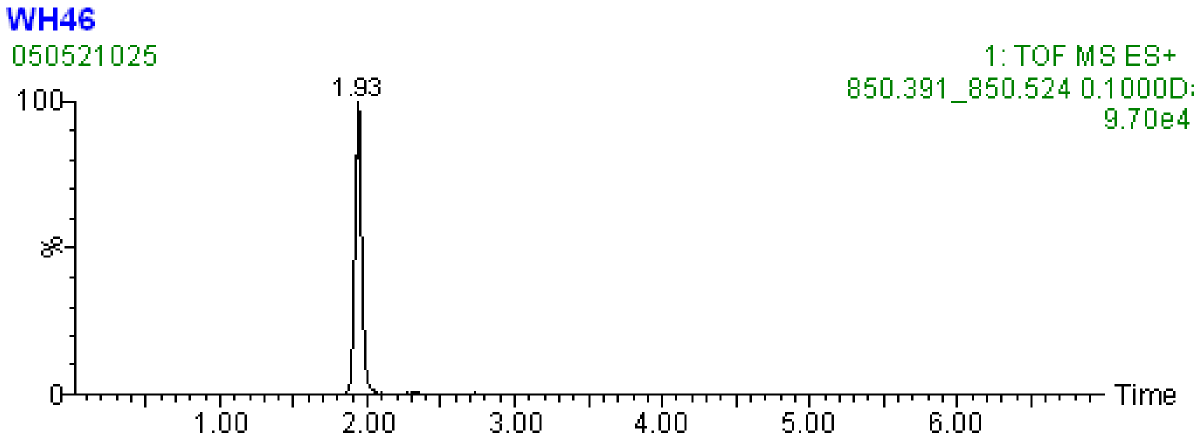

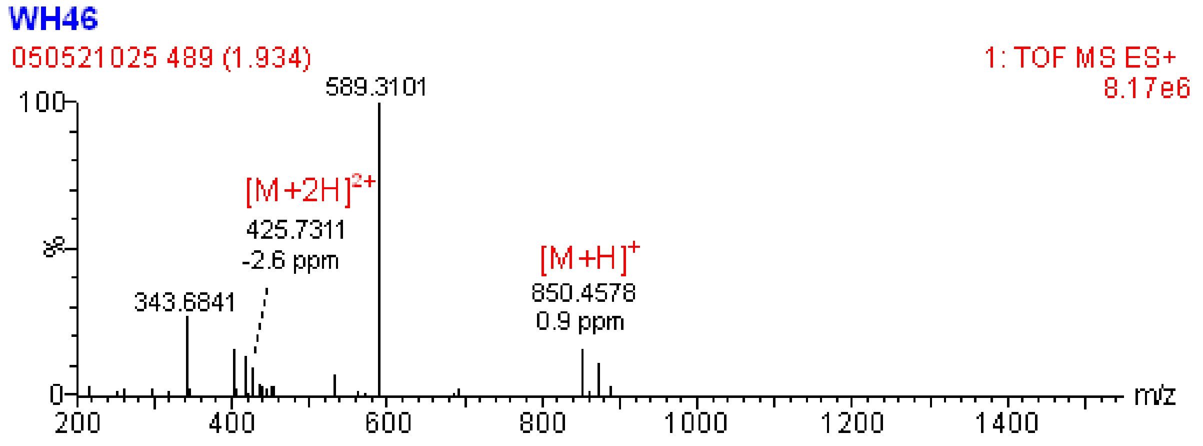

Figure S20: LC HRMS of the unprocessed substrate in the reaction mixture of H-rFDLGPF-NH_2 (**12**) and AutF in the presence of DMAPP.

A

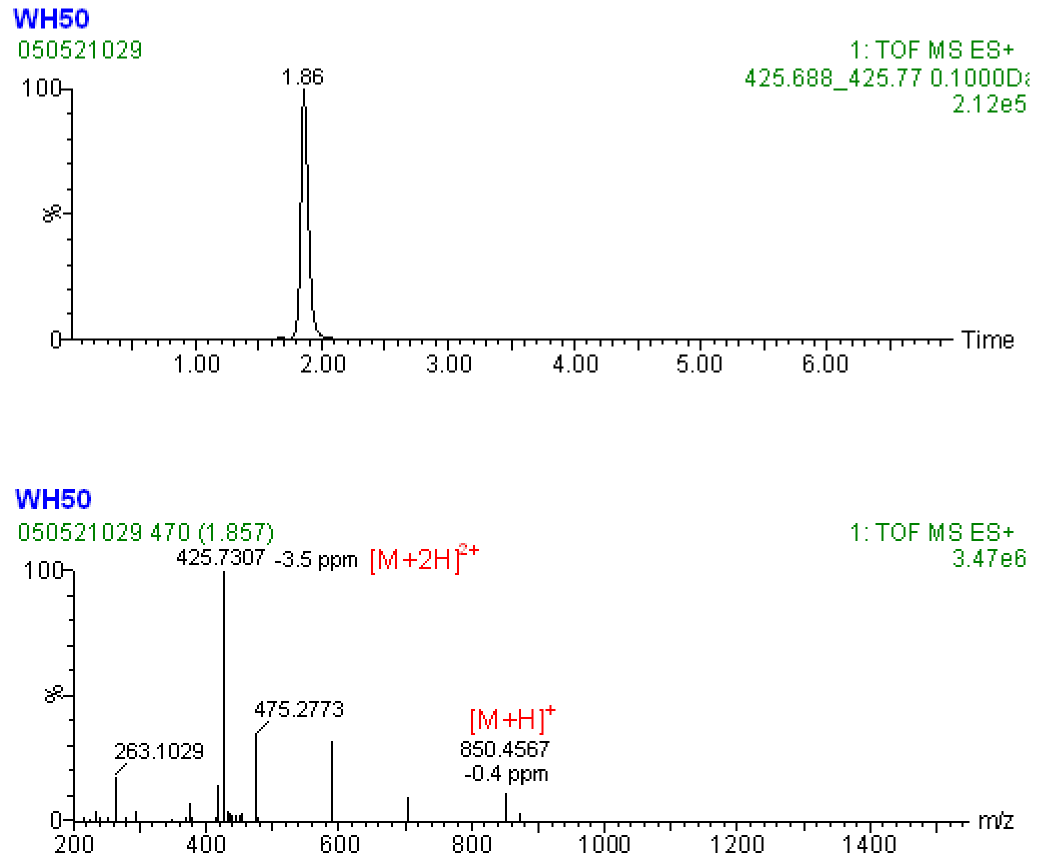
WH50
050521029
1: TOF MS ES+
425.688_425.77 0.1000Da
2.12e5
1.86
100
%
0
1.00
2.00
3.00
4.00
5.00
6.00
Time
WH50
050521029 470 (1.857)
1: TOF MS ES+
3.47e6
425.7307 -3.5 ppm [M+2H]2+
475.2773
263.1029
[M+H]+
850.4567
-0.4 ppm
100
%
0
200
400
600
800
1000
1200
1400
m/z

B

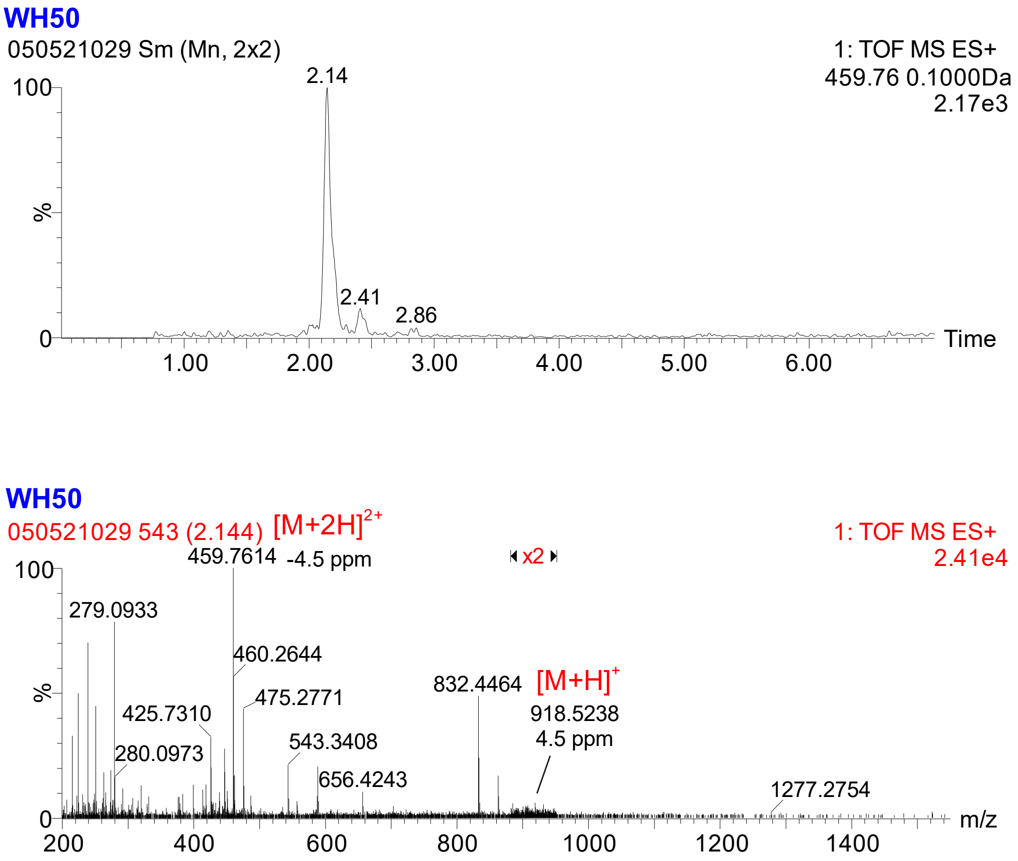
WH50
050521029 Sm (Mn, 2x2)
1: TOF MS ES+
459.76 0.1000Da
2.17e3
2.14
2.41
2.86
100
%
0
1.00
2.00
3.00
4.00
5.00
6.00
Time
WH50
050521029 543 (2.144) [M+2H]2+
1: TOF MS ES+
2.41e4
459.7614 -4.5 ppm
x2
279.0933
460.2644
475.2771
425.7310
280.0973
543.3408
656.4243
832.4464
[M+H]+
918.5238
4.5 ppm
1277.2754
100
%
0
200
400
600
800
1000
1200
1400
m/z

Figure S21: LC HRMS of the biochemical reaction of peptide H-FDLGPFR-NH_2 (**13**) and AutF in the presence of DMAPP. A) LCMS of the unprocessed substrate, B) LCMS of the prenylated product.

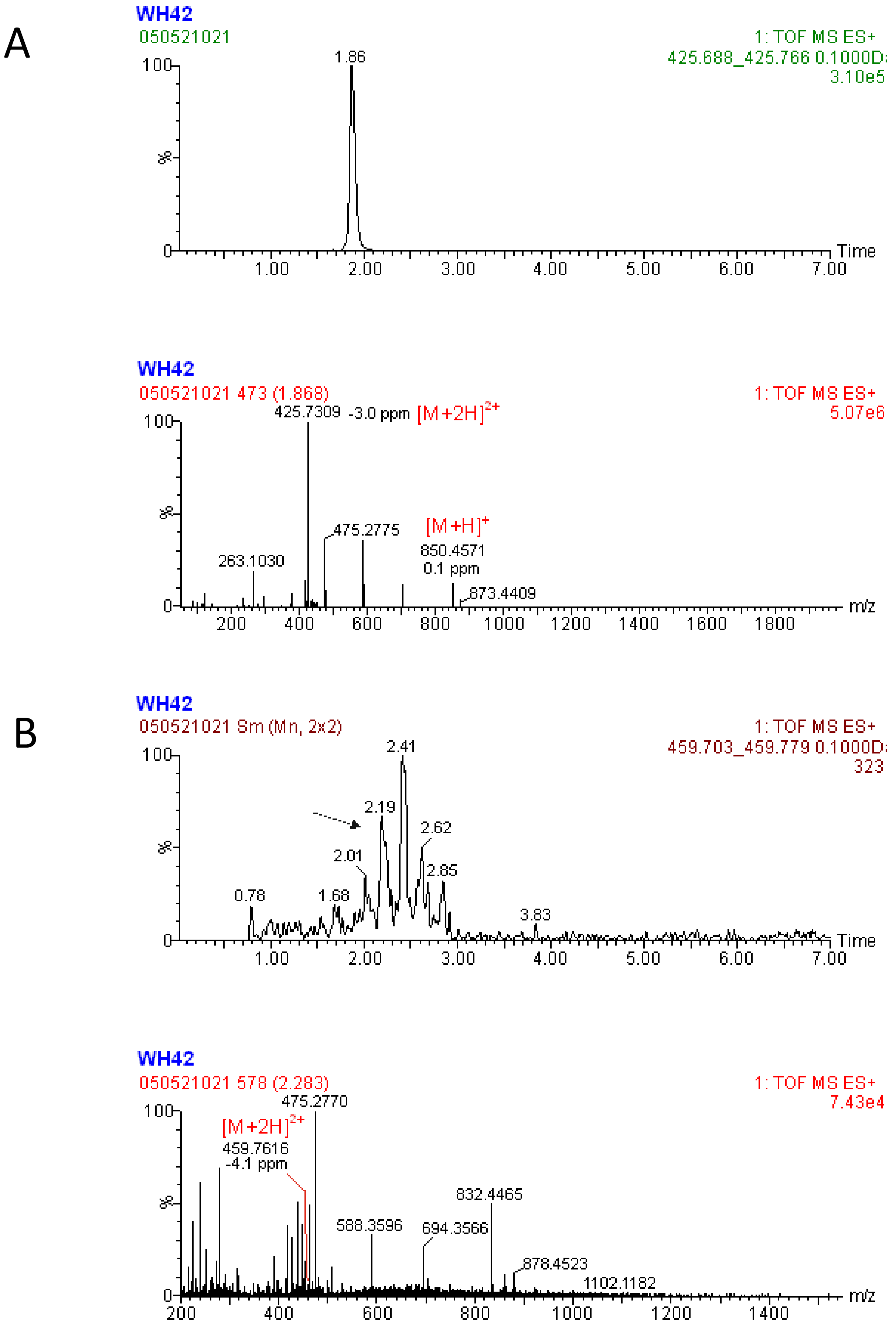
A
WH42
050521021
1: TOF MS ES+
425.688_425.766 0.1000D:
3.10e5
1.86
100
%
0
Time
1.00
2.00
3.00
4.00
5.00
6.00
7.00
WH42
050521021 473 (1.868)
1: TOF MS ES+
5.07e6
425.7309 -3.0 ppm [M+2H]2+
475.2775
[M+H]+
850.4571
0.1 ppm
263.1030
873.4409
m/z
200
400
600
800
1000
1200
1400
1600
1800
B
WH42
050521021 Sm (Mn, 2x2)
1: TOF MS ES+
459.703_459.779 0.1000D:
323
2.41
2.19
2.62
2.01
2.85
0.78
1.68
3.83
Time
WH42
050521021 578 (2.283)
1: TOF MS ES+
7.43e4
475.2770
[M+2H]2+
459.7616
-4.1 ppm
832.4465
588.3596
694.3566
878.4523
1102.1182
m/z

Figure S22: LC HRMS of the biochemical reaction of peptide H-FDLGPFr-NH_2 (**14**) and AutF in the presence of DMAPP. A) LCMS of the unprocessed substrate, B) LCMS of the prenylated product.

A

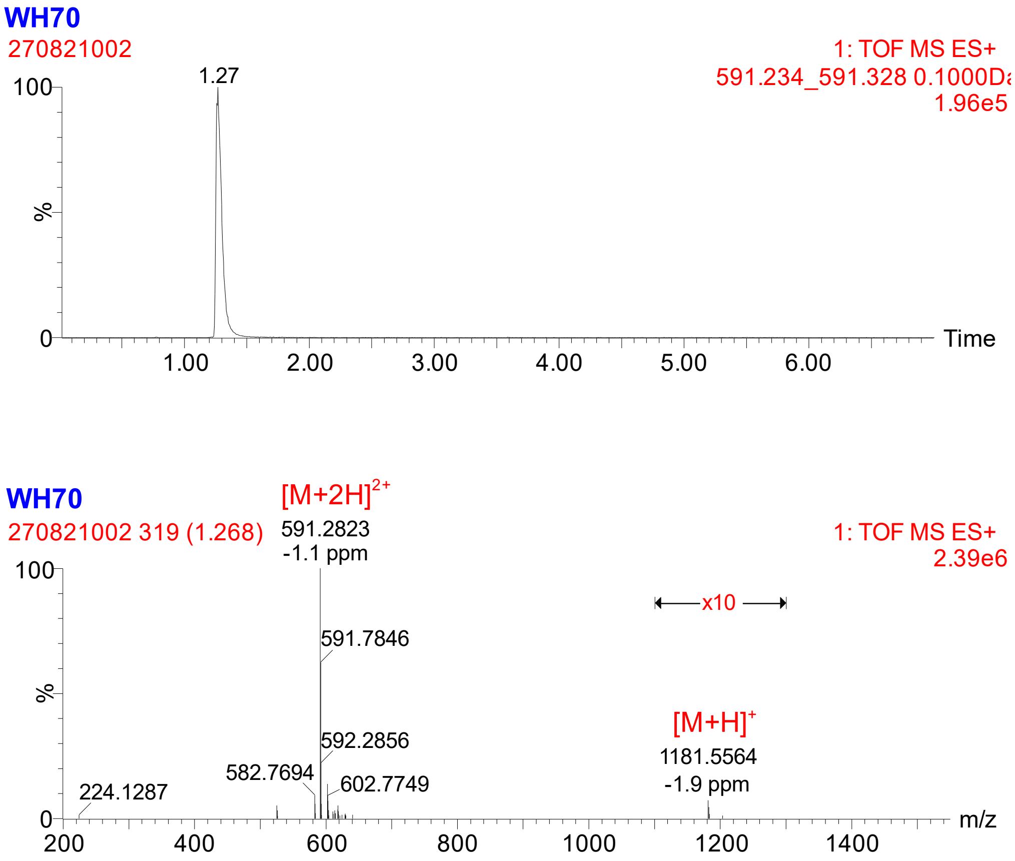

WH70
270821002
1: TOF MS ES+
591.234_591.328 0.1000Da
1.96e5
1.27
100
%
0
1.00
2.00
3.00
4.00
5.00
6.00
Time
WH70
270821002 319 (1.268)
[M+2H]2+
591.2823
-1.1 ppm
1: TOF MS ES+
2.39e6
x10
591.7846
592.2856
582.7694
602.7749
224.1287
[M+H]+
1181.5564
-1.9 ppm
200
400
600
800
1000
1200
1400
m/z

B

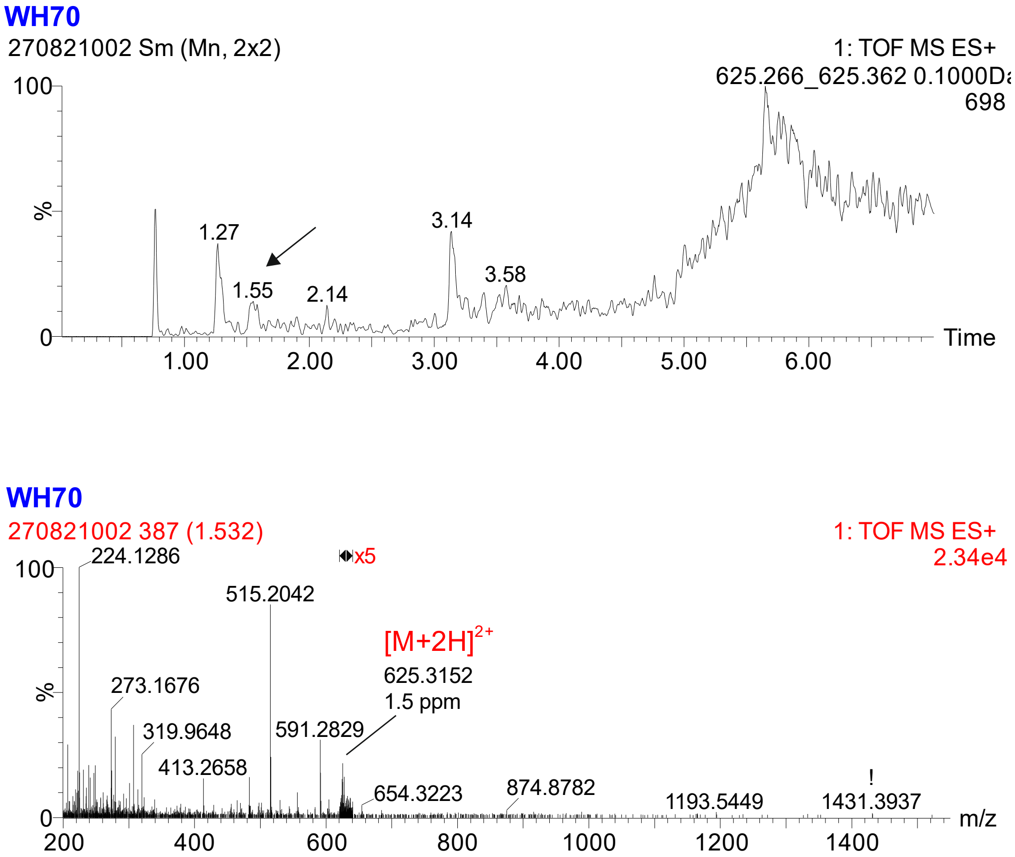

WH70
270821002 Sm (Mn, 2x2)
1: TOF MS ES+
625.266_625.362 0.1000Da
698
1.27
1.55
2.14
3.14
3.58
Time
WH70
270821002 387 (1.532)
1: TOF MS ES+
2.34e4
x5
224.1286
515.2042
[M+2H]2+
625.3152
1.5 ppm
273.1676
319.9648
591.2829
413.2658
654.3223
874.8782
1193.5449
1431.3937
m/z

Figure S23: LC HRMS of the biochemical reaction of peptide H-FREDLGPAYD-NH_2 (**15**) and AutF in the presence of DMAPP. A) LCMS of the unprocessed substrate, B) LCMS of the prenylated product.

A

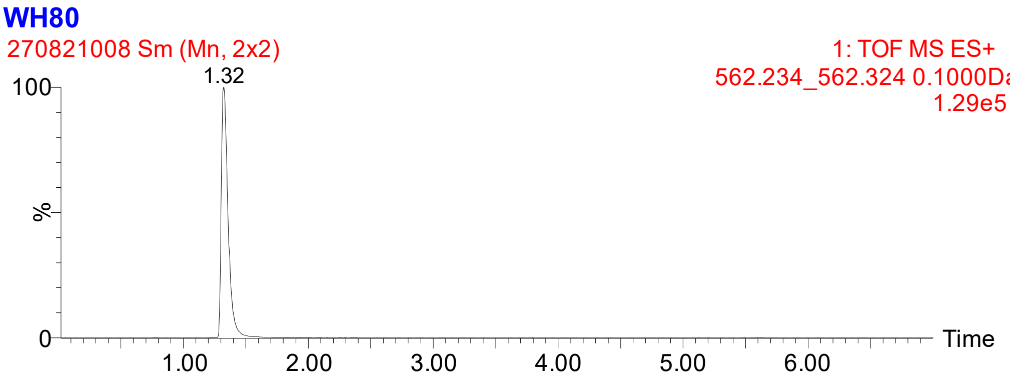
WH80
270821008 Sm (Mn, 2x2)
1: TOF MS ES+
562.234_562.324 0.1000Da
1.29e5
1.32
100
%
0
Time
1.00
2.00
3.00
4.00
5.00
6.00

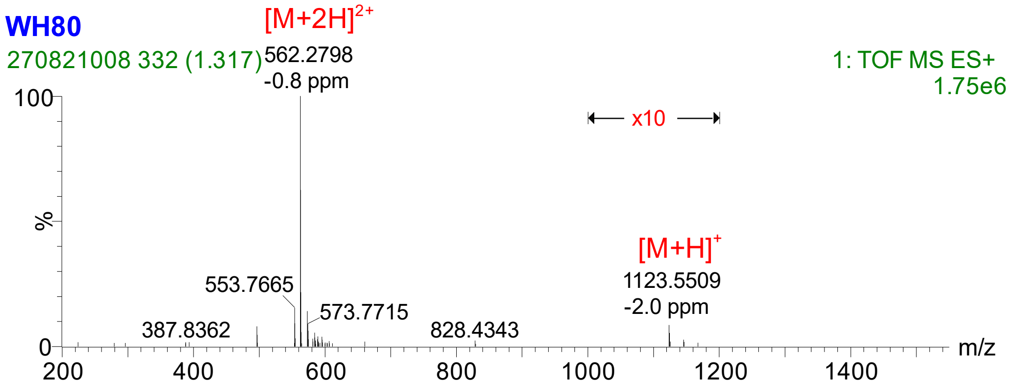
WH80
270821008 332 (1.317)
[M+2H]2+
562.2798
-0.8 ppm
1: TOF MS ES+
1.75e6
x10
[M+H]+
1123.5509
-2.0 ppm
553.7665
573.7715
387.8362
828.4343
m/z
200
400
600
800
1000
1200
1400

B

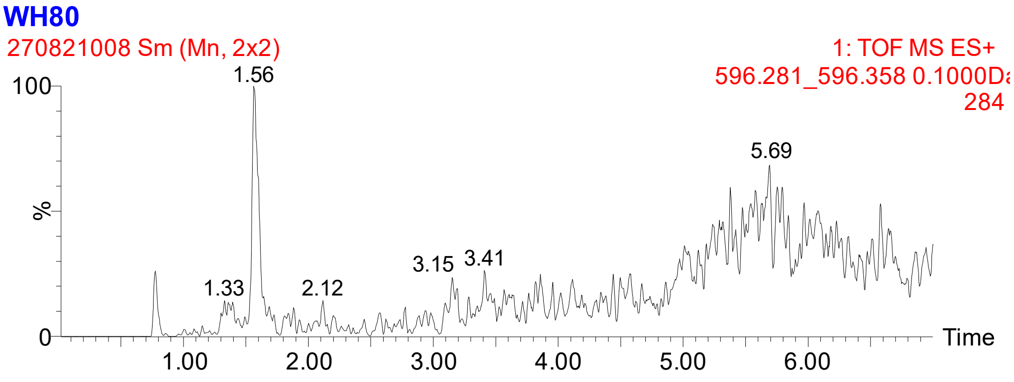
WH80
270821008 Sm (Mn, 2x2)
1: TOF MS ES+
596.281_596.358 0.1000Da
284
1.56
5.69
3.15
3.41
1.33
2.12
Time
1.00
2.00
3.00
4.00
5.00
6.00

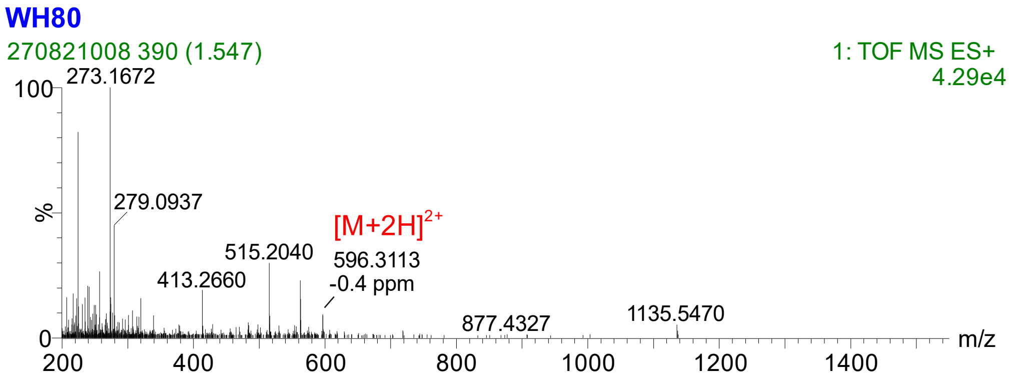
WH80
270821008 390 (1.547)
1: TOF MS ES+
4.29e4
273.1672
279.0937
[M+2H]2+
596.3113
-0.4 ppm
515.2040
413.2660
877.4327
1135.5470
m/z
200
400
600
800
1000
1200
1400

Figure S24: LC HRMS of the biochemical reaction of peptide H-FRADLGPAYD-NH_2 (**16**) and AutF in the presence of DMAPP. A) LCMS of the unprocessed substrate, B) LCMS of the prenylated product.

A

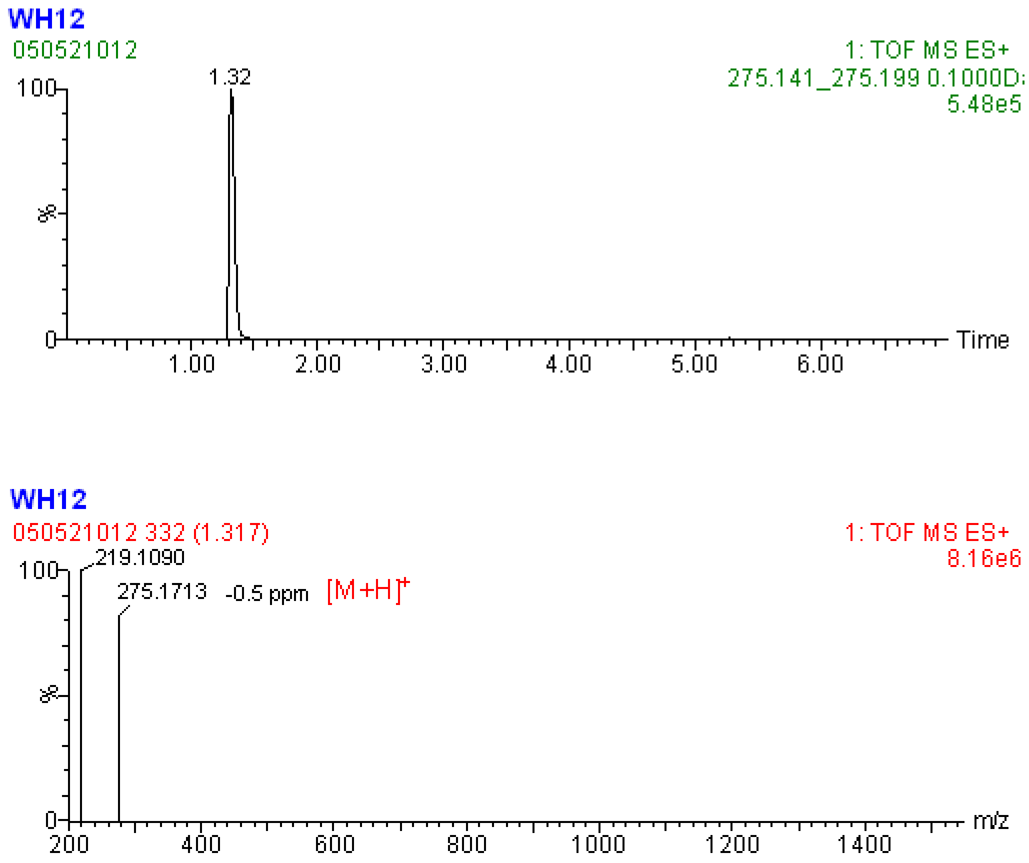
WH12
050521012
1: TOF MS ES+
275.141_275.199 0.1000D
5.48e5
1.32
100
%
0
1.00
2.00
3.00
4.00
5.00
6.00
Time
WH12
050521012 332 (1.317)
1: TOF MS ES+
8.16e6
219.1090
275.1713 -0.5 ppm [M+H]+
100
%
0
200
400
600
800
1000
1200
1400
m/z

B

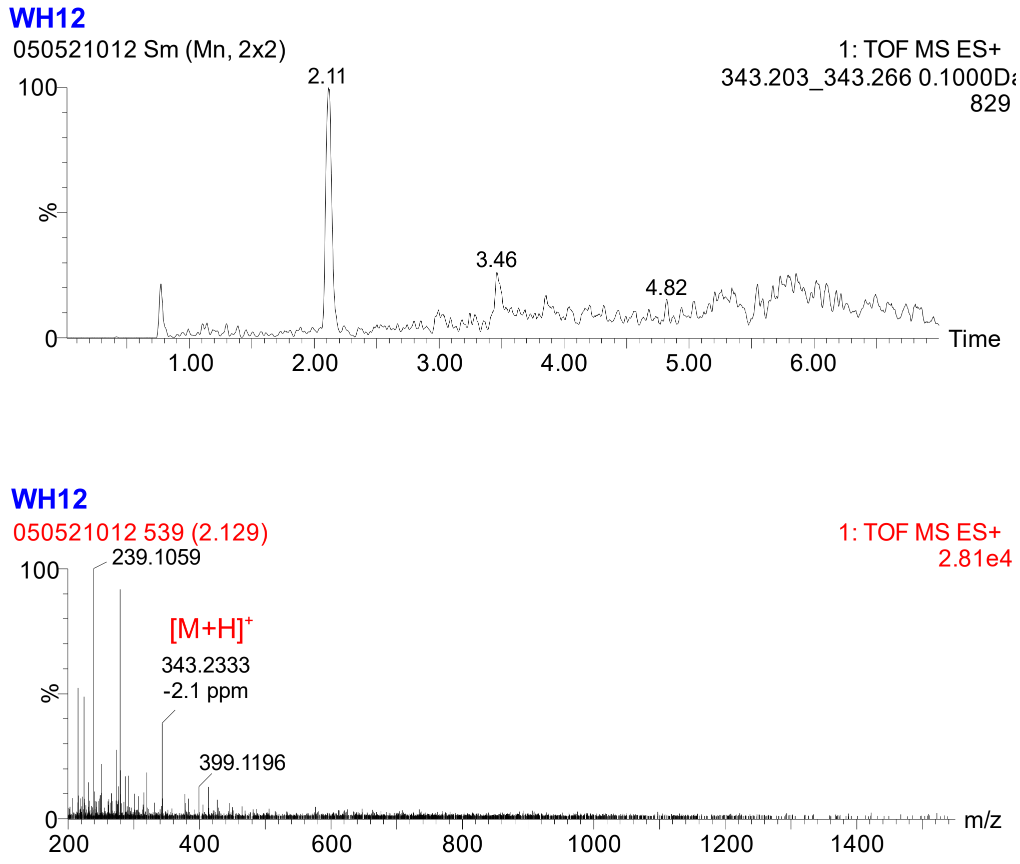
WH12
050521012 Sm (Mn, 2x2)
1: TOF MS ES+
343.203_343.266 0.1000D
829
2.11
3.46
4.82
100
%
0
1.00
2.00
3.00
4.00
5.00
6.00
Time
WH12
050521012 539 (2.129)
1: TOF MS ES+
2.81e4
239.1059
[M+H]+
343.2333
-2.1 ppm
399.1196
100
%
0
200
400
600
800
1000
1200
1400
m/z

Figure S25: LC HRMS of the biochemical reaction of the substrate *N*-α-Boc-L-arginine (**17**) and AutF in the presence of DMAPP. A) LCMS of the unprocessed substrate, B) LCMS of the prenylated product.

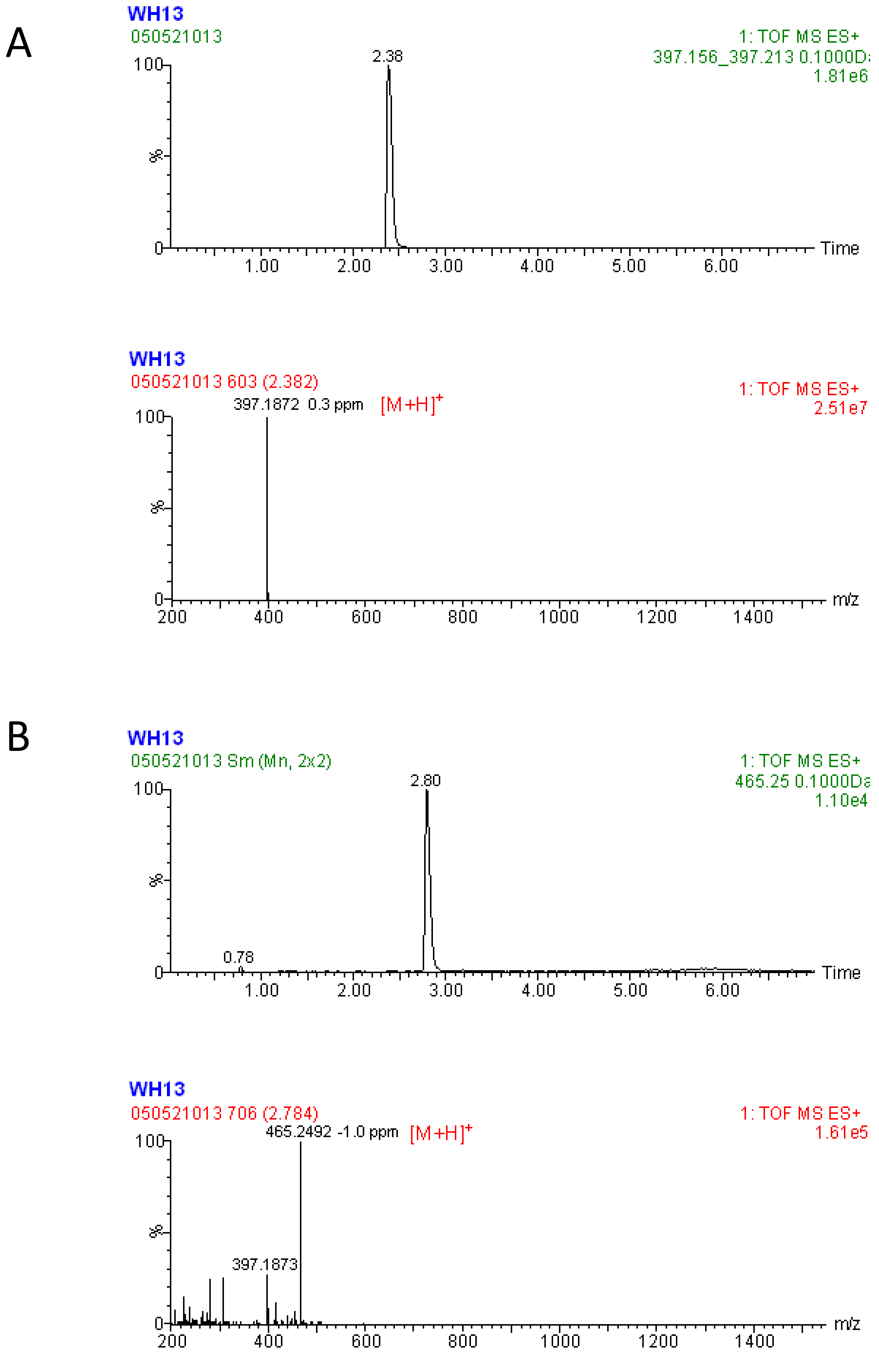
A
WH13
050521013
1: TOF MS ES+
397.156_397.213 0.1000Da
1.81e6
2.38
100
%
0
Time
1.00
2.00
3.00
4.00
5.00
6.00
WH13
050521013 603 (2.382)
1: TOF MS ES+
2.51e7
397.1872 0.3 ppm
[M+H]+
m/z
200
400
600
800
1000
1200
1400
B
WH13
050521013 Sm (Mn, 2x2)
1: TOF MS ES+
465.25 0.1000Da
1.10e4
2.80
0.78
WH13
050521013 706 (2.784)
1: TOF MS ES+
1.61e5
465.2492 -1.0 ppm
[M+H]+
397.1873

Figure S26: LC HRMS of the biochemical reaction of the substrate *N*-α-Fmoc-L-arginine (**18**) and AutF in the presence of DMAPP. A) LCMS of the unprocessed substrate, B) LCMS of the prenylated product.

A

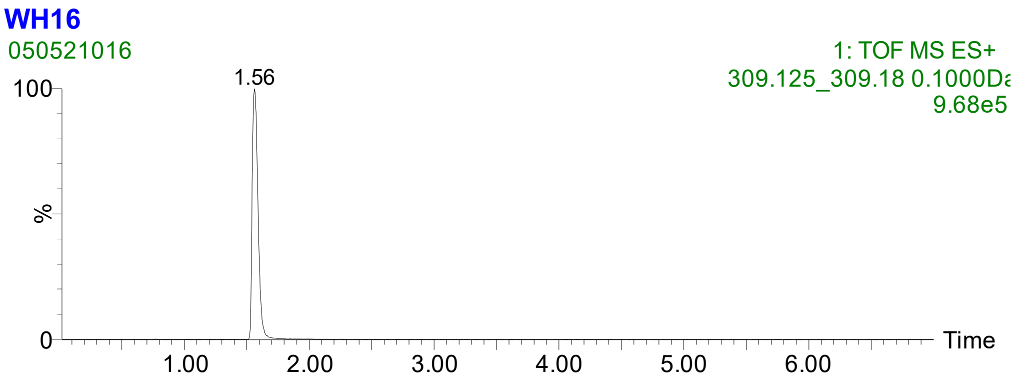
WH16
050521016
1: TOF MS ES+
309.125_309.18 0.1000Da
9.68e5
1.56
100
%
0
Time
1.00
2.00
3.00
4.00
5.00
6.00
WH16
050521016 394 (1.563)
1: TOF MS ES+
1.22e7
309.1558 0.1 ppm [M+H]+
100
%
0
m/z
200
400
600
800
1000
1200
1400

B

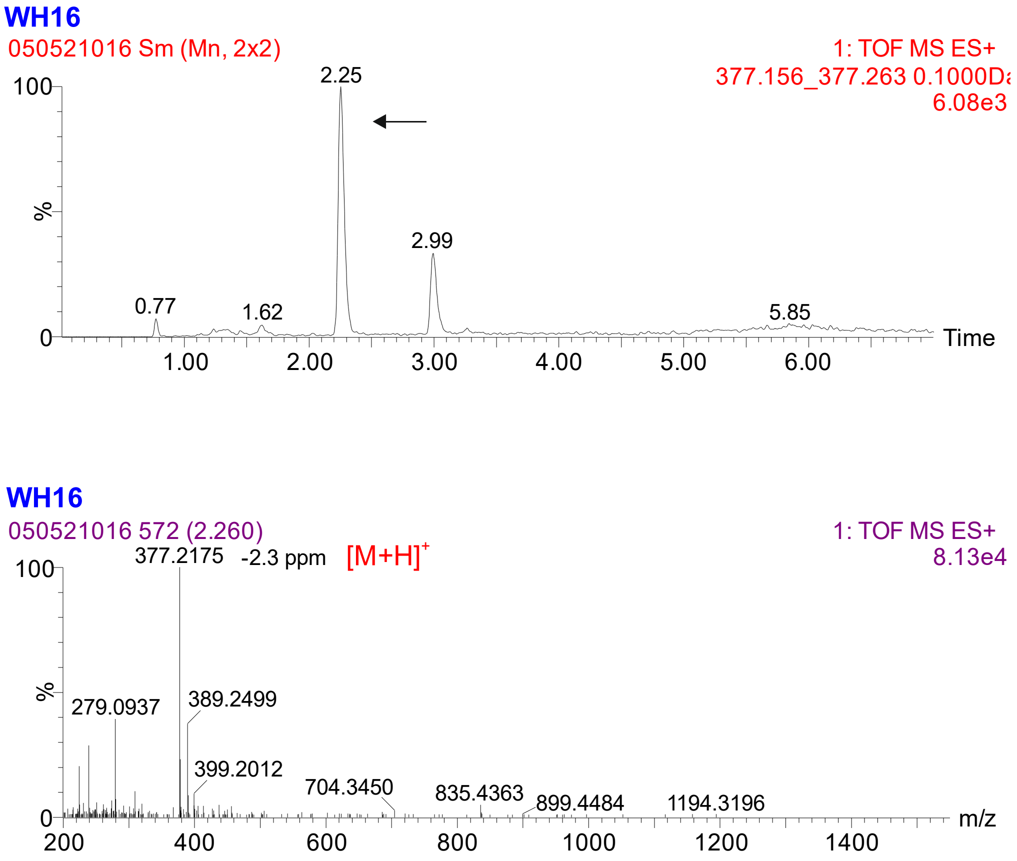
WH16
050521016 Sm (Mn, 2x2)
1: TOF MS ES+
377.156_377.263 0.1000Da
6.08e3
2.25
2.99
0.77
1.62
5.85
100
%
0
Time
1.00
2.00
3.00
4.00
5.00
6.00
WH16
050521016 572 (2.260)
1: TOF MS ES+
8.13e4
377.2175 -2.3 ppm [M+H]+
279.0937
389.2499
399.2012
704.3450
835.4363
899.4484
1194.3196
100
%
0
m/z
200
400
600
800
1000
1200
1400

Figure S27: LC HRMS of the biochemical reaction of the substrate *Z*-L-arginine-OH (**19**) and AutF in the presence of DMAPP. A) LCMS of the unprocessed substrate, B) LCMS of the prenylated product.

A

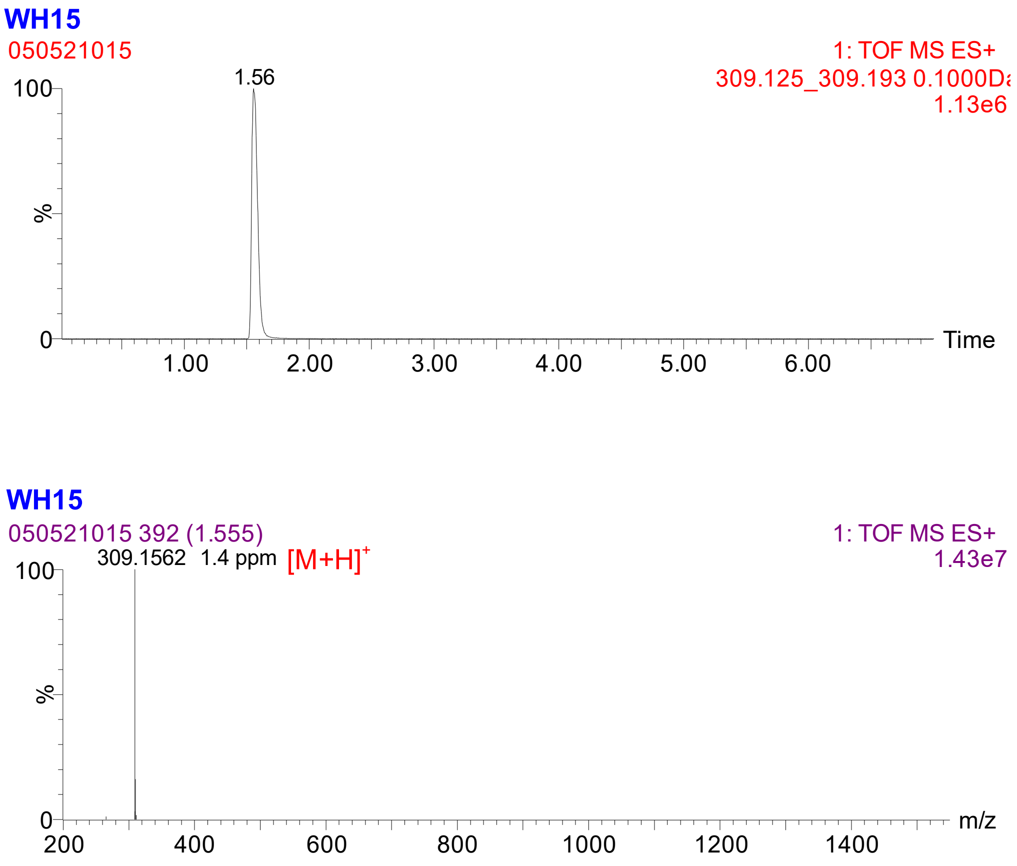
WH15
050521015
1: TOF MS ES+
309.125_309.193 0.1000Da
1.13e6
1.56
100
%
0
Time
1.00
2.00
3.00
4.00
5.00
6.00
WH15
050521015 392 (1.555)
1: TOF MS ES+
1.43e7
309.1562 1.4 ppm [M+H]+
100
%
0
m/z
200
400
600
800
1000
1200
1400

B

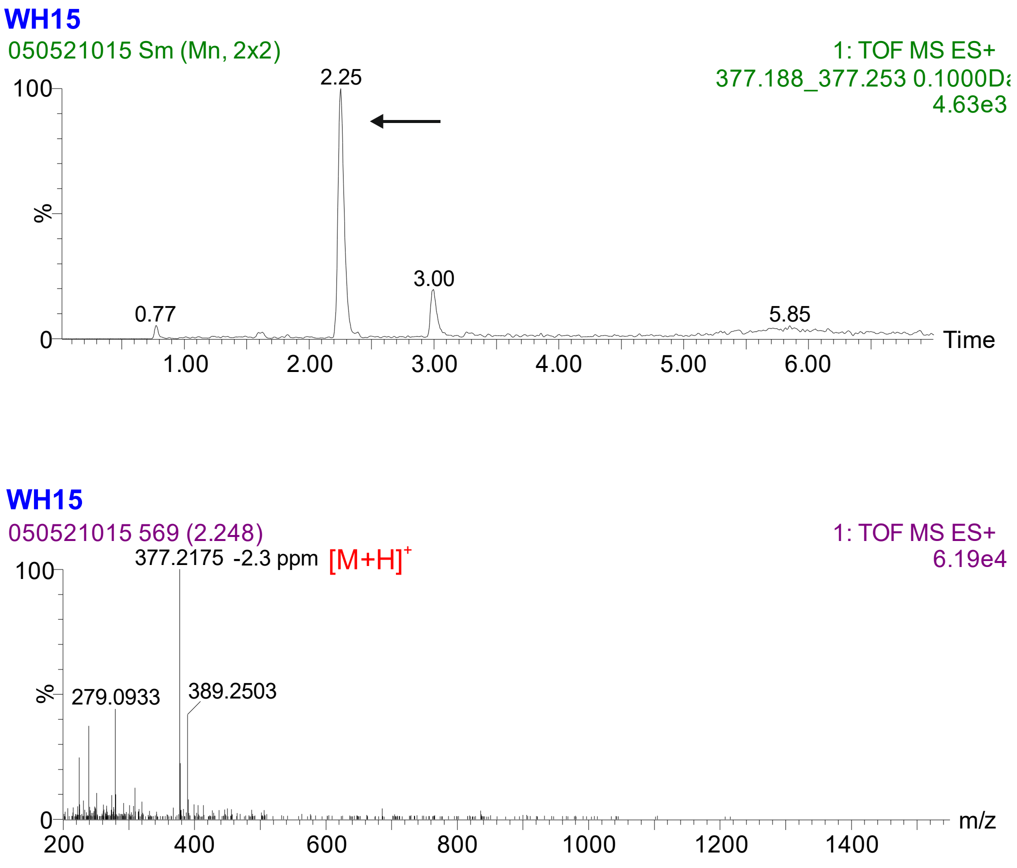
WH15
050521015 Sm (Mn, 2x2)
1: TOF MS ES+
377.188_377.253 0.1000Da
4.63e3
2.25
3.00
0.77
5.85
100
%
0
Time
1.00
2.00
3.00
4.00
5.00
6.00
WH15
050521015 569 (2.248)
1: TOF MS ES+
6.19e4
377.2175 -2.3 ppm [M+H]+
279.0933
389.2503
100
%
0
m/z
200
400
600
800
1000
1200
1400

Figure S28: LC HRMS of the biochemical reaction of the substrate *Z*-D-arginine-OH (**20**) and AutF in the presence of DMAPP. A) LCMS of the unprocessed substrate, B) LCMS of the prenylated product.

A

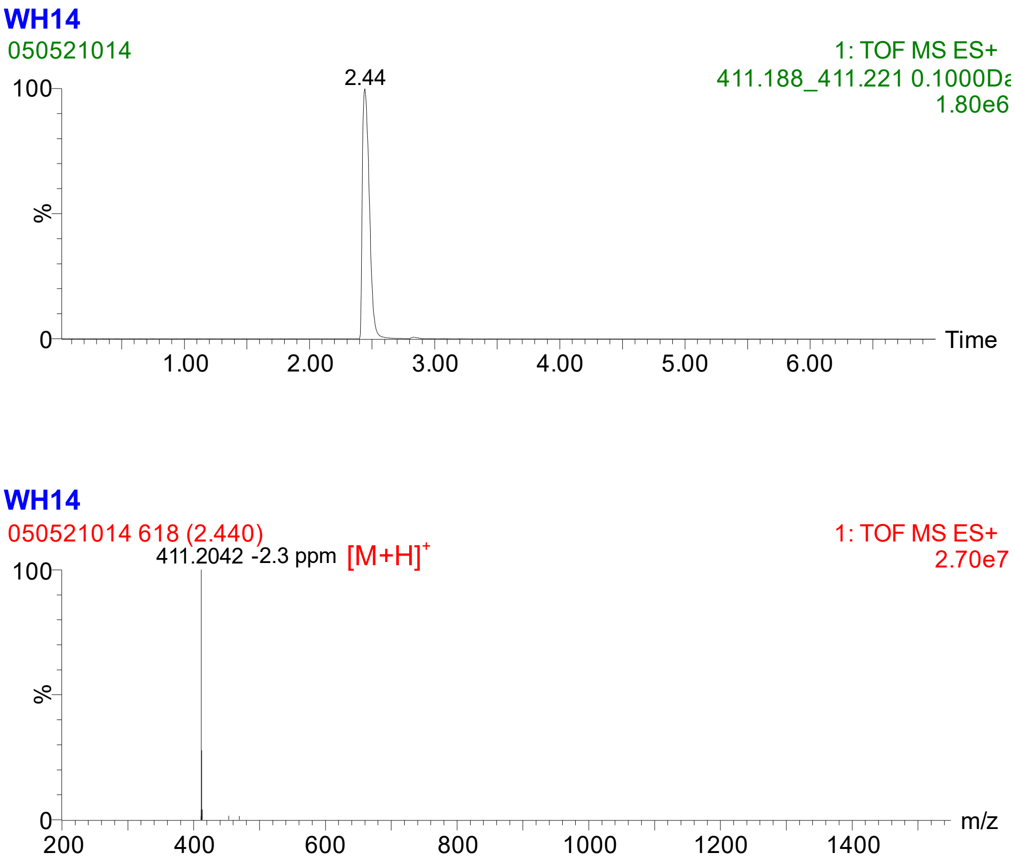
WH14
050521014
1: TOF MS ES+
411.188_411.221 0.1000Da
1.80e6
2.44
100
%
0
Time
1.00
2.00
3.00
4.00
5.00
6.00
WH14
050521014 618 (2.440)
1: TOF MS ES+
2.70e7
411.2042 -2.3 ppm [M+H]+
100
%
0
m/z
200
400
600
800
1000
1200
1400

B

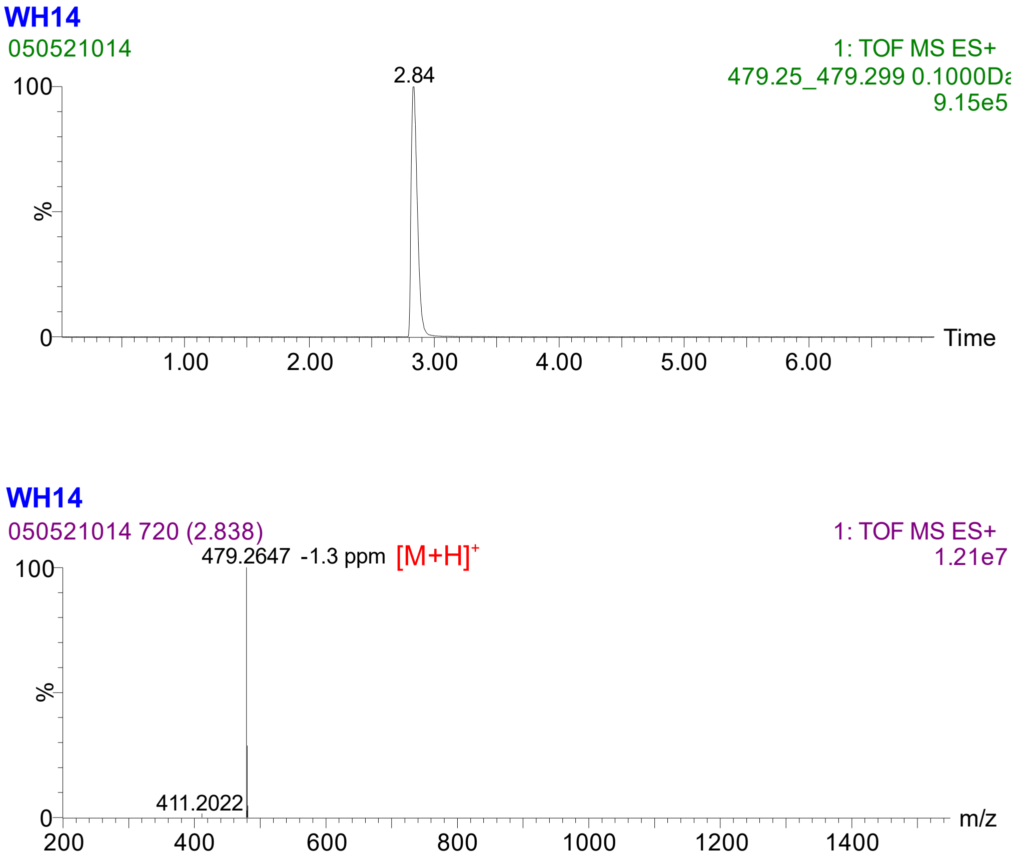
WH14
050521014
1: TOF MS ES+
479.25_479.299 0.1000Da
9.15e5
2.84
100
%
0
Time
1.00
2.00
3.00
4.00
5.00
6.00
WH14
050521014 720 (2.838)
1: TOF MS ES+
1.21e7
479.2647 -1.3 ppm [M+H]+
411.2022
100
%
0
m/z
200
400
600
800
1000
1200
1400

Figure S29: LC HRMS of the biochemical reaction of the substrate Fmoc-Homoarginine-OH (**21**) and AutF in the presence of DMAPP. A) LCMS of the unprocessed substrate, B) LCMS of the prenylated product.

A

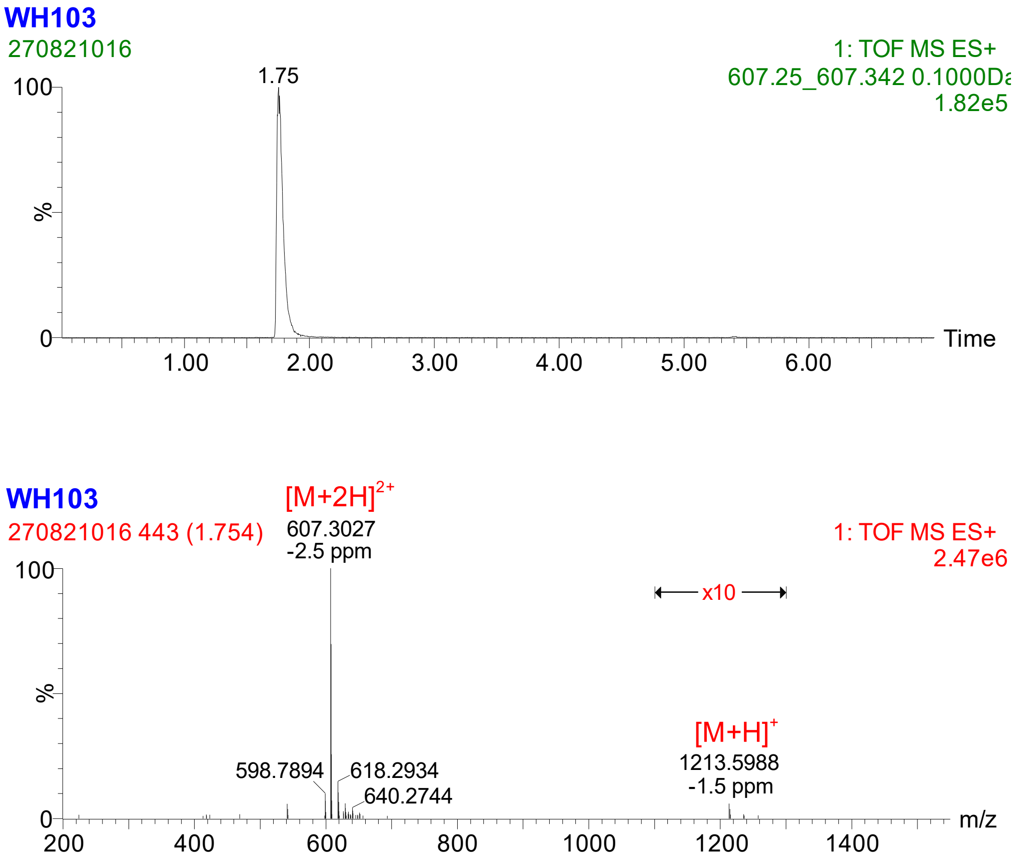
WH103
270821016
1: TOF MS ES+
607.25_607.342 0.1000Da
1.82e5
1.75
100
%
0
1.00
2.00
3.00
4.00
5.00
6.00
Time
WH103
270821016 443 (1.754)
[M+2H]2+
607.3027
-2.5 ppm
1: TOF MS ES+
2.47e6
x10
[M+H]+
1213.5988
-1.5 ppm
598.7894
618.2934
640.2744
100
%
0
200
400
600
800
1000
1200
1400
m/z

B

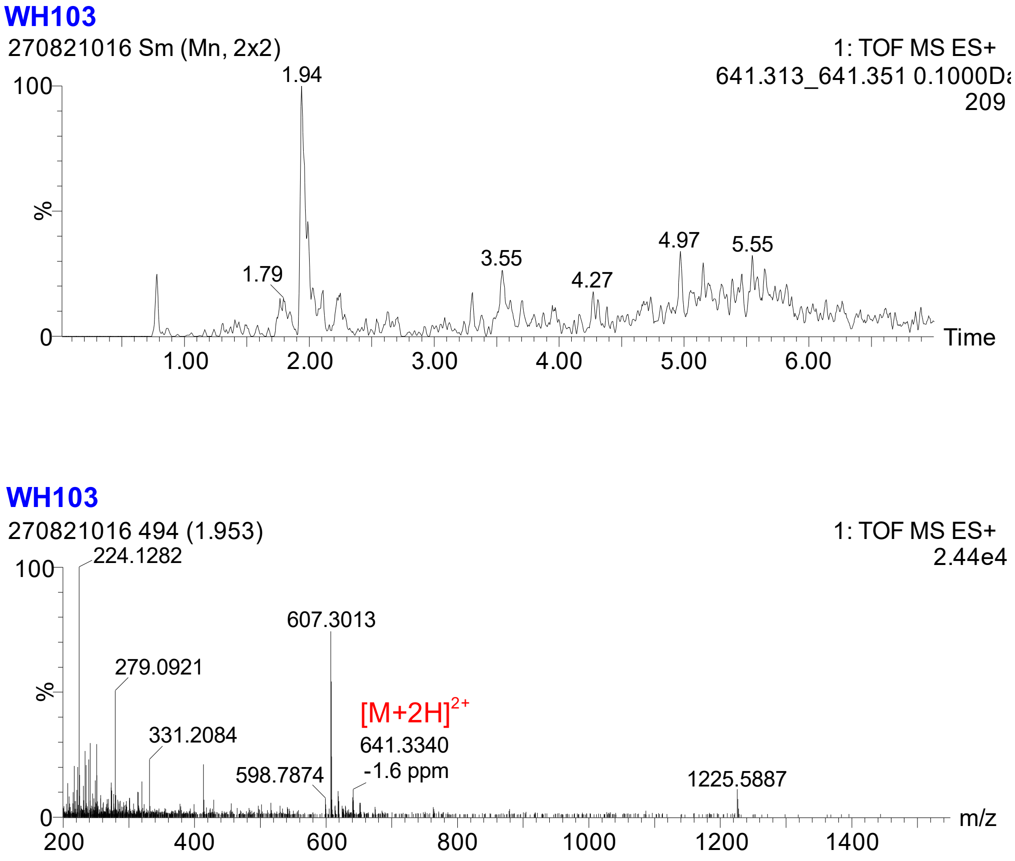
WH103
270821016 Sm (Mn, 2x2)
1: TOF MS ES+
641.313_641.351 0.1000Da
209
1.94
1.79
3.55
4.27
4.97
5.55
100
%
0
1.00
2.00
3.00
4.00
5.00
6.00
Time
WH103
270821016 494 (1.953)
1: TOF MS ES+
2.44e4
224.1282
607.3013
279.0921
331.2084
598.7874
[M+2H]2+
641.3340
-1.6 ppm
1225.5887
100
%
0
200
400
600
800
1000
1200
1400
m/z

Figure S30: LC HRMS of the biochemical reaction of peptide H-FXFDLGPAYD-NH_2 (**22**) where X is L-homoarginine and AutF in the presence of DMAPP. A) LCMS of the unprocessed substrate, B) LCMS of the prenylated product.

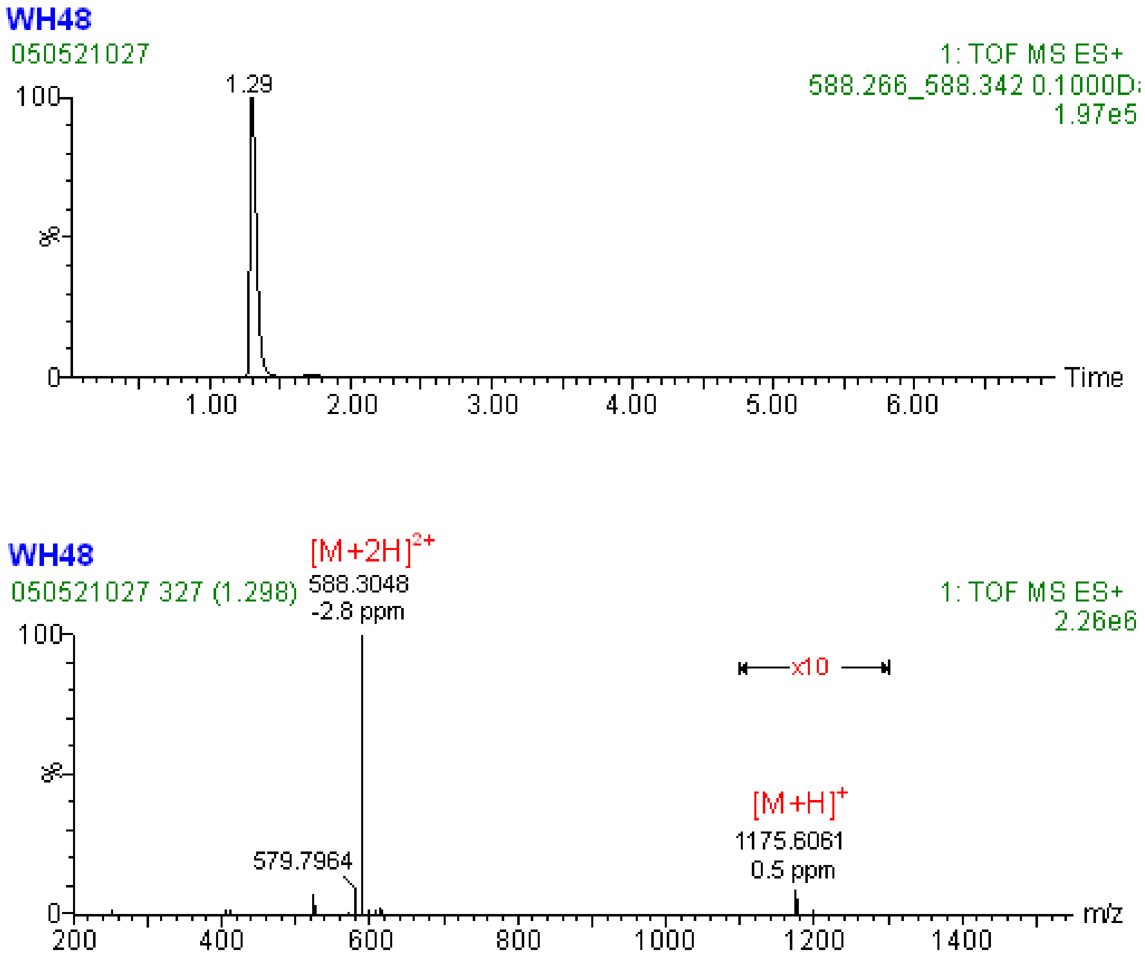

Figure S31: LC HRMS of the unprocessed substrate in the reaction mixture of H-QYLDEKLPNG-NH_2 (**23**) and AutF in the presence of DMAPP.

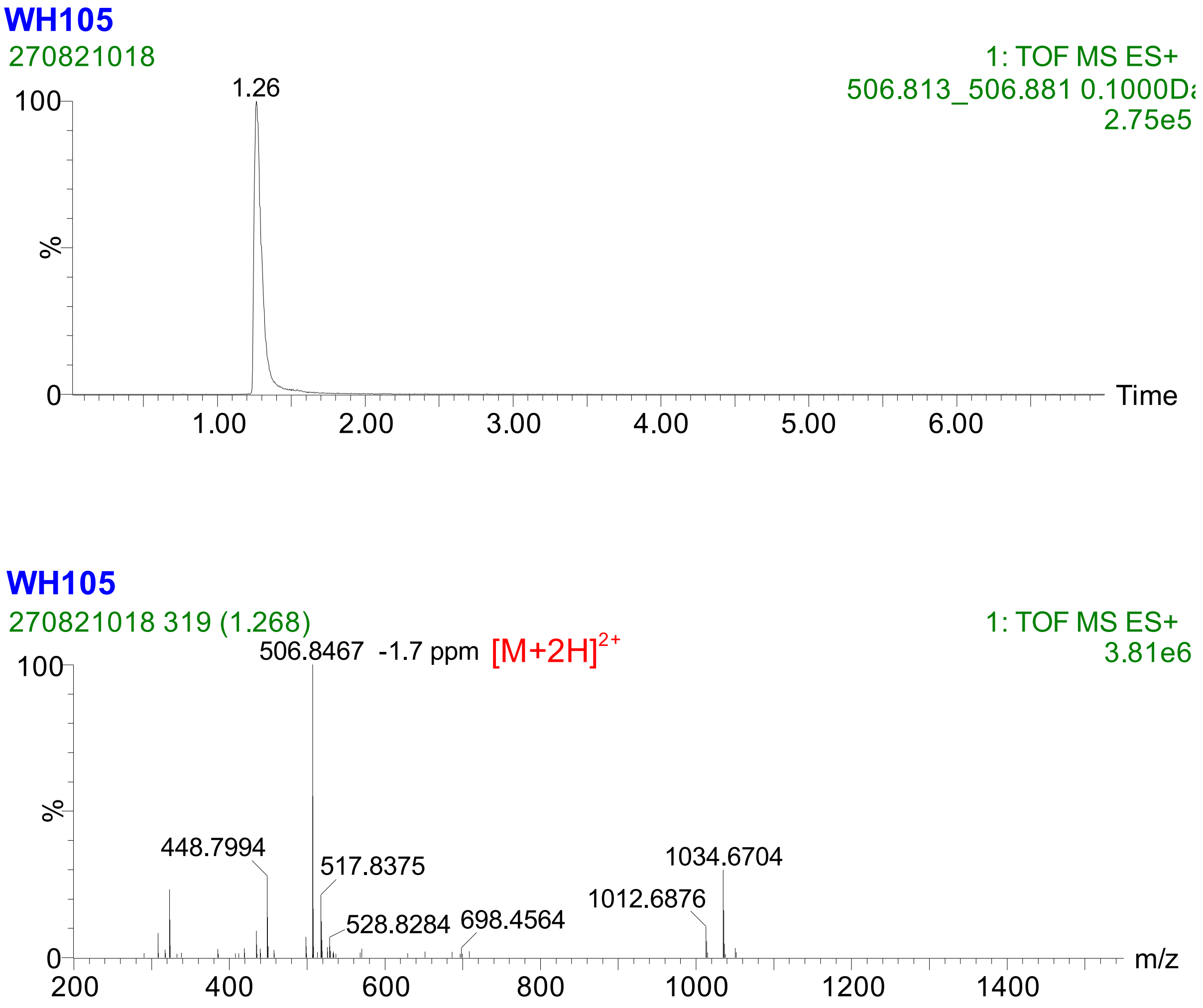

Figure S32: LC HRMS of the unprocessed substrate in the reaction mixture of H-VVKGALKSLV-NH_2 (**24**) and AutF in the presence of DMAPP.

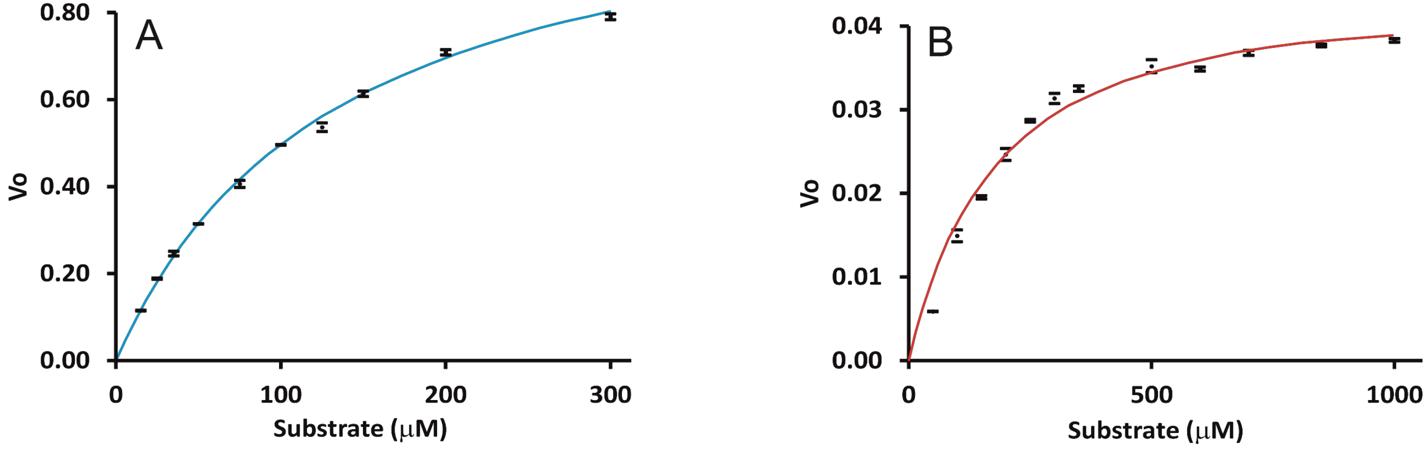

Figure S33: Michaelis-Menton kinetics for AutF with A) H-FRFDLGPAYD-NH_2 (**10**) and B) Fmoc-L-homoarginine-OH (**21**). Experiments were done in duplicates and error bars indicate the SE of the mean.

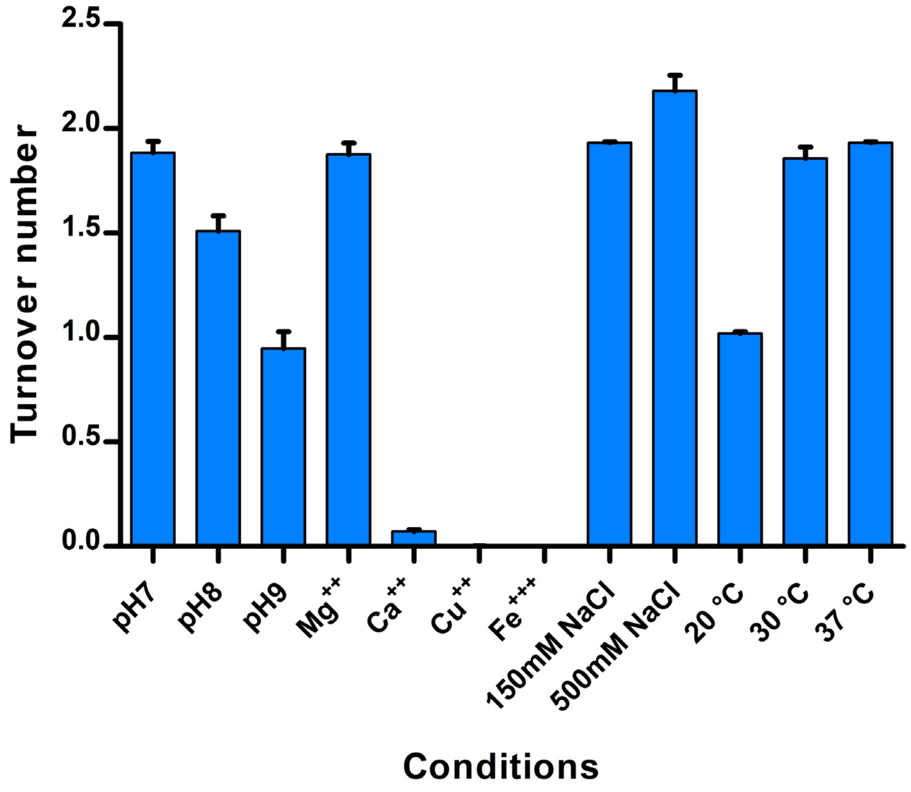

Figure S34: Turnover number (K_{cat}) of AutF and Fmoc-homoarginine (21) in different reaction conditions (pH, metals, salt concentrations and temperatures). Experiments were done in duplicates and error bars indicate the SE of the mean.

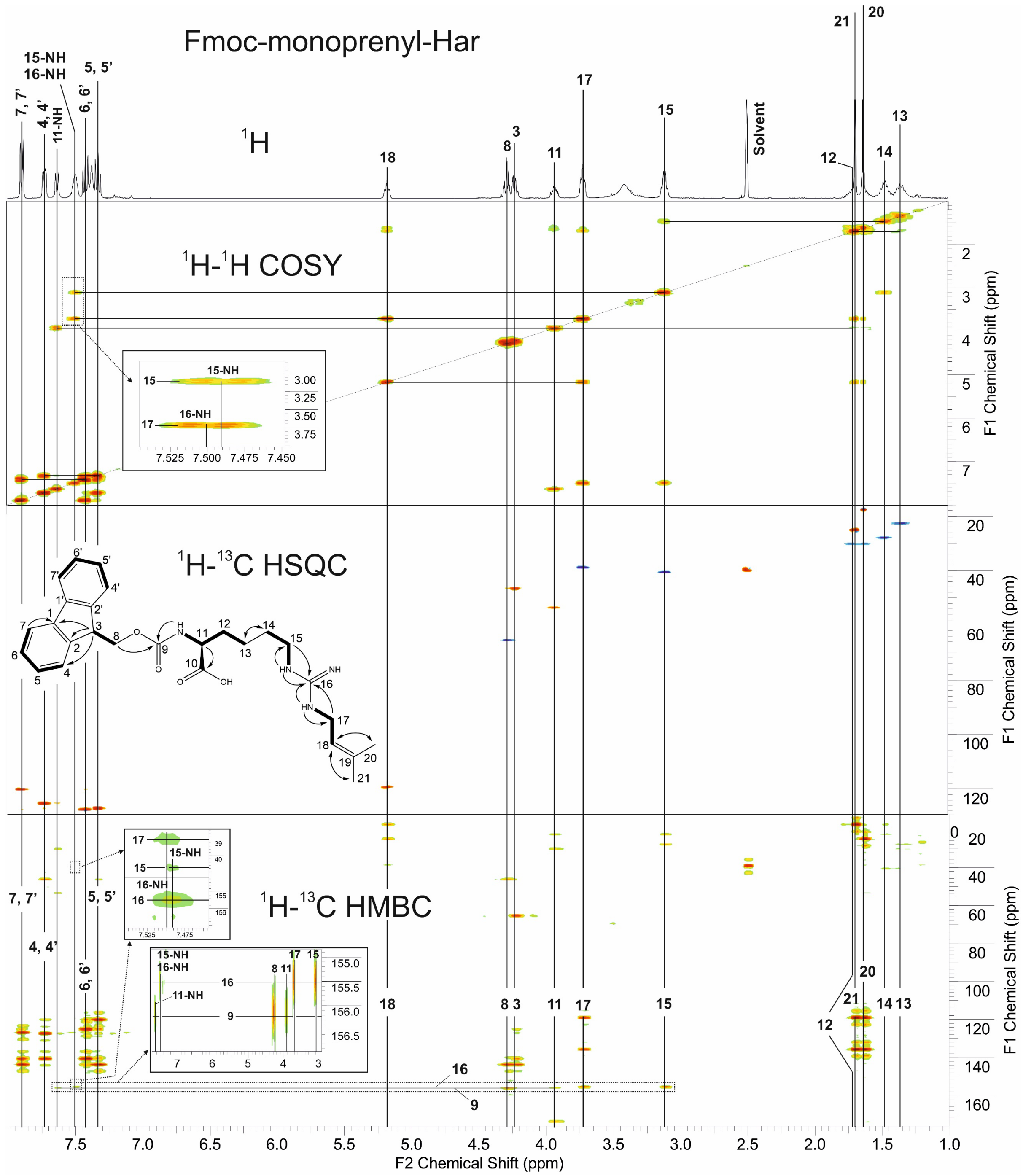

Figure S35a: ^{1}H, ^{1}H-^{1}H COSY, ^{1}H-^{13}C HSQC and ^{1}H-^{13}C HMBC NMR spectra of the N^{ω}-Fmoc-homoarginine in DMSO-d_6. The chemical structure shows the observed 1H-1H-COSY correlations as thick bonds and 1H-13C HMBC correlations as arrows. ^{1}H-^{1}H COSY and HMBC correlations between 16, 16-NH and 17 confirmed the position of prenylation. This is in addition to the fact that there is 11-NH and 15-NH protons which cannot be present if prenyl is connected to these positions.

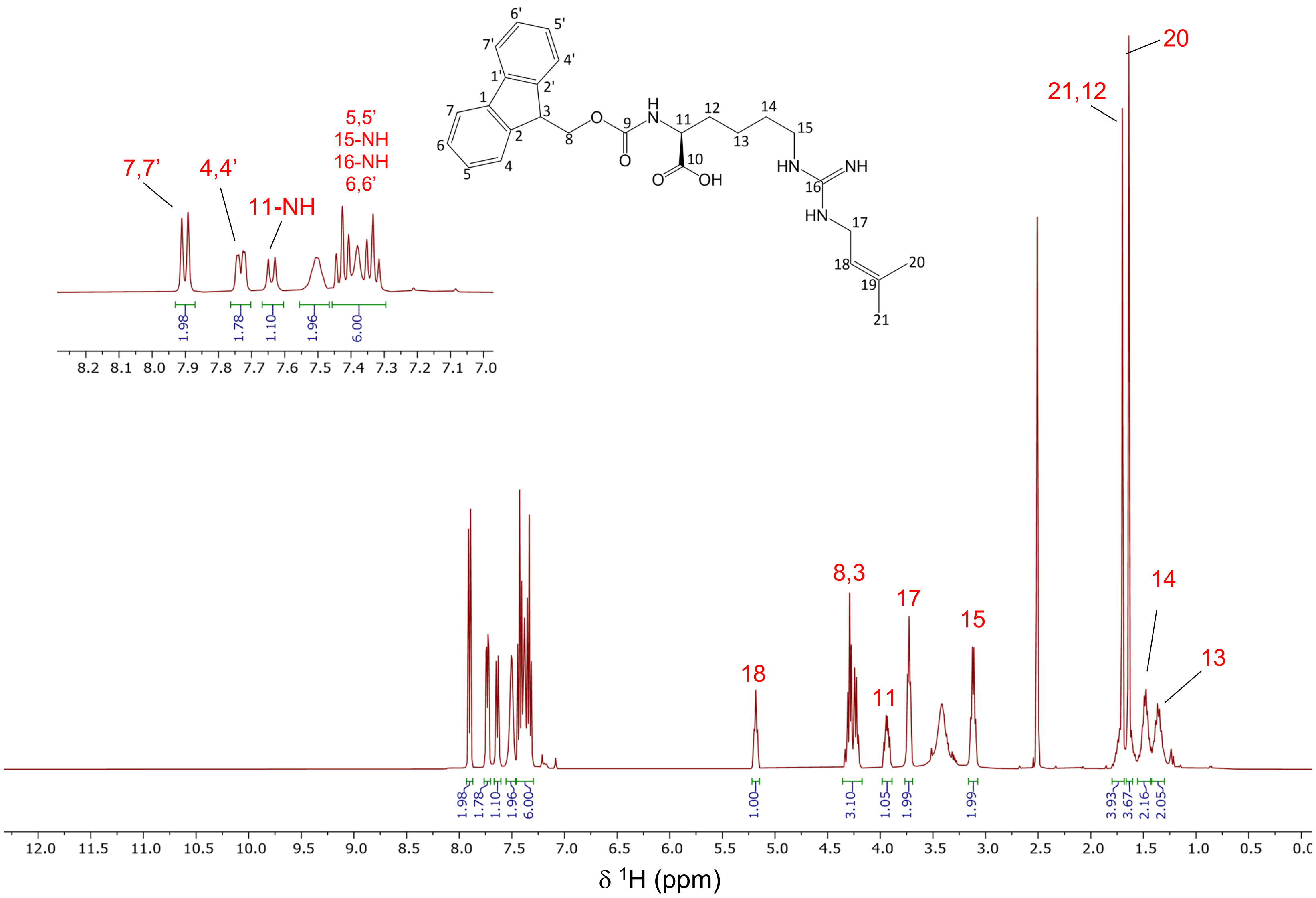

Figure S35b: ^{1}H NMR (400 MHz) spectrum of the *N*$^{\omega}$-monoprenylated Fmoc-homoarginine in DMSO-*d*$_6$.

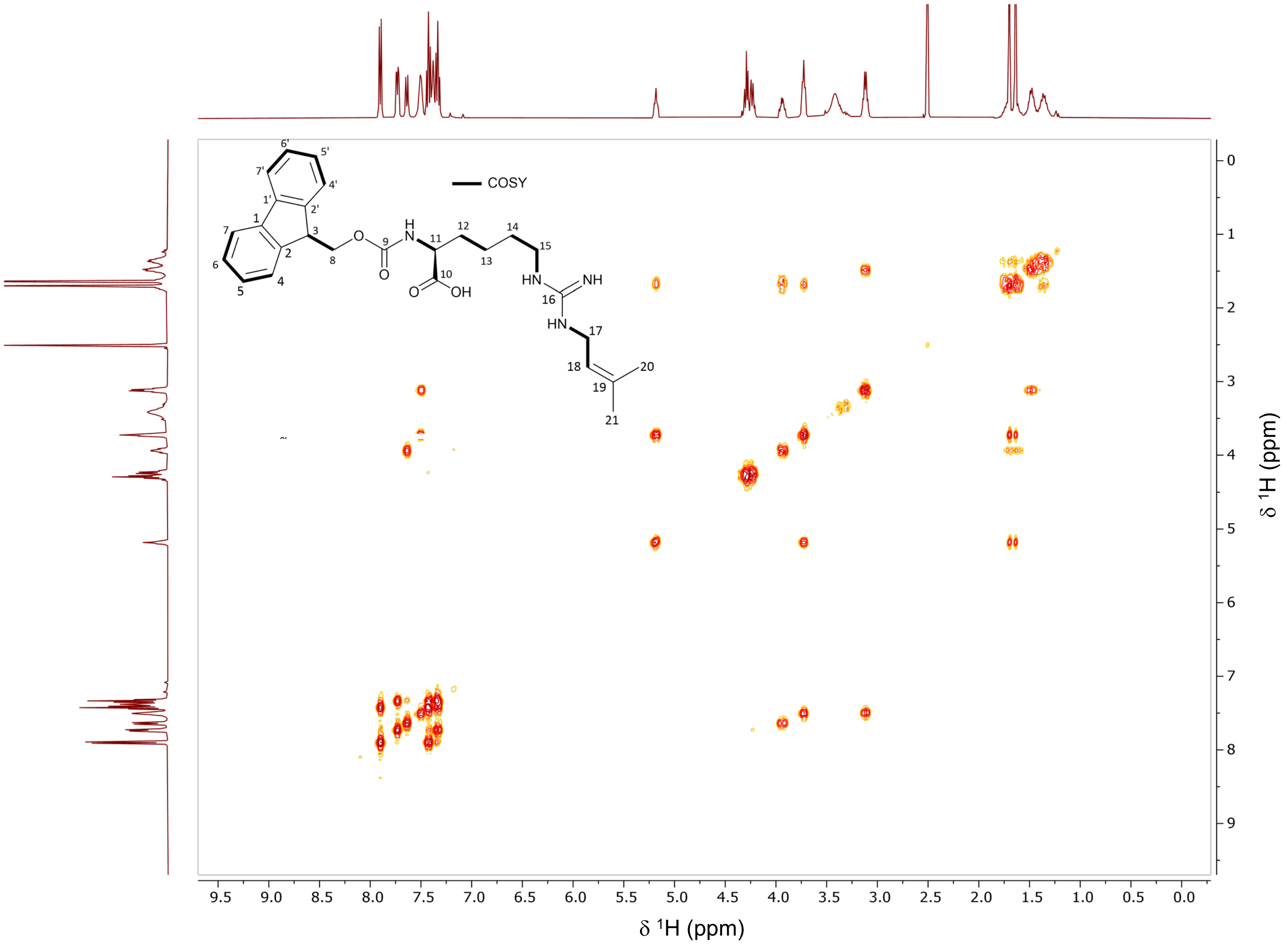

Figure S35c: ^{1}H-^{1}H COSY NMR (400 MHz) full spectrum of the N^{ω}-monoprenylated Fmoc-homoarginine in DMSO-d_6.

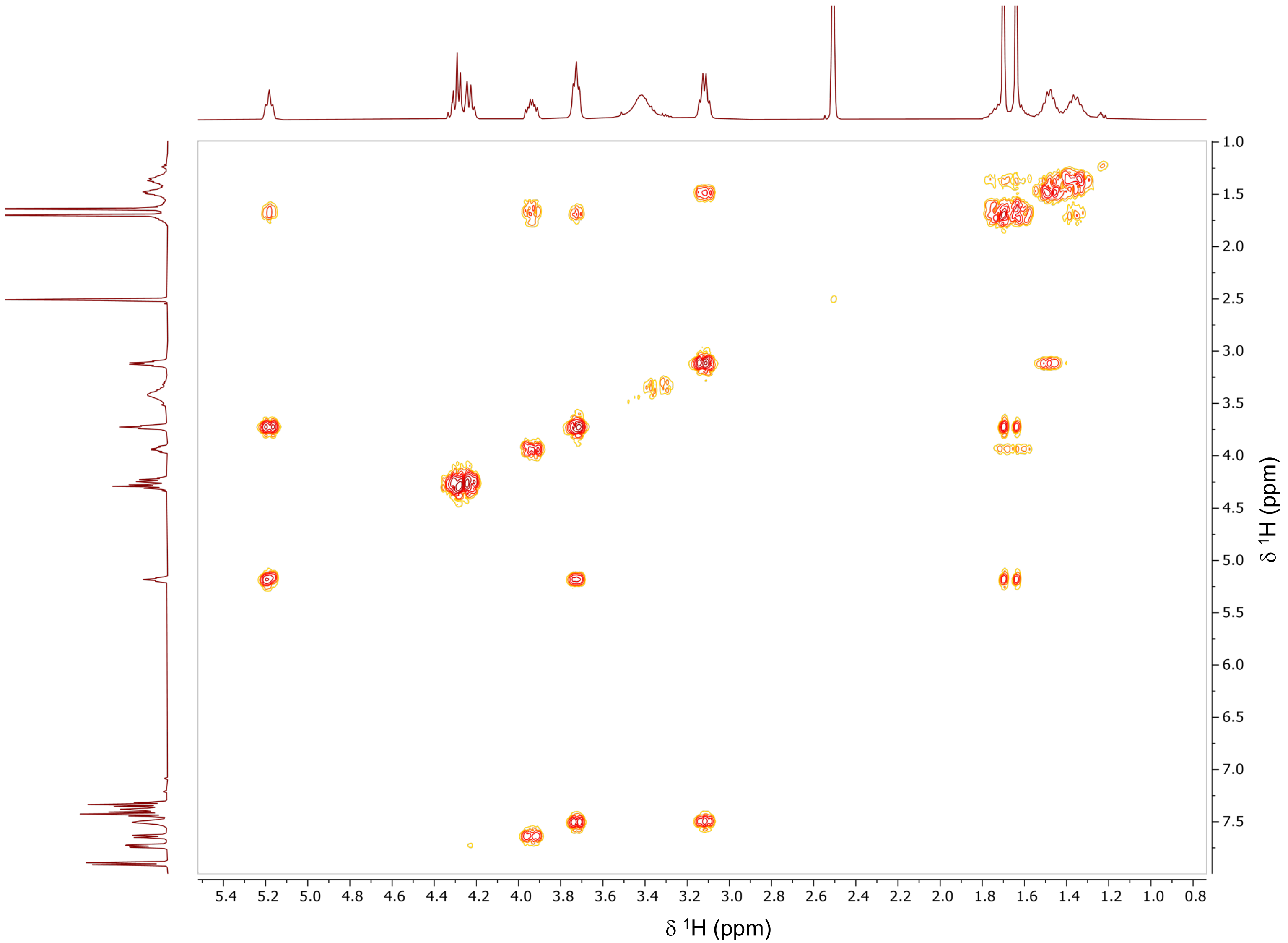

Figure S35d: ^{1}H-^{1}H COSY NMR (400 MHz) spectrum of the *N*$^{\omega}$-monoprenylated Fmoc-homoarginine in DMSO-d_6.

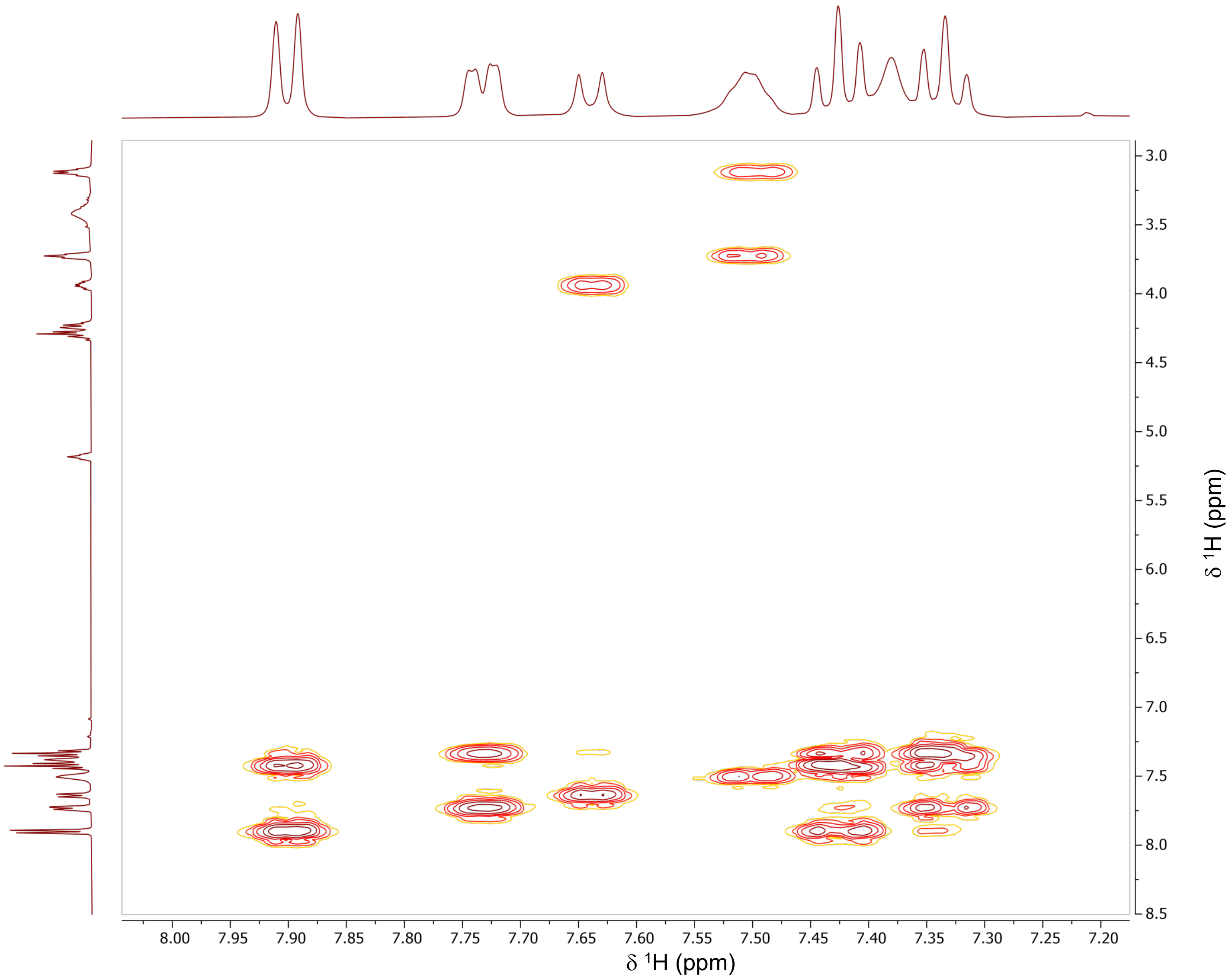

Figure S35e: ^{1}H-^{1}H COSY NMR (400 MHz) spectrum of the N^{ω}-monoprenylated Fmoc-homoarginine in DMSO-d_6.

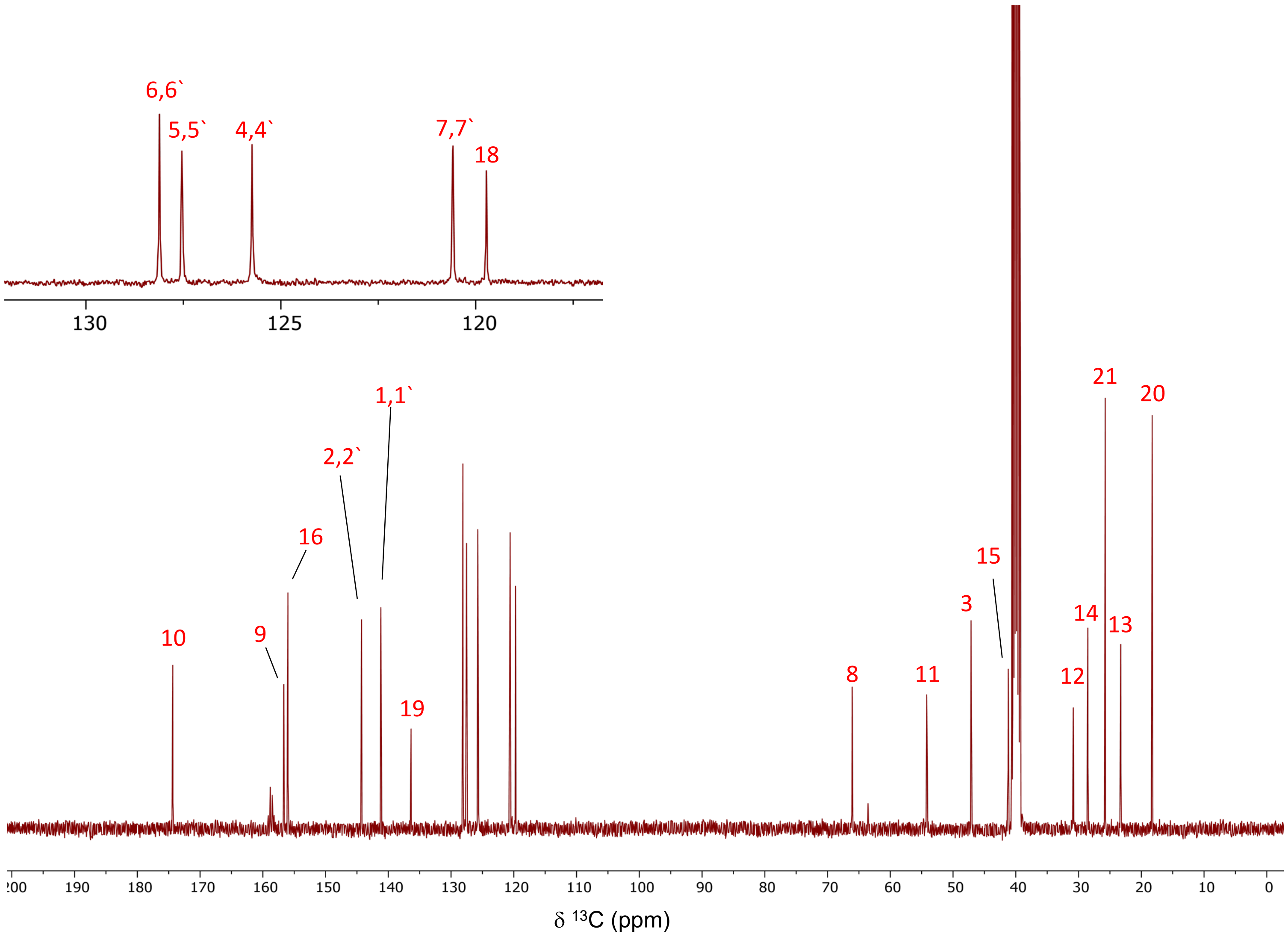

Figure S35f: ^{13}C NMR (100 MHz) spectrum of the *N*$^{\omega}$-monoprenylated Fmoc-homoarginine in DMSO-d_6.

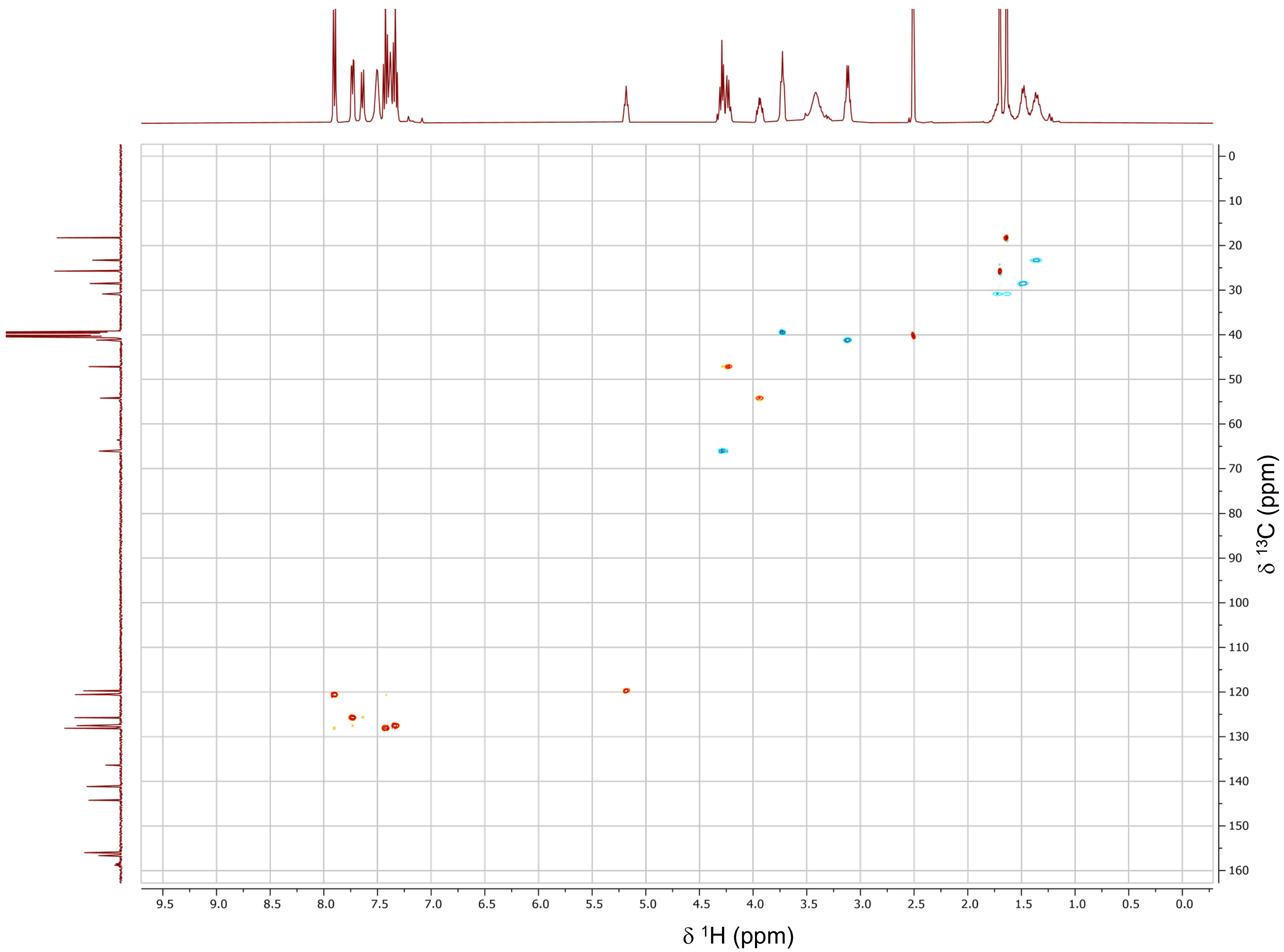

Figure S35g: HSQC NMR (400 MHz) spectrum of the N^{ω}-monoprenylated Fmoc-homoarginine in DMSO-d_6.

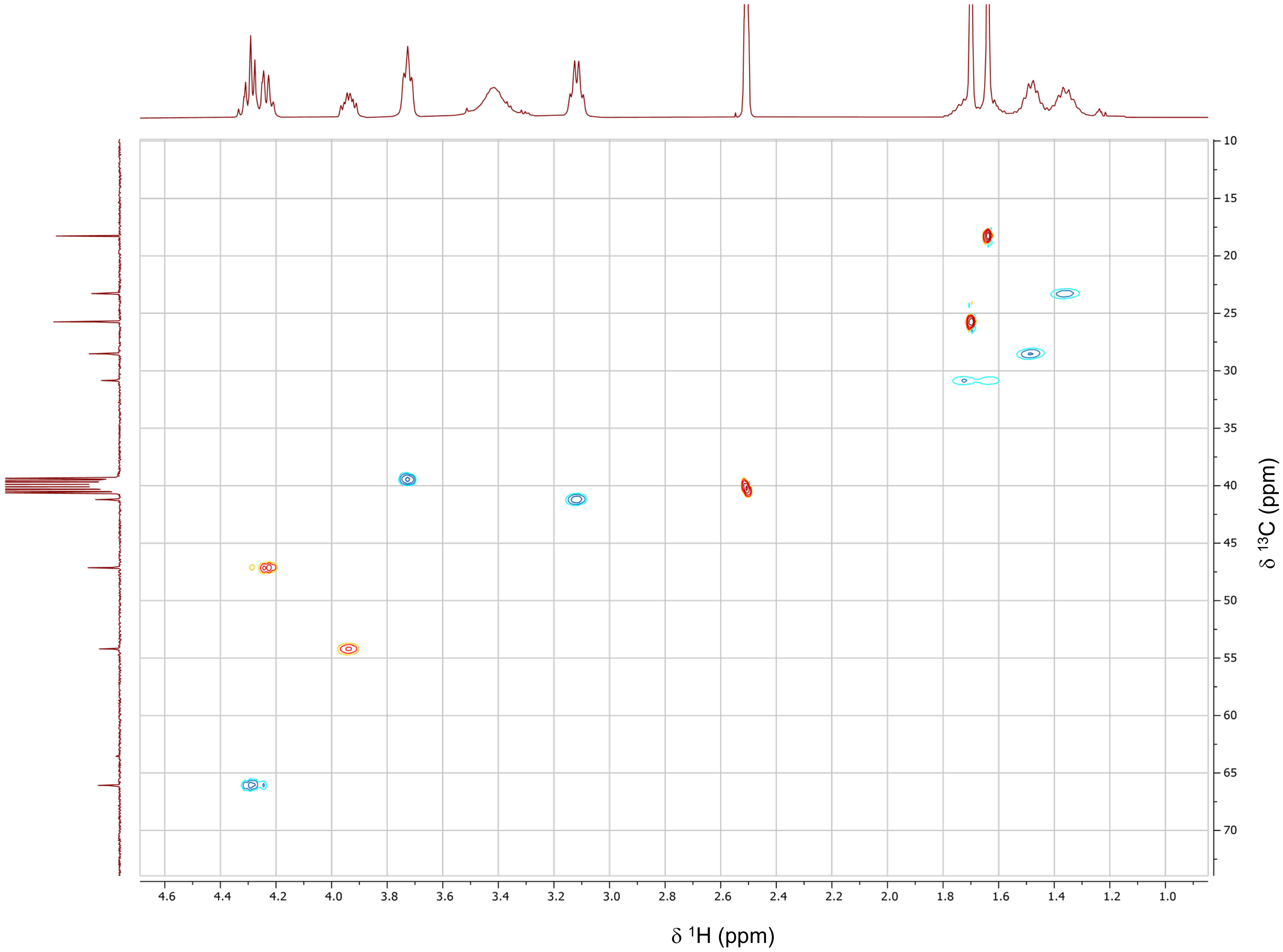

Figure S35h: HSQC NMR (400 MHz) spectrum of the N^{ω}-monoprenylated Fmoc-homoarginine in DMSO-d_6.

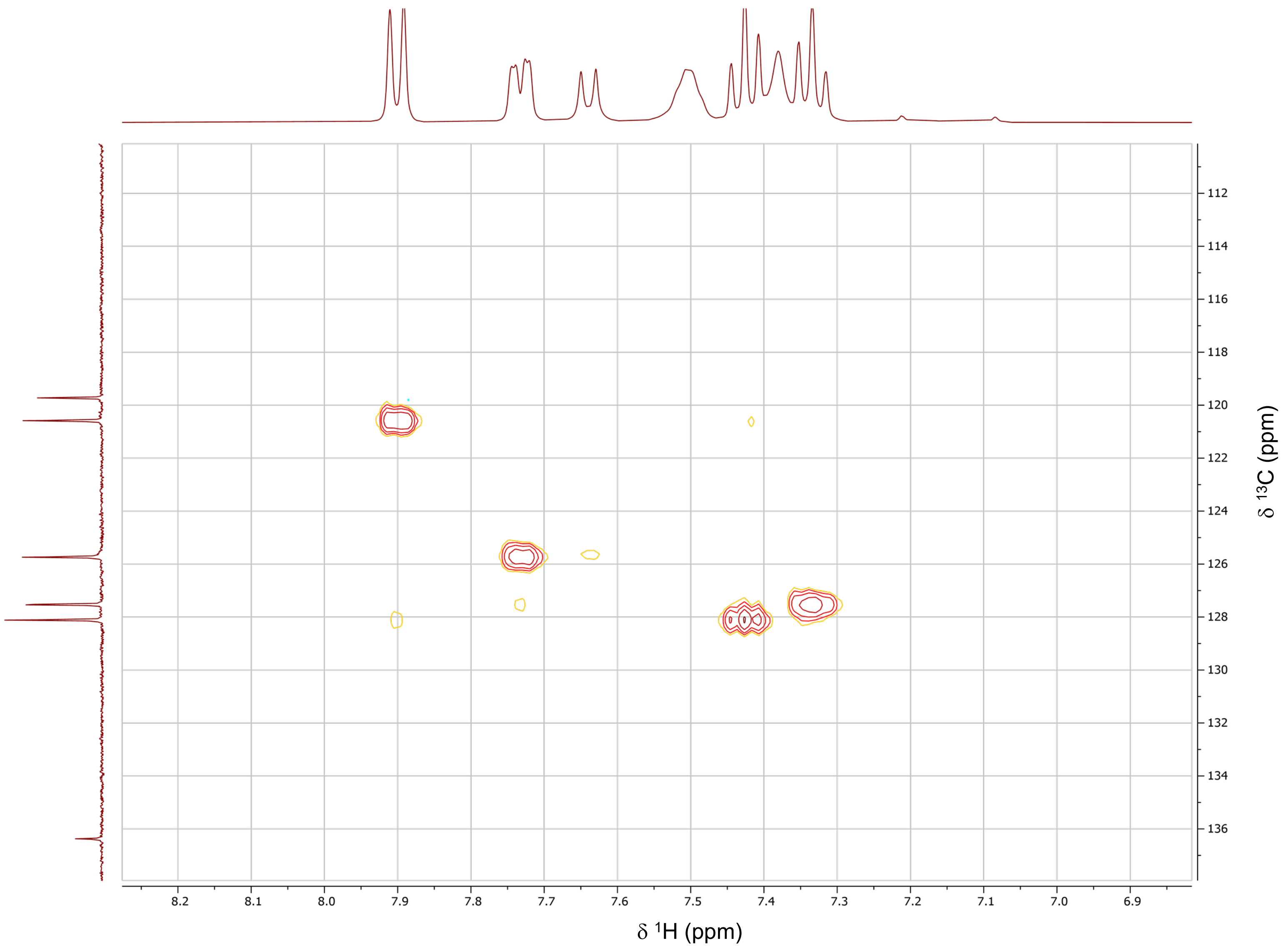

Figure S35i: HSQC NMR (400 MHz) spectrum of the N^{ω}-monoprenylated Fmoc-homoarginine in DMSO-d_6.

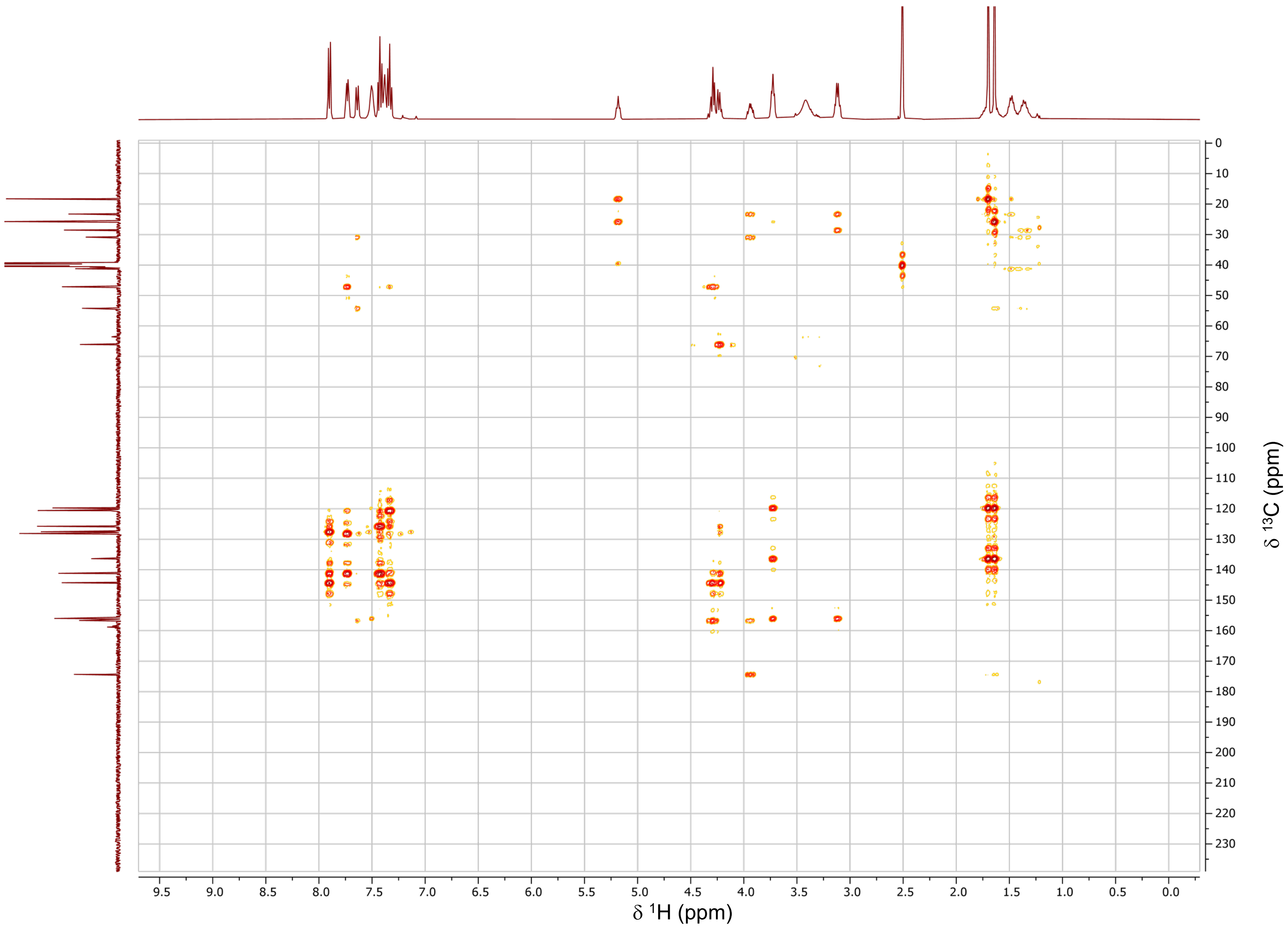

Figure S35j: HMBC NMR (400 MHz) spectrum of the N^{ω}-monoprenylated Fmoc-homoarginine in DMSO-d_6.

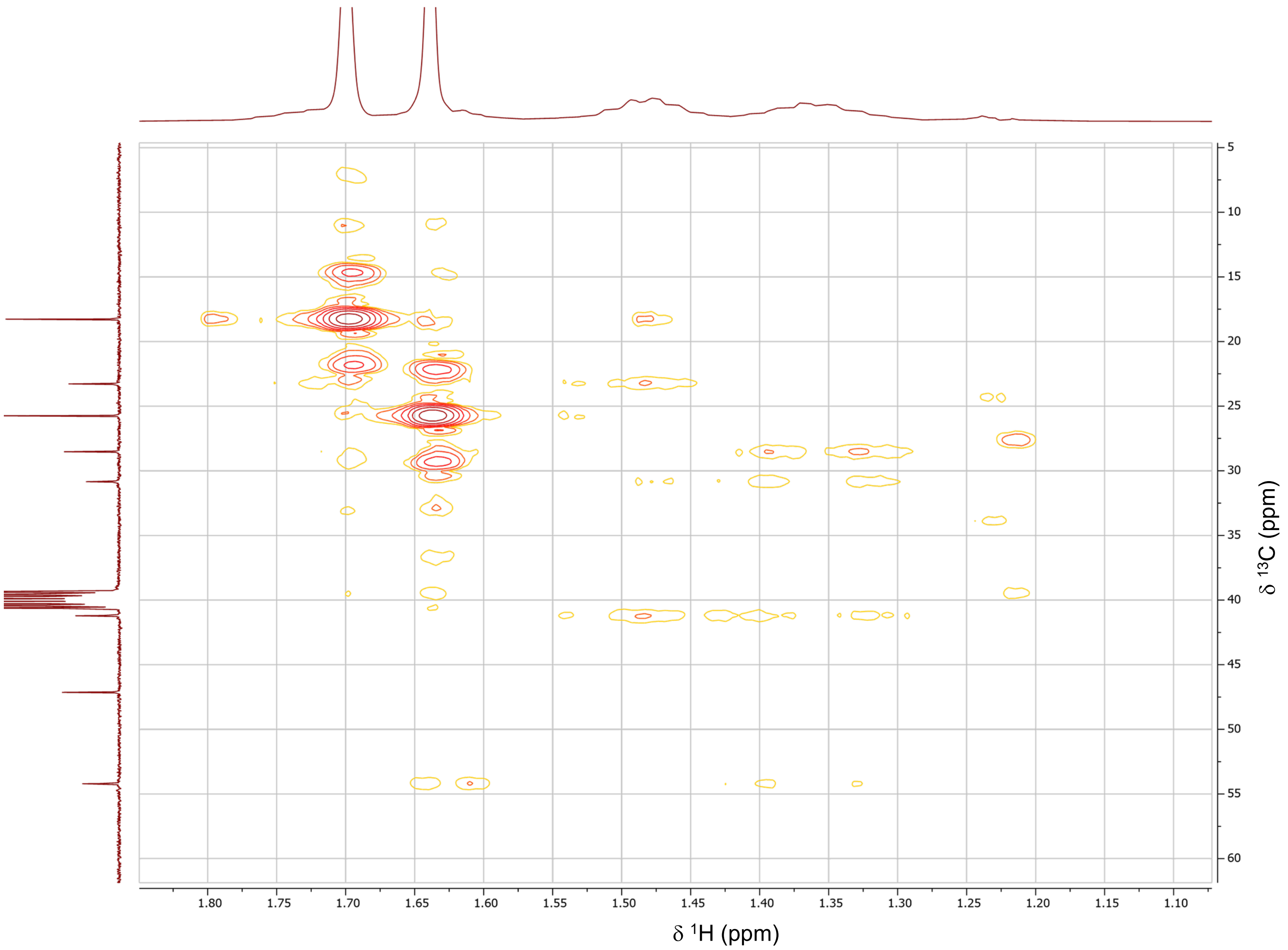

Figure S35k: HMBC NMR (400 MHz) spectrum of the N^{ω}-monoprenylated Fmoc-homoarginine in DMSO-d_6.

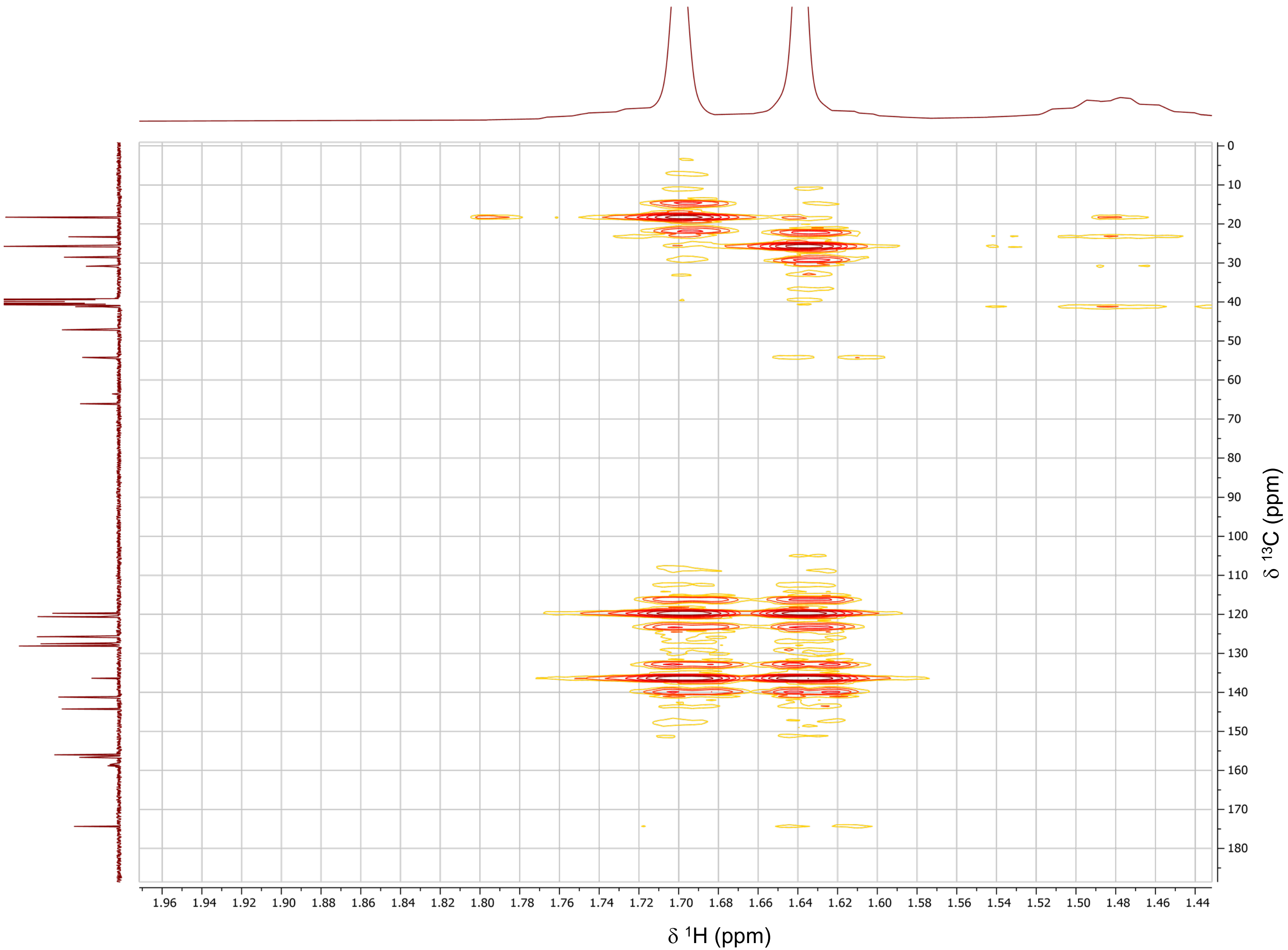

Figure S35l: HMBC NMR (400 MHz) spectrum of the N^{ω}-monoprenylated Fmoc-homoarginine in DMSO-d_6.

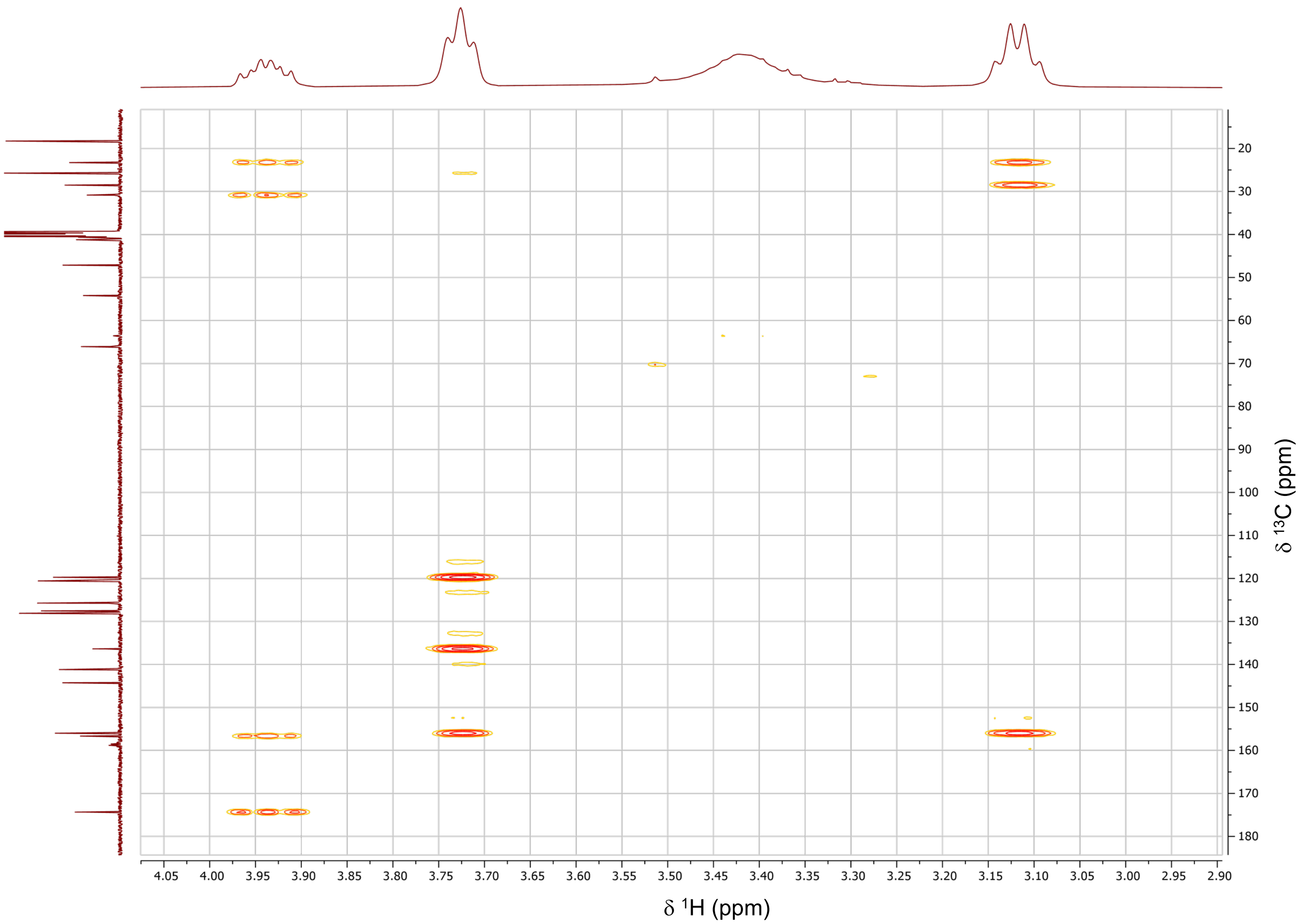

Figure S35m: HMBC NMR (400 MHz) spectrum of the N^{ω}-monoprenylated Fmoc-homoarginine in DMSO-d_6.

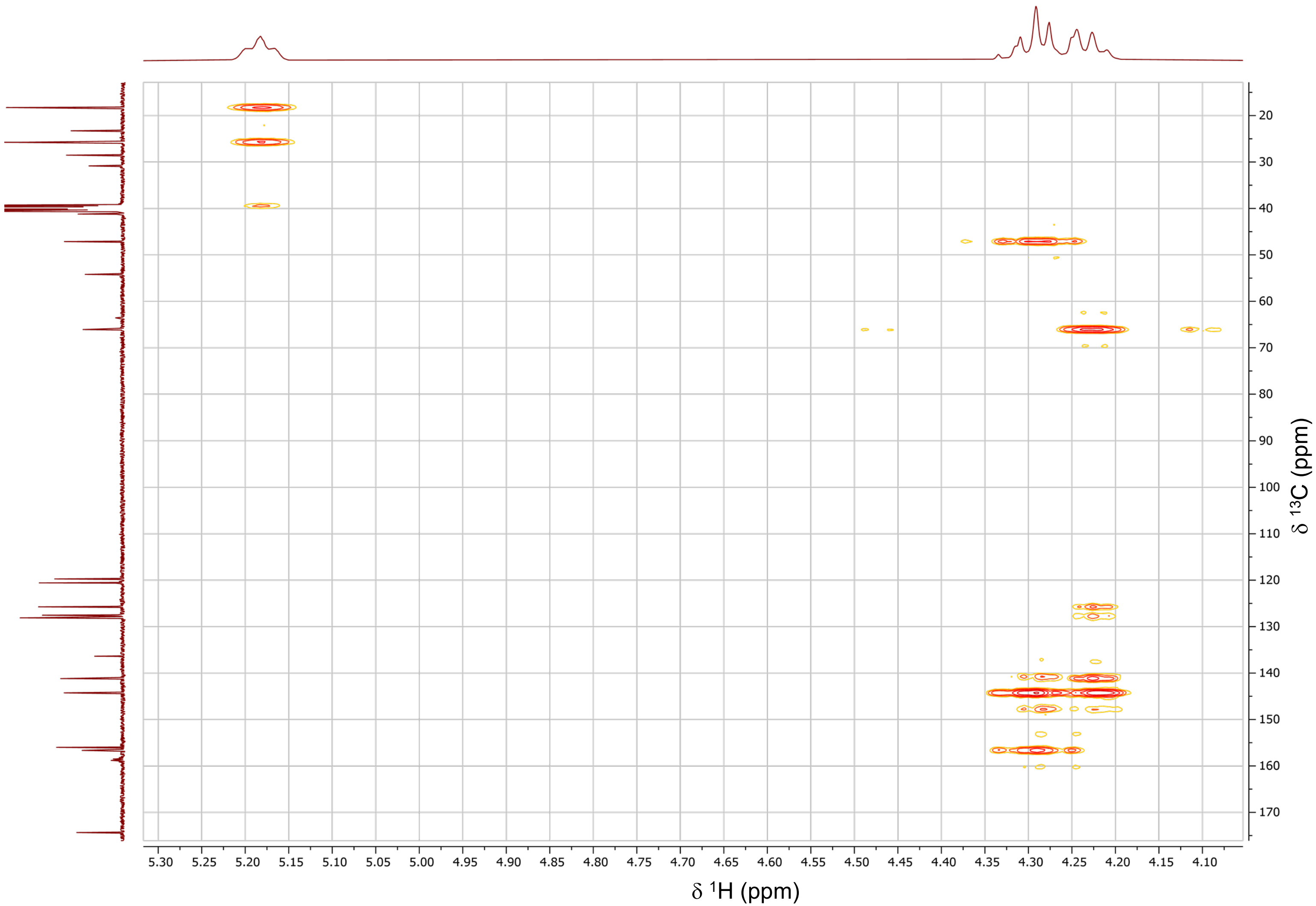

Figure S35n: HMBC NMR (400 MHz) spectrum of the N^{ω}-monoprenylated Fmoc-homoarginine in DMSO-d_6.

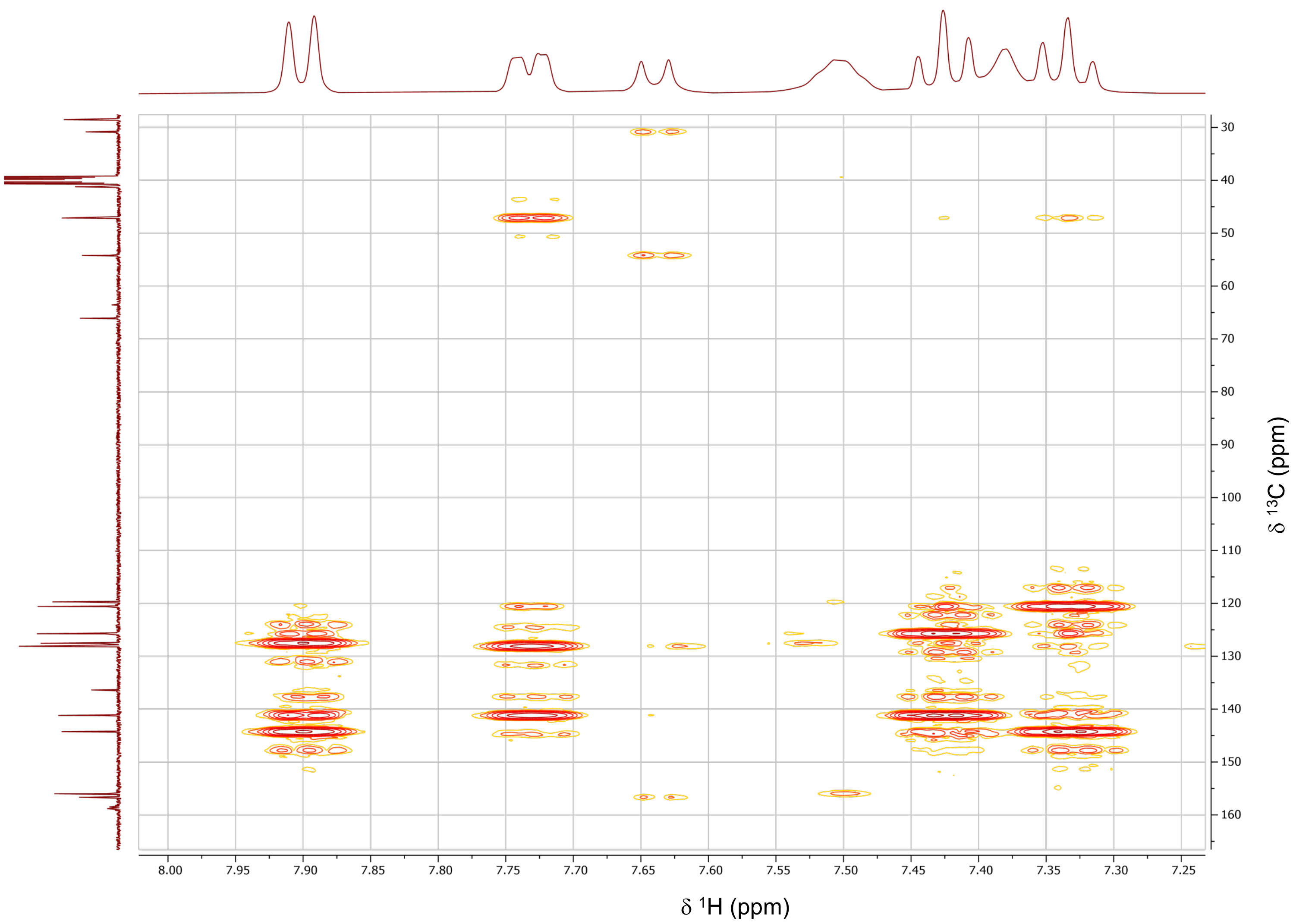

Figure S35o: HMBC NMR (400 MHz) spectrum of the N^{ω}-monoprenylated Fmoc-homoarginine in DMSO-d_6.

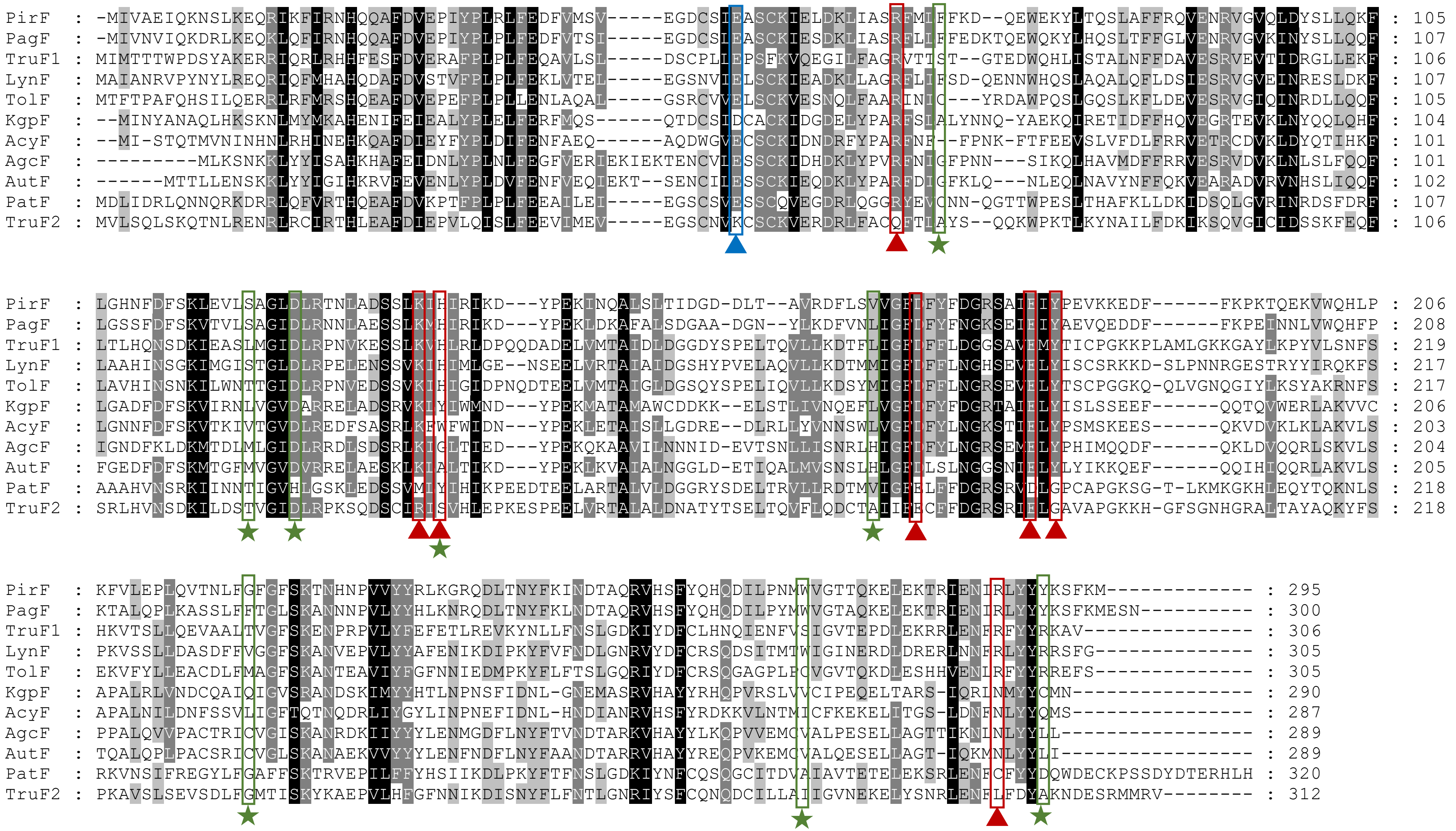

PirF : -MIVAEIQKNSLKEQRIKFIRNHQQAFDVEPIYPLRLFEDFVMSV-----EGDCSIEASCKIELDKLIASRFMIFFKD--QEWEKYLTQSLAFFRQVENRVGVQLDYSLLQKF : 105
PagF : -MIVNVIQKDRLKEQKLQFIRNHQQAFDVEPIYPLPLFEDFVTSI-----EGDCSIEASCKIESDKLIASRFLIFFEDKTQEWQKYLHQSLTFFGLVENRVGVKINYSLLQQF : 107
TruF1 : MIMTTTWPDSYAKERRIQRLRHHFESFDVERAFPLPLFEQAVLSL-----DSCPLLEPSFKVQEGILFAGRVTISST--GTEDWQHLISTALNFFDAVESRVEVTIDRGLLEKF : 106
LynF : MAIANRVPYNYLREQRIQFMHAHQDAFDVSTVFPLPLFEKLVTEL-----EGSNVIELSCKIEADKLLAGRFLIFSD-QENNWHQSLAQALQFLDSIESRVGVEINRESLDKF : 107
TolF : MTFTPAFQHSILQERRLRFMRSHQEAFDVEPEFPLPLLENLAQAL-----GSRCVVELSCKVESNQLFAARINIC---YRDAWPQSLGQSLKFLDEVESRVGIQINRDLLQQF : 105
KgpF : --MINYANAQLHKSKNLMYMKAHENIFEIEALYPLELFERFMQS------QTDCSIDCACKIDGDELYPARFSIALYNNQ-YAEKQIRETIDFFHQVEGRTEVKLNYQQLQHF : 104
AcyF : --MI-STQTMVNINHNLRHINEHKQAFDIEYFYPLDIFENFAEQ------AQDWGVECSCKIDNDRFYPARFNF-FPNK-FTFEEVSLVFDLFRRVETRCDVKLDYQTIHKF : 101
AgcF : ---------MLKSNKKLYYISAHKHAFEIDNLYPLNLFEGFVERIEKIEKTENCVLESSCKIDHDKLYPVRFNIGFPNN---SIKQLHAVMDFFRRVESRVDVKLNLSLFQQF : 101
AutF : ------MTTLLENSKKLYYIGIHKRVFEVENLYPLDVFENFVEQIEKT--SENCIIESSCKIEQDKLYPARFDIGFKLQ---NLEQLNAVYNFFQKVEARADVRVNHSLIQQF : 102
PatF : MDLIDRLQNNQRKDRRLQFVRTHQEAFDVKPTFPLPLFEEAILEI-----EGSCSVESSCQVEGDRLQGGRYEVQNN-QGTTWPESLTHAFKLLDKIDSQLGVRINRDSFDRF : 107
TruF2 : MVLSQLSKQTNLRENRLRCIRTHLEAFDIEPVLQISLFEEVIMEV-----EGSCNVKCSCKVERDRLFACQFTIAYS--QQKWPKTLKYNAILFDKIKSQVGICIDSSKFEQF : 106

PirF : LGHNFDFSKLEVLSAGLDLRTNLADSSLKIHIRIKD---YPEKINQALSLTIDGD-DLT--AVRDFLSVVGFIFYFDGRSAIFIYPEVKKEDF------FKPKTQEKVWQHLP : 206
PagF : LGSSFDFSKVTVLSAGIDLRNNLAESSLKMHIRIKD---YPEKLDKAFALSDGAA-DGN--YLKDFVNLIGFIFYFNGKSEIFIYAEVQEDDF------FKPEINNLVWQHFP : 208
TruF1 : LTLHQNSDKIEASLMGIDLRPNVKESSLKVHLRLDPQQDADELVMTAIDLDGGDYSPELTQVLLKDTFLIGFIFFLDGGSAVFMYTICPGKKPLAMLGKKGAYLKPYVLSNFS : 219
LynF : LAAHINSGKIMGISTGLDLRPELENSSVKIHIMLGE--NSEELVRTAIAIDGSHYPVELAQVLLKDTMMIGFIFFLNGHSEVFLYISCSRKKD-SLPNNRGESTRYYIRQKFS : 217
TolF : LAVHINSNKILWNTTGIDLRPNVEDSSVKIHIGIDPNQDTEELVMTAIGLDGSQYSPELIQVLLKDSYMIGFIFFLNGRSEVFLYTSCPGGKQ-QLVGNQGIYLKSYAKRNFS : 217
KgpF : LGADFDFSKILRNLVGVDARRELADSRVKIYIWMND---YPEKMATAMAWCDDKK--ELSTLIVNQEFLVGFIFYFDGRTAIFLYISLSSEEF------QQTQVWERLAKVVC : 206
AcyF : LGNNFDFSKVTKIVTGVDLREDFSASRLKEWFWIDN---YPEKLETASLLGDRE--DLRLLYVNNSWLVGFIFYLNGKSTIFLYPSMSKEES------QKVDVKLKLAKVLS : 203
AgcF : IGNDFKLDKMTDLMLGIDLRRDLSDSRLKIGLTIED---YPEKQKAAVILNNNID-EVTSNLLISNRLHIGFIFYLNGRSEMFLYPHIMQQDF------QKLDVQQRLSKVLS : 204
AutF : FGEDFDFSKMTGFMVGVDVRRELAESKLKIALTIKD---YPEKLKVAIALNGGLD-ETIQALMVSNSLHLGFILSLNGGSNIFLYLYIKKQEF------QQIHIQQRLAKVLS : 205
PatF : AAAHVNSRKIINNTIGVHLGSKLEDSSVMIYIHIKPEEDTEELARTALVLDGGRYSDELTRVLLRDTMVIGFLLFFDGRSRVILGPCAPGKSG-T-LKMKGKHLEQYTQKNLS : 218
TruF2 : SRLHVNSDKILDSTVGIDLRPKSQDSCIRISVHLEPKESPEELVRTALALDNATYTSELTQVFLQDCTAIIFECFFDGRSRIFLGAVAPGKKH-GFSGNHGRALTAYAQKYFS : 218

PirF : KFVLEPLQVTNLFGFGFSKTNHNPVVYYRLKGRQDLTNYFKINDTAQRVHSFYQHQDILPNMWVGTTQKELEKTRIENIRLYYYKSFKM------------- : 295
PagF : KTALQPLKASSLFFTGLSKANNNPVLYYHLKNRQDLTNYFKLNDTAQRVHSFYQHQDILPYMWVGTAQKELEKTRIENIRLYYYKSFKMESN---------- : 300
TruF1 : HKVTSLLQEVAALTVGFSKENPRPVLYFEFETLREVKYNLLFNSLGDKIYDFCLHNQIENFVSIGVTEPDLEKRRLENFRFYYRKAV--------------- : 306
LynF : PKVSSLLDASDFFVGGFSKANVEPVLYYAFENIKDIPKYFVFNDLGNRVYDFCRSQDSITMTWIGINERDLDRERLNNFRLYYRRSFG-------------- : 305
TolF : EKVFYLLEACDLFMAGFSKANTEAVIYFGFNNIEDMPKYFLFTSLGQRIYDFCRSQGAGPLPCVGVTQKDLESHHVENLRFYYRREFS-------------- : 305
KgpF : APALRLVNDCQAIQIGVSRANDSKIMYYHTLNPNSFIDNL-GNEMASRVHAYYRHQPVRSLVVCIPEQELTARS-IQRLNMYYCMN---------------- : 290
AcyF : APALNILDNFSSVLIGFTQTNQDRLIYGYLINPNEFIDNL-HNDIANRVHSFYRDKKVLNTMICFKEKELITGS-LDNFNLYYQMS---------------- : 287
AgcF : PPALQVVPACTRICVGISKANRDKIIYYYLENMGDFLNYFTVNDTARKVHAYYLKQPVVEMCVALPESELLAGTTIKNLNLYYLL----------------- : 289
AutF : TQALQPLPACSRICVGLSKANAEKVVYYYLENFNDFLNYFAANDTARRVHAYYREQPVKEMCVALQESELLAGT-IQKMNLYYLI----------------- : 289
PatF : RKVNSIFREGYLFGAFFSKTRVEPILFFYHSIIKDLPKYFTFNSLGDKIYNFCQSQGCITDVAIAVTETELEKSRLENFCFYYDQWDECKPSSDYDTERHLH : 320
TruF2 : PKAVSLSEVSDLFGMTISKYKAEPVLHFGFNNIKDISNYFLFNTLGNRIYSFCQNQDCILLAIIGVNEKELYSNRLENFLFDYAKNDESRMMRV--------- : 312

Figure S36: Multiple alignment of the following cyanobactin prenyl/geranyl transferases; (1) PirF: the *O*-geranyltransferase on tyrosine from piricyclamide biosynthesis (GenBank ID: AFK79989), (2) PagF: the forward *O*-prenyltransferase on tyrosine from prenylagaramide biosynthesis (GenBank ID: AED99429; PDB ID: 5TU6), (3) TruF1: the reverse *O*-prenyltransferase on serine and threonine from trunkamide biosynthesis (GenBank ID: ACA04492), (4) LynF: the reverse *O*-prenyltransferase on tyrosine from aestuaramide biosynthesis (GenBank ID: EAW34319), (5) TolF: the forward *O*-prenyltransferase on serine and threonine from tolypamide biosynthesis (GenBank ID: EKF00815), (6) KgpF: the *C*3 indole-prenyltransferase on tryptophan from kawaguchipeptin biosynthesis (GenBank ID: KXS89935), (7) AcyF: the *N*1 indole-prenyltransferase on tryptophan from anacyclamide biosynthesis (GenBank ID: AZB51087), (8) AgcF: the mono- and bis- *N* guanidine prenyltransferase on arginine from argicyclamide biosynthesis (GenBank ID: BCU11649), (9) AutF: the mono- N_{η} guanidine prenyltransferase on arginine from autumnalamide biosynthesis (GenBank ID: JAIGNI000000000), (10) PatF: the non-functional prenyltransferase from patellamide biosynthesis (PDB ID: 4BG2), (11) TruF2: the non-functional prenyltransferase from trunkamide biosynthesis (GenBank id: ACA04493). Positions of the residues at the substrate entrance site in PagF (PDB: 5TU6) are highlighted by a green star. Residues involved in binding of Mg ion and diphosphate group are highlighted by red triangle. Catalytic residue in PagF (Glu51) and the corresponding residues in other prenylases are highlighted by a blue triangle.

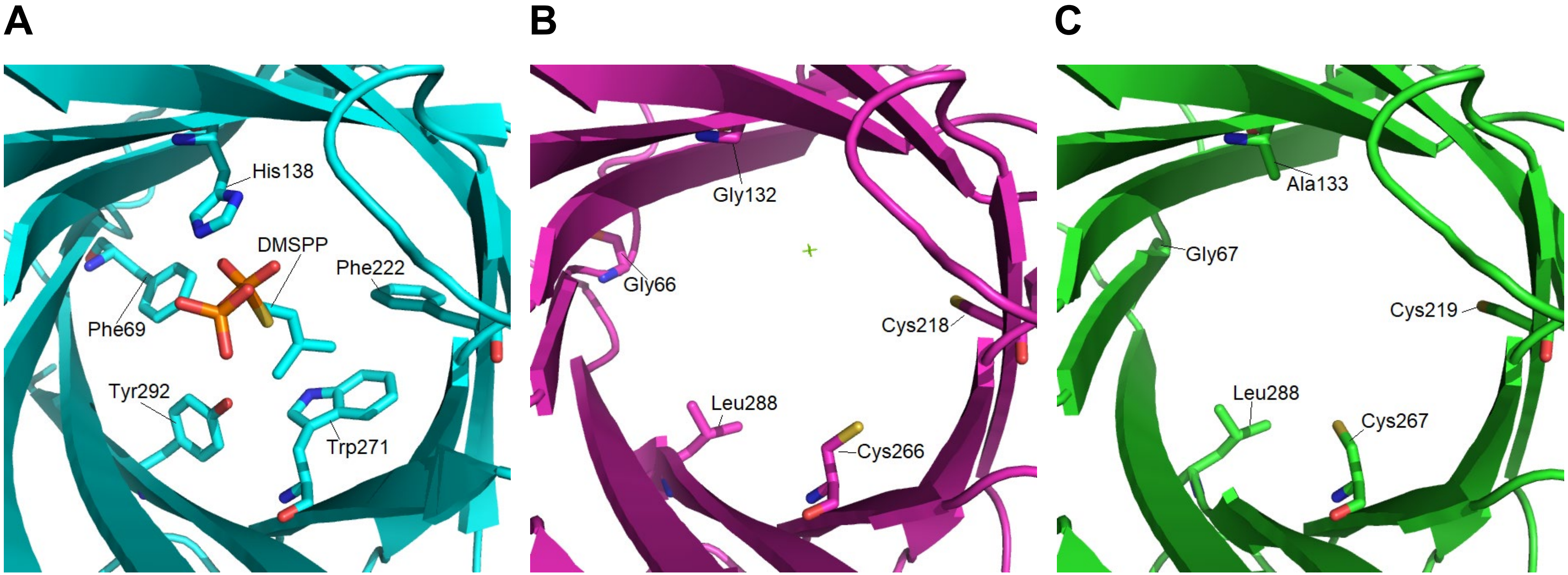

Figure S37: A) Crystal structure of the O-tyrosine prenyl transferase, PagF, showing the residues at the active site entrance and the DMSPP as sticks (PDB: 5TU6). Computational models of (B) AgcF and (C) AutF showing the corresponding residues at the active site entrance as sticks. Models were generated using SWISS-MODEL web tool[S16] using PagF structure (PDB 5TU6) as a template.
